# The Dual Face of Gingival Mesenchymal Stem Cell Paracrine Signalling in Oral Squamous Cell Carcinoma: A Pro-Tumour Transcriptional Programme and a Hypothesis-Generating Drug-Repurposing Screen

**DOI:** 10.3390/cells15171538

**Published:** 2026-08-26

**Authors:** Abdullah Alqarni, Jagadish Hosmani, Saeed Arem, Hussain Almubarak, Hassan Ahmed Assiri, Rayan Mohammedfarooq Meer, Shankargouda Patil

**Affiliations:** 1 Department of Oral Diagnosis, Oral Biology & Periodontology, College of Dentistry, King Khalid University, Abha 61421, Saudi Arabia; aawan@kku.edu.sa (A.A.); halmuawad@kku.edu.sa (H.A.A.); 2 Department of Diagnostic Science and Oral Biology, College of Dentistry, King Khalid University, P.O. Box 960, Abha 61421, Saudi Arabia; 3 Department of Preventive Dental Sciences, College of Dentistry, Taibah University, Madinah 42353, Saudi Arabia; rmeer@taibahu.edu.sa; 4 College of Dental Medicine, Roseman University of Health Sciences, South Jordan, UT 84095, USA

**Keywords:** cell–cell communication, circular analysis, drug repurposing, gingival mesenchymal stem cells, oral squamous cell carcinoma, paracrine signalling, proteogenomics, single-cell transcriptomics, spatial transcriptomics, TCGA-HNSC, tumour microenvironment

## Abstract

**Highlights:**

**What are the main findings?**

- Gingival mesenchymal stem cells (GMSCs), applied as conditioned medium or through indirect co-culture, raised proliferative and migratory oral cancer transcripts in every treatment arm, and the tumour-versus-normal direction of that same gene programme reproduces across three independent patient populations on three platforms—a cross-platform quality check, not patient-level evidence that GMSC signalling drives it. The functional read-outs did not follow; metabolic activity was unchanged, and wound closure was reduced under conditioned medium.
- Gefitinib, an approved epidermal growth factor receptor (EGFR) inhibitor, is nominated as a hypothesis-generating candidate against the pro-proliferation/migration programme, on the prior-motivated grounds that EGFR is raised in the tumour contrast and is a dependency in 69.8% (37 of 53) of oral-cavity squamous cell carcinoma lines; no activity of gefitinib was demonstrated in this system.

**What are the implications of the main findings?**

- Gingival mesenchymal stem cells (GMSCs), isolated from gum tissue, are an abundant cell source for regenerative dentistry, and their secretome raises a panel of pro-tumour transcripts in primary oral cancer cells, consistent with a shift towards a tumour-promoting transcriptional state; no corresponding functional shift was demonstrated in this system.
- This study describes candidate ligand–receptor routes for GMSC-driven pro-tumour transcriptional signalling and nominates gefitinib as a hypothesis-generating candidate—routes and a candidate that remain to be tested, since neither cell-line authentication nor a gefitinib experiment could be performed here.

**Abstract:**

**Background:** In our companion study, the gingival mesenchymal stem cell (GMSC) secretome suppresses oxidative stress and induces apoptosis in primary oral squamous cell carcinoma (OSCC) cells, where those wet-lab results are themselves reported as preliminary; here we ask whether it also engages a proliferation × migration programme in patient tissue. The two arms differ in read-out type (apoptosis and redox there, transcript abundance here), not in opposed function. **Methods:** Primary OSCC cells received GMSC-conditioned medium (GMSC-CM) or indirect Transwell co-culture, assayed by RT-qPCR (VEGFA, TGFB1, MMP9, CXCL12, CCND1, PCNA, MYC, EGFR), MTT, and scratch-wound migration. Thirteen computational layers plus a GeoMx spatial verify-and-decide layer were applied to public data: TCGA-HNSC, GEO, CPTAC, single-cell inference, prognostic modelling, DepMap and LINCS L1000. **Results:** All eight transcripts were raised in all three arms (16 of 24 comparisons significant), indicating a pro-tumour transcriptional shift; metabolic activity was unchanged and wound closure was reduced under conditioned medium (*p* < 0.01). Of 1358 genes significant in all three modalities, 1219 (89.8%, 95% CI 88.0–91.3%) share direction against 25.0% expected by chance (3.59-fold; exact binomial *p* below machine precision). The pre-registered oral-cavity signature did not validate externally (GSE41613; C = 0.562), and no ligand–receptor pair survived permutation calibration. **Conclusions:** GMSC paracrine exposure induces a pro-tumour transcriptional programme in primary OSCC cells that replicates at patient level, without demonstrated functional or therapeutic consequence; gefitinib is nominated only as a hypothesis-generating candidate. The evidence rests on three primary cultures (*n* = 3), one GMSC donor preparation, one 24 h time point, a single reference gene, no cell-line authentication and no test of gefitinib; the study programme has concluded, and these experiments cannot be performed now.

## 1. Introduction

### 1.1. OSCC Therapeutic Gap and the Rise in MSC-Based Regenerative Interventions

Oral squamous cell carcinoma (OSCC) accounts for roughly 90% of oral cancers and is the most common malignancy of the head-and-neck region [1,2]. Five-year overall survival in advanced disease has remained nearly 50% for the past two decades, despite improvements in surgical technique, intensity-modulated radiotherapy, and platinum-based chemotherapy [3]. Most OSCC arises in HPV-negative settings linked to tobacco and betel-quid use, with HPV-driven disease being largely confined to the oropharynx [4]. Targeted therapy options are limited, and adjuvant intervention is largely non-specific. These gaps have driven interest in regenerative-medicine adjuncts, including mesenchymal stem cells (MSCs) of various tissue origins, to support post-resection healing and to modulate tumour–stromal crosstalk [5,6].

### 1.2. Gingival MSCs and the Dual-Role Paradox

Gingiva-derived mesenchymal stem cells (GMSCs) have several practical advantages for oral applications. They reside in the same anatomic region as OSCC, can be harvested from minimally invasive biopsies, expand readily in vitro, and show lower immunogenicity than bone-marrow MSCs [7,8]. Their secretome is enriched in TGF-β family ligands, IGFs, FGFs, IL-6, MMPs/TIMPs, thrombospondins, fibronectin, collagens and extracellular vesicle cargo [9,10]. MSC–tumour interactions have long been described as biologically dual—protective on some pathways and tumour-promoting on others [11,12]. What has remained unclear is whether the same GMSC secretome, acting on the same OSCC cells, can suppress one cancer programme (apoptosis × redox) while activating another (proliferation × migration). In the companion study, now published in Int. J. Mol. Sci. [13], we describe the protective arm. The present paper describes the pro-tumour arm and places both in a single analytical framework.

### 1.3. The Proliferation × Migration Axis as a Paracrine-Driven OSCC Vulnerability

The eight-gene wet-lab panel used here (VEGFA, TGFB1, MMP9, CXCL12, CCND1, PCNA, MYC and EGFR) covers the canonical OSCC programmes of epithelial–mesenchymal transition (EMT; TGFB1, MMP9, CXCL12), extracellular matrix (ECM) remodelling (MMP9), proliferation (CCND1, PCNA, MYC, EGFR) and angiogenesis (VEGFA) [14,15,16,17,18]. Three questions follow. Does the GMSC secretome converge on this programme at the patient-cohort level? How does it engage stromal–tumour ligand–receptor signalling? And is the programme pharmacologically tractable, in the specific and limited sense that a compound whose transcriptional signature opposes it can be identified from public connectivity data and put forward for testing [13]?

### 1.4. Layered Framework with Explicit Anchor Exclusion

Our framework brings together cross-cohort transcriptomic prioritisation, primary-cell GMSC paracrine experimentation, patient-cohort consistency analysis, single-cell and spatial cell–cell communication inference, CRISPR dependency cross-referencing and a connectivity-based drug-repurposing screen. Throughout, the eight wet-lab genes are excluded from the analyses that could otherwise rediscover them: they are removed as seeds from the STRING shortest-path analysis (both source and target seed sets); removed from the ranking statistic and from gene-set membership in GSEA; and excluded from the feature pool of the LASSO–Cox prognostic model. This is a bias-control procedure of the kind long recommended in the circular-analysis and selection-bias literature [19,20,21,22], not a new statistical method, and we describe it as such (Section 2.2.12). It is also incomplete, and we quantify the residual: 10.0% (56 of 562) of the retained feature pool are STRING high-confidence direct partners of an excluded anchor, and two selected features are among them—AREG, an EGFR ligand, in PAN-HNSC, and MMP3, an MMP9 paralogue, in STRICT and BROAD. Anchor exclusion removes the measured genes; it does not remove their pathways.

We ran the companion apoptosis × ROS contrast alongside the present one so that the connectivity screen could be scored against both. We no longer present this as a source of axis selectivity. The two contrasts are the same tumour-versus-normal contrast computed on two heavily overlapping definitions of the same TCGA-HNSC oral-cavity cohort (Section 2.2.2) (Spearman ρ = 0.949 over 27,532 shared genes, rising to 0.992 among the 9885 genes significant in both), and no compound in the screen reaches FDR < 0.05 on the companion contrast (Section 3.10 and Section 4.5).

## 2. Materials and Methods

### 2.1. Experimental Methods

#### 2.1.1. Ethics

The study was conducted in accordance with the Declaration of Helsinki and approved by the Research Ethics Committee of King Khalid University (reference KKU-72-2025-21, dated 3 September 2025). Written informed consent was obtained from all tissue donors.

#### 2.1.2. Primary OSCC Cell Culture

Surgically resected OSCC tissue was used to generate primary OSCC cell cultures. Cells were maintained in Dulbecco’s modified Eagle’s medium (DMEM; Gibco/Thermo Fisher Scientific, Waltham, MA, USA) supplemented with 10% (*v*/*v*) foetal bovine serum (FBS; Gibco/Thermo Fisher Scientific) and 1% (*v*/*v*) penicillin–streptomycin (Gibco/Thermo Fisher Scientific) at 37 °C and 5% CO_2_ in a humidified incubator. Cultures were passaged at 80–90% confluence with 0.25% trypsin–EDTA (supplier not recorded), and functional assays were performed at passages 0–1, as documented for these same three patient-derived cultures in the companion study [13]. Epithelial identity rests on cobblestone morphology; no epithelial or stromal immunophenotyping was performed, so stromal contamination cannot be formally excluded [13], and no dedicated mycoplasma testing was performed [13]. The HPV status and the short-tandem-repeat (STR) profile of these cultures were not determined and cannot now be determined (Section Constraints on Experimental Re-Validation). Catalogue and lot numbers for the reagents named here and below cannot be retrieved.

#### 2.1.3. GMSC Isolation and Characterisation

**Figure 1:**
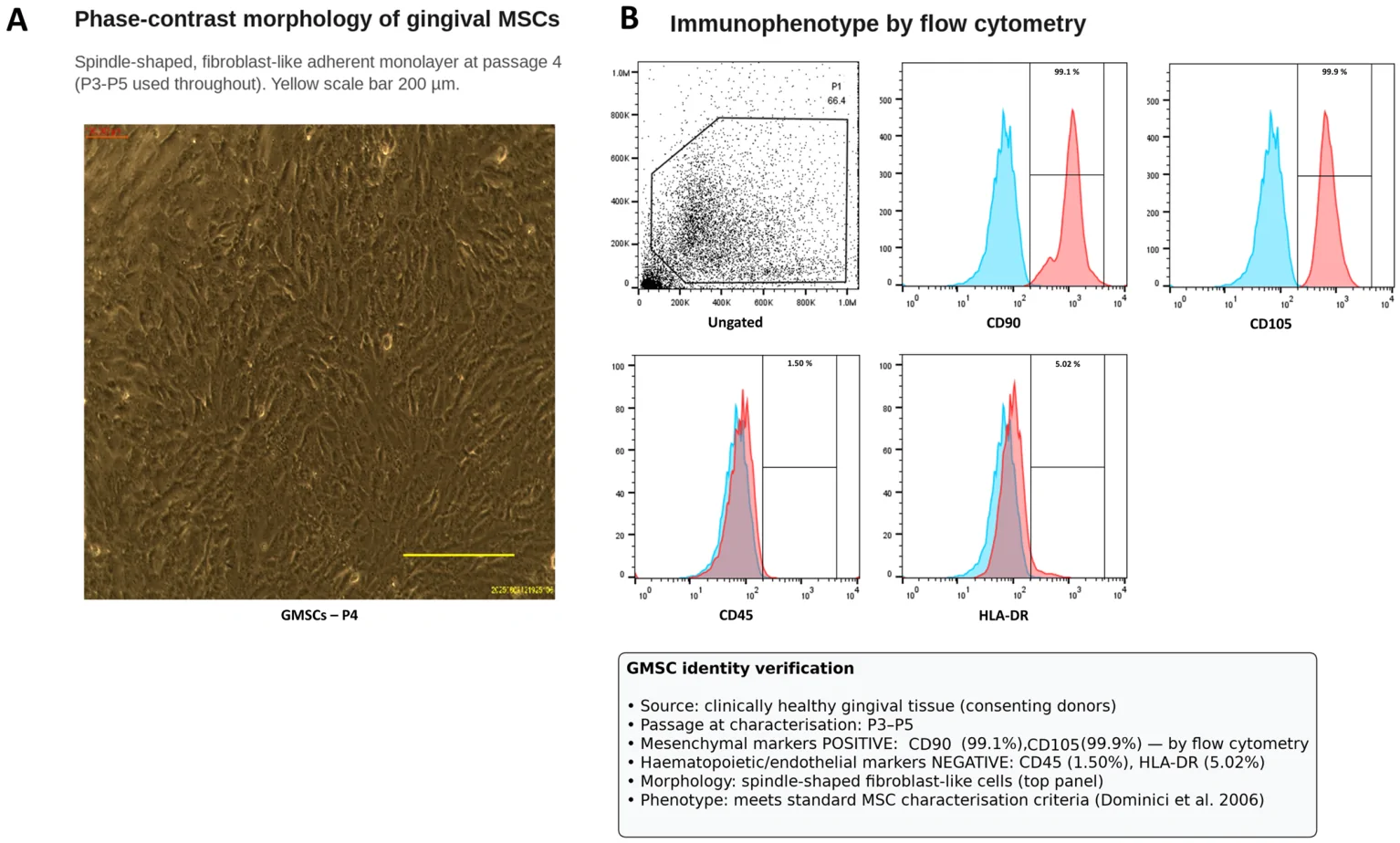
**GMSC characterisation.** Phase-contrast micrograph of gingival mesenchymal stem cells at passage 4 and flow-cytometric profiling of surface markers in the run shown here: CD90 99.1% and CD105 99.9% positive, CD45 1.50% and HLA-DR 5.02% (negative). The phenotype is consistent with an MSC population; the ISCT minimal criteria [23] are not met, because CD73 is 80.6% (below the 95% threshold) in the partly overlapping panel reported on the same cell system in the companion study [13], HLA-DR at 5.02% exceeds the ISCT ≤ 2% ceiling, CD14/CD11b and CD79a/CD19 were not assessed, and tri-lineage differentiation was not performed (Section 2.1.3). The two flow panels are not from the same acquisition and are reported separately rather than pooled (Section 2.1.3). Colour key for the flow-cytometry panels: the cyan trace is the unstained/isotype control and the red trace is the antibody-stained sample; where the two distributions coincide (CD45, HLA-DR) the traces overlap and appear brown. The vertical line marks the positivity gate and the boxed value gives the percentage of events above it. The scale bar in the micrograph is yellow. Scale bar: 200 µm.

Gingival tissue was harvested during routine periodontal procedures from a consenting healthy donor and processed under sterile conditions. A single GMSC donor preparation was used throughout this study and throughout the companion study, so GMSC donor-to-donor secretome variation is not sampled [13]. GMSCs were expanded in DMEM + 10% FBS + antibiotics. The morphology of the GMCs were spindle sphaped, fibroblast like adherent monolayer seen in phase contrast microscope at passage 4 shown in Figure 1.

In the flow-cytometric run shown in Figure 1, the cells were 99.1% positive for CD90 and 99.9% positive for CD105, and negative for CD45 (1.50%) and HLA-DR (5.02%), with spindle-shaped fibroblast-like morphology on phase-contrast microscopy. The per-marker assignment is taken from the source acquisition figure retained by the laboratory (Figure 1.tif, the file from which Figure 1B is drawn), which labels its CD90 tile at 99.1% and its CD105 tile at 99.9%, and the manuscript. The assignment stated here is the one the source figure carries, read tile by tile; it is not inferred from any other source. A second, partly overlapping panel on GMSCs from the same laboratory is reported in the companion study—CD73 80.6%, CD90 98.9%, CD34 1.60%, CD45 2.41%, with CD105 not assessed in that run [13]. The two panels are not the same acquisition: each reports two markers; the other does not, and the two shared markers differ (CD90 99.1% vs. 98.9%; CD45 1.50% vs. 2.41%). The corrected assignment happens to bring our CD90 value closer to the companion’s than the transposed one did, and we note that explicitly so that it cannot be read as the reason for the correction: the correction rests on the tile labels in our own source figure, and would stand unchanged had the companion never been published. The antibody clones and isotype controls, the cytometer and the analysis software used for our run are not recorded, and the raw FCS files were not retained by the outsourced facility [13]; the companion’s published flow schedule describes a different acquisition and has therefore not been adopted here (Section Constraints on Experimental Re-Validation). The two panels cannot be reconciled retrospectively, and we report each as acquired rather than pooling or averaging them. On the union of the two panels, the International Society for Cellular Therapy minimal criteria [23] are not met: CD73 at 80.6% is below the 95% threshold, HLA-DR at 5.02% exceeds the ≤2% ceiling, CD14/CD11b and CD79a/CD19 were not assessed, and tri-lineage differentiation was not performed. We therefore describe the cells as GMSC-like primary gingival stromal cells that are mesenchymal-consistent rather than ISCT-compliant.

#### 2.1.4. GMSC-Conditioned-Medium (GMSC-CM) Preparation

**Figure 2:**
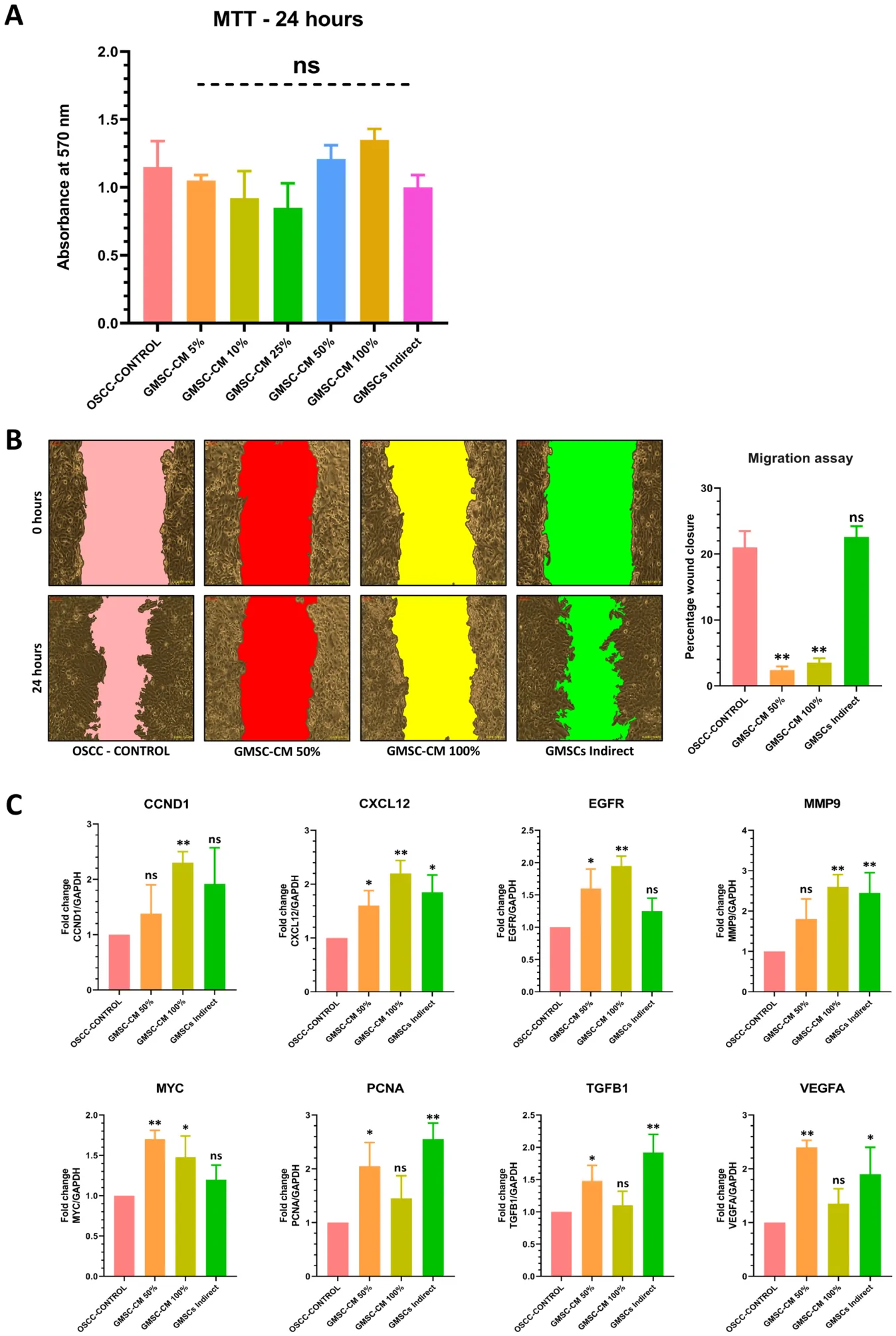
**Wet-lab functional and transcriptional read-outs after GMSC-conditioned medium and Transwell co-culture treatment.** (**A**) MTT metabolic activity (absorbance at 570 nm) at 24 h across seven arms (OSCC control; GMSC-CM 5%, 10%, 25%, 50%, 100%; GMSCs indirect/Transwell). No comparison between arms reached significance: the span bracket is annotated ns. (**B**) Wound-healing scratch migration at 0 h and 24 h, with representative micrographs (shaded overlay marks the unclosed wound area; fill colour identifies the arm and is not a measurement) and percentage closure. Closure is significantly reduced under GMSC-CM 50% and GMSC-CM 100% (both *p* < 0.01); the serum-matched Transwell arm does not differ from the OSCC control (ns). (**C**) RT-qPCR for the eight-gene panel (2^−ΔΔCq^, GAPDH reference, OSCC control = 1). All eight transcripts are raised above control in all three arms; 16 of the 24 comparisons are significant (9 at *p* < 0.01, 7 at *p* < 0.05) and 8 are not significant. Significance key, applied to all panels: ns, not significant; * *p* < 0.05; ** *p* < 0.01 (one-way ANOVA with Tukey’s post hoc test). n = 3 biological replicates. Bars, error bars and micrographs are carried over unaltered from the source figures; no numeric value has been re-derived (Section Constraints on Experimental Re-Validation).

GMSCs at ≈80% confluence were rinsed twice in phosphate-buffered saline (PBS) and switched to serum-reduced DMEM for 24 h. The harvested supernatant was centrifuged (300× *g*, 5 min, 4 °C) to remove cellular debris, sterile-filtered through a 0.22-μm syringe filter, aliquoted, and stored at −80 °C. Working dilutions were prepared in fresh DMEM at 5%, 10%, 25%, 50%, and 100% (*v*/*v*); the metabolic-activity assay used the full series, and the migration and RT-qPCR read-outs used GMSC-CM 50% and GMSC-CM 100% (Figure 2). Each batch was used within 24 h of preparation. Because the conditioning step is performed in serum-reduced DMEM, the GMSC-CM arms are serum-depleted relative to the 10% FBS control, whereas the Transwell arm is not; this asymmetry is not a design choice we can now correct, and its interpretive consequence is addressed in Section 3.1 and Section Constraints on Experimental Re-Validation.

#### 2.1.5. Indirect Transwell Co-Culture

GMSCs were seeded in the upper chamber of a 0.4-μm pore Transwell insert (Corning Incorporated, Corning, NY, USA), which permits soluble-factor exchange while preventing direct cell–cell contact, and primary OSCC cells in the lower well. Co-cultures were maintained for 24 h before downstream assays. This arm is maintained in the same serum-containing medium as the control and is therefore the only serum-matched treatment.

#### 2.1.6. MTT Metabolic-Activity (Cell-Viability) Assay

Following 24 h treatment with the GMSC-CM dilution series or Transwell co-culture, OSCC cells were incubated with MTT reagent (thiazolyl blue tetrazolium bromide) for 4 h. Formazan was solubilised in dimethyl sulfoxide, and absorbance was measured at 570 nm on a microplate reader. The read-out is metabolic activity of viable cells, which reflects viable-cell number and per-cell metabolic state jointly and is not a proliferation-specific measurement; it is reported as such throughout. Data are absorbance at 570 nm, n = 3 biological replicates.

#### 2.1.7. Wound-Healing Migration Assay

A confluent OSCC monolayer was scratched with a sterile 200-μL pipette tip, washed in PBS, and treated with GMSC-CM 50%, GMSC-CM 100%, or maintained under indirect Transwell co-culture. Phase-contrast images were captured at 0 h and 24 h on an inverted microscope; wound area was quantified in Fiji/ImageJ v1.54t (National Institutes of Health, Bethesda, MD, USA; open source)and percentage closure was reported relative to OSCC control. The make and model of the inverted microscope were not recorded and cannot now be retrieved (Section Constraints on Experimental Re-Validation).

#### 2.1.8. RT-qPCR

Total RNA was extracted with TRIzol reagent (Invitrogen/Thermo Fisher Scientific, Waltham, MA, USA) and DNase-treated, and RNA purity was assessed by A260/A280 absorbance ratio, as documented for the same workflow in the companion study [13]. Complementary DNA was synthesised from 1 μg total RNA using the RevertAid First-Strand cDNA synthesis kit (Thermo Fisher Scientific, Waltham, MA, USA). SYBR Green Master Mix (Thermo Fisher Scientific) [13] was used for the eight-gene panel (VEGFA, TGFB1, MMP9, CXCL12, CCND1, PCNA, MYC, EGFR) with GAPDH as the single reference gene. Relative expression was computed by the 2^−ΔΔCq^ method. Each condition was assayed in biological triplicate (n = 3). As documented for the same cell systems in the companion study [13], the three biological replicates are three independent patient-derived primary OSCC cultures (one tumour per donor), each assayed in technical triplicate, with the donor as the unit of analysis; the conditioned-medium and Transwell arms derive from a single GMSC donor preparation applied across all three cultures, so GMSC donor-to-donor secretome variation is not sampled. Per-donor clinical metadata (stage, site, sex, age) were not retained by the outsourced facility and cannot now be recovered [13] (Section Constraints on Experimental Re-Validation).

Against the MIQE checklist [24], the following items are recoverable and are stated above: RNA extraction and DNase treatment, RNA purity assessment, cDNA synthesis kit and RNA input, detection chemistry, reference gene, quantification method, primer sequences (Table 1) and replicate structure. The following are not recoverable and cannot now be generated: reaction composition and volume, cycling conditions, instrument model, melt-curve and no-template-control records, amplification efficiencies from standard curves, and any formal test of GAPDH stability across the treatment arms. Amplicon lengths and per-assay RefSeq accessions are printed in Table 1; per-assay RefSeq accessions were not retained. Two consequences follow and are discussed in the Limitations Section. First, 2^−ΔΔCq^ assumes comparable amplification efficiencies between target and reference; that assumption was verified only in silico and not experimentally. The in silico check is reported in full: every pair amplifies a single product in its intended gene, no pair yields a product in any other human RefSeq transcript, and for each primer the best BLAST alignment against human RefSeq RNA is to its intended gene. Specificity was confirmed by BLASTN (task blastn-short) against the NCBI RefSeq RNA database restricted to Homo sapiens (taxid:9606), run through the NCBI BLAST web service (accessed 21 May 2026) If a local install was used instead, write NCBI BLAST+ v2.17.0 in place of “the NCBI BLAST web service (accessed 21 May 2026)”. Two loci align to a single primer at equal score—an uncharacterised GAPDH-related transcript (XR_007059630.1) and the antisense RNA SLC12A5-AS1/MMP9-AS1 (NR_147699.1)—but neither can be amplified, because only one primer of the pair binds each. Specificity is therefore supported; amplification efficiency is not, and that is what the assumption 2^−ΔΔCq^ actually requires. Second, GAPDH stability under GMSC-CM exposure was not assessed, so if GAPDH itself shifted, the fold-changes would shift with it. The panel should therefore be read as an internally consistent set of relative, direction-bearing measurements rather than as absolutely calibrated fold-changes.

#### 2.1.9. Wet-Lab Statistical Analysis

Group comparisons (n = 3) used one-way ANOVA with Tukey’s post hoc test in GraphPad Prism v10.4 (GraphPad Software, Boston, MA, USA); *p* < 0.05 was considered significant. The significance tiers annotated in Figure 2 are ns (not significant), * *p* < 0.05; ** *p* < 0.01. Because the underlying absorbance values, wound areas and Cq values for this study’s eight-gene panel and functional assays are no longer available to us (Section Constraints on Experimental Re-Validation), which also sets out the scope of the companion study’s own availability statement [13]), this manuscript reports only the direction and significance tiers licensed by the original figure annotations. Consequently, any mean or standard error values that cannot be traced back to a source file are omitted.

### 2.2. Computational Methods

#### 2.2.1. Rationale for the Layered Design

The computational pipeline comprises 13 analytical layers (L1–L10 and L12–L14), with L15 spatial validation as a verify-and-decide layer. Layer L11 (foundation-model in silico perturbation) is not used (Section 4.7); the layer numbering retains the gap so that all deposited outputs and code references remain addressable. A consolidated layer-to-figure map is given in Table 2, and Table 3 states, for each layer, what data it consumes, what it shares with other layers, and what it can and cannot establish.

**Table 2:** Computational layer summary. Each layer maps to a deposited output, a method or tool, and the resulting figure panel(s). “AE?” flags layers to which explicit anchor exclusion is applied; n/a indicates that the eight wet-lab genes are not used as analytical inputs to that layer at all. Five of the thirteen analytical layers apply explicit anchor exclusion (L4 seeds, L5 MODE-A, L7 method iii, L9 ranking statistic and gene-set membership, L12 queries). The table has 15 rows: the 13 analytical layers, the L10b matrix-decomposition sub-layer, and the L15 verify-and-decide layer.

Layer	Method/Tool	Public Dataset	Key Output	Figure(s)	AE?
L1	PyDESeq2 0.5.4 [25,26]	TCGA-HNSC GDC v45.0 [27]	Tumour-vs.-normal DE (STRICT/BROAD/PAN_HNSC)	Figure 3A and Figure S1A	n/a
L2	PyMARE REML v0.0.10 [28]	GSE30784 [29] + GSE37991 [30]	Cross-cohort meta DE with τ^2^, I^2^	Figure 3B and Figure S2A–H	n/a
L3	CPTAC 1.5.14 [31,32]; Welch’s t	CPTAC HNSCC [31,32]	Protein DA, anchor concordance	Figure 4A–C and Figure S1C	n/a
L4	networkx 3.6.1 shortest-path; STRING v12.0 [33]	STRING v12.0 [33]	Anchor-blind PPI pairs, 200-rewire null	Figure 5C	Yes (seeds)
L5	NicheNet NSGA-II prior [34]; liana-py 1.7.1 [35]	NicheNet Zenodo [34]; LIANA consensus	Ligand activity (MODE-A/MODE-B)	Figure 5A,B	Yes (MODE-A)
L6	scanpy 1.11.5 [36]; harmonypy 2.0.0 [37]; leidenalg 0.11.0	GSE103322 [38]	UMAP + Leiden + DPT/PAGA pseudotime	Figure 6A–F	n/a
L7	scikit-survival 0.27.0 [39]; lifelines 0.30.3 [40]	TCGA-HNSC + Liu et al. [41]; GSE41613 [42] for external validation	LASSO–Cox signature; KM + Cox, CV, permutation nulls; external application of frozen coefficients	Figure 7A–C and Figure S7A–H	Yes (method iii)
L8	PROGENy v2 [43]; CollecTRI [44]; decoupler-py 2.1.6 [45]	OmniPath snapshot 25 July 2026	Pathway + TF activity, primary tumours only	Figure 8A,B	n/a
L9	gseapy.prerank 1.2.1 [46]; Enrichr libraries [47,48,49,50,51]	Hallmark/KEGG/Reactome/GO-BP	NES across libraries	Figure 8C and Figure S6A,B	Yes (rank + sets)
L10	LIANA consensus [35] (CellPhoneDB [52], NATMI [53], Connectome [54])	GSE103322 [37]	Fibroblast → malignant LR ranking	Figure 9A and Figure S3A	n/a
L10b	Truncated SVD, substituted for cell2cell 0.8.4 [55]	GSE103322 [37]	Patient × component decomposition	Figure 9B,C and Figure S3B	n/a
L12	gseapy.Enrichr 1.2.1 [45]; LINCS L1000 [56,57]; DGIdb v5 [58]	LINCS L1000; DGIdb v5	Dual-axis selectivity, FDA/ATC/target annotations	Figure 10A–C and Figure S4	Yes (queries)
L13	decoupler-py 2.1.6 ULM [44]	Curated 17-cell-type panel (13 scored)	Immune-compartment stratification	Figure 8D	n/a
L14	DepMap 24Q4 Chronos [59]	DepMap 24Q4	Per-gene essentiality across 53 oral-cavity SCC lines (77 head-and-neck lines, sensitivity), annotated with the Common Essential flag and the all-lineage median (Table 4)	Figure 11A and Figure S5A,B	n/a
L15	NanoString GeoMx + custom DCC parser [60]	GSE300414 [60]	Stromal-ligand × tumour-receptor Spearman on Q3-normalised counts, 153 pairs, 24 patients, with a 10,000-draw permutation null	Figure 11B–D	n/a

The cross-modality convergence analysis (Section 3.3, Figure 4D–F) is not a new layer: it is a re-analysis of the L1, L2 and L3 outputs.

**Table 3:** Layer independence and inferential scope. “Shares data with” lists the other layers that consume the same primary matrix or the same curated candidate list; layers sharing an entry are not independent tests of one another.

Layer	Primary Data Source	Shares Data with	Can Establish	Cannot Establish
L1	TCGA-HNSC RNA-seq (tumour + normal)	L7, L8, L9, L12, L13	That the panel’s direction is reproduced in oral-cavity HPV-negative tumours vs. normals	Causation, mediation, or prognosis
L2	GSE30784 + GSE37991 microarray	—	Independent-population replication of effect direction on a second platform	Prognosis (neither cohort carries outcome data)
L3	CPTAC HNSCC proteomics	—	That the direction survives to the protein level in a third population	Oral-cavity specificity (the cohort is HNSCC-wide)
L4	STRING v12.0 physical interactions	L5, L10, L15 (shared 46-ligand list)	That short, high-confidence paths exist from secretome ligands to non-anchor targets	That those paths are used in this system
L5	NicheNet NSGA-II prior	L4, L10, L15 (shared list)	Ligand–target regulatory potential ranking over the candidate space	Independence from prior literature, which includes MSC studies
L6	GSE103322 scRNA-seq	L10, L10b	An EMT-ordered malignant compartment in HNSCC tissue	Any GMSC-specific effect (no GMSCs in the atlas)
L7	TCGA-HNSC VST, primary tumours; GSE41613 for external validation	L1, L8, L9, L12, L13 (fitting cohorts only; GSE41613 shares no sample with any layer)	Whether a signature carries out-of-fold prognostic information, and whether the frozen signature transfers to an independent cohort	A risk-group claim (no median split is significant), and oral-cavity prognostic validity; the variant that validates externally was fitted out of scope (Section 3.6 and Section 4.4)
L8	TCGA-HNSC VST, primary tumours	L1, L7, L9, L12, L13	Pathway/TF activity differences between anchor-score strata	Direction of causation between programme and activity
L9	TCGA-HNSC DE ranking	L1, L7, L8, L12, L13	Which gene sets are enriched with the anchors removed	Independence from L1, which supplies the ranking
L10	GSE103322 scRNA-seq	L6, L10b, L15	Ranked fibroblast → malignant ligand–receptor candidates	That the pairs are active, or that fibroblasts equal GMSCs
L10b	L10 score matrix	L10	Dominant variance structure across 12 patients	Latent communication programmes (component 1 is a scale term)
L12	TCGA-HNSC DE top lists; LINCS L1000	L1, L7, L8, L9, L13	Which catalogued perturbation signatures oppose the tumour signature	Drug activity, selectivity, or effect in this system
L13	TCGA-HNSC VST, primary tumours	L1, L7, L8, L9, L12	Marker-based immune-compartment activity trends between strata	Cell abundance (ULM scores activity, not fractions)
L14	DepMap 24Q4 Chronos	—	That nominated target genes are dependencies in oral-cavity SCC lines, and whether that dependency is selective for the lineage or common to almost all screened lines	That a drug against them reverses the GMSC effect
L15	GSE300414 GeoMx	L10 (candidate space), L4/L5 (ligand list)	Whether L10-predicted pairs co-localise across AOIs in 24 patients	Independent validation: its candidate space is L10’s output

**Table 4:** DepMap Public 24Q4 essentiality of the twelve genes crossing the −0.5 threshold in the 53 oral-cavity SCC lines, annotated for lineage relevance. “Common Essential” is the release’s own flag, read from CRISPRInferredCommonEssentials.csv. Selectivity is the oral-cavity median minus the median across the 1178 screened lines; a more negative value means the dependency is stronger in oral-cavity SCC lines than in cancer cell lines generally. Essentiality is median Chronos < −0.5. normLRT is our reimplementation of the statistic behind DepMap’s Strongly Selective label, computed with the Jones–Faddy skew-t rather than the Azzalini skew-t used by the portal (Section 2.2.11); it is given for reference only, and it measures skewness of the global dependency profile rather than oral-cavity specificity. Rows are ordered by oral-cavity median; EGFR is set in bold. The same annotation for all 88 measured genes is deposited.

Gene	Common Essential (24Q4)	Median Chronos, Oral Cavity (n = 53)	Median Chronos, All Lineages (n = 1178)	Selectivity (Oral − All)	Essential, Oral Cavity	Essential, All Lineages	normLRT (Reimplemented)
*PCNA*	Yes	−2.864	−2.750	−0.115	100.0% (53/53)	99.9%	50.0
*MYC*	Yes	−1.996	−1.819	−0.177	98.1% (52/53)	94.8%	66.0
*MTOR*	Yes	−1.137	−1.168	+0.031	98.1% (52/53)	98.6%	26.3
*CRKL*	Yes	−1.120	−0.812	−0.308	94.3% (50/53)	74.9%	232.8
*EIF4A1*	Yes	−1.094	−1.242	+0.149	98.1% (52/53)	94.6%	5.0
*CDK6*	No	−0.839	−0.505	−0.335	77.4% (41/53)	50.5%	208.7
*CCND1*	No	−0.821	−0.845	+0.024	81.1% (43/53)	64.9%	131.6
***EGFR***	**No**	**−0.677**	**−0.189**	**−0.488**	**69.8% (37/53)**	**19.0%**	652.2
*NUP62*	Yes	−0.634	−0.587	−0.047	69.8% (37/53)	65.0%	50.8
*CDK4*	Yes	−0.579	−0.618	+0.039	62.3% (33/53)	66.7%	569.1
*PIK3CA*	No	−0.553	−0.426	−0.127	56.6% (30/53)	39.0%	316.6
*RELA*	No	−0.549	−0.368	−0.181	50.9% (27/53)	26.7%	179.3

The design answers a specific problem. A primary-cell perturbation across three donor cultures at n = 3 can indicate that a set of transcripts moves relative to a reference gene; it cannot establish that the movement is relevant to human disease, that it is mediated by any particular ligand, or that it is actionable. Each layer therefore addresses one of those gaps with data the wet-lab experiment does not contain, and the layers are ordered so that the question asked of each is answerable by the data it uses: L1–L3 ask whether the programme is present in patient tumours, on three platforms and in three disjoint populations; L4, L5 and L10 ask which secreted ligands could plausibly deliver it, over one curated candidate space; L6 places it on a single-cell EMT trajectory; L7 asks whether it carries prognostic information; L8, L9 and L13 characterise the pathway, transcription-factor and immune-compartment context of the high-anchor stratum; L12 asks whether any catalogued compound’s transcriptional signature opposes it; L14 asks whether the nominated targets are genuine dependencies; and L15 asks whether the inferred ligand–receptor pairs co-localise in tissue.

Two properties of this design must be stated at the outset, because they bound every conclusion drawn from it. First, the layers are not statistically independent of one another. L4, L5, L10 and L15 all draw on a single pre-registered 46-ligand curated GMSC secretome, so agreement between them is agreement about coverage of a shared list; L15’s entire candidate space is L10’s output; and L1, L7, L8, L9, L12 and L13 all derive from the same TCGA-HNSC expression matrix. Table 3 makes the sharing explicit. Second, layer count is not evidence. Thirteen layers applied to overlapping data do not constitute thirteen independent tests, and we no longer present the framework’s breadth as if it were.

#### 2.2.2. Public Datasets and Provenance Pinning

- TCGA-HNSC harmonised STAR-Counts and clinical metadata were retrieved from the Genomic Data Commons API under Data Release 45.0 (December 2025) [27]. Oral-cavity restriction was applied by ICD-10 prefix (C00, C02–C06; excluding C01, C05.1, C07–C10, C13, C32). HPV-negative status was confirmed against the TCGA Network molecular classification [61] through the cBioPortal Datahub mirror of Liu et al.’s PanCanAtlas Clinical Data Resource [41,62]. Applying only the anatomic restriction to our own sample table returns 194 primary tumours, one metastatic sample and 15 normals (the same single metastatic sample and the same 15 normals as the companion reports) and adding the molecular HPV-negative requirement gives the 171 primary tumours and 13 normals used throughout this paper.
- GEO bulk OSCC microarray cohorts were retrieved from NCBI GEO [63] as series-matrix files: GSE30784 (Chen et al., HG-U133 Plus 2.0; 167 tumour/45 normal) [29] and GSE37991 (Lee et al., GPL6883 Illumina HumanRef-8 v3.0; 40 tumour/40 normal) [30]. GSE25099 was excluded after acquisition because its platform (Affymetrix Human Exon 1.0 ST, GPL5175) lacked a usable probe-to-gene mapping in our cache; the companion study included this series in its own three-cohort meta-analysis [13], so the exclusion reflects our cache state rather than a property of the series (Section 4.7). Neither series carries outcome data, which is why the external prognostic test uses a third deposit.
- External prognostic cohort: GSE41613 (Lohavanichbutr et al., GPL570 Affymetrix HG-U133 Plus 2.0; 97 HPV-negative oral-cavity SCC with per-patient follow-up, 51 deaths, median follow-up 54.4 months) [42], the series matrix was downloaded (md5 ad645ea2d7fac58a141cc1e9756ca0d5) on 27 July 2026. It shares no samples with any other layer and is used once, with frozen coefficients (Section 2.2.6).
- CPTAC HNSCC umich proteomics (Report_abundance_groupby = protein, median-centred normalisation) was obtained through the PNNL cptac Python package v1.5.14 [26,27] for 116 tumours and 66 normal-adjacent samples. The cohort is HNSCC-wide and is not restricted to the oral cavity; this scope limit is carried into Section 4.7.
- Single-cell RNA-seq: GSE103322 (Puram et al., SMART-seq2) [38]: The deposited matrix contains 5902 cells; 5891 remain after our gene-count filter, and it is the latter number that is analysed throughout Section 3.5. Donor assignment is taken from the cell-barcode prefix and yields 17 batch labels (16 patient identifiers and one unassigned group), which is the key supplied to Harmony; the prefix is not a clean patient key in this deposit (it carries two parallel naming conventions for the same cases and a generic token), so it is used as a batch covariate and not as a patient count. The patient count for this cohort is the one given in the source publication: 18 HNSCC patients [38].
- Spatial transcriptomics: GSE300414 (Ourailidis et al. 2026) [60]. The deposit comprises 24 HPV-negative HNSCC patients, 133 ROIs/289 tissue AOIs parsed from 293 DCC files plus 4 no-tissue negative controls, of which 276 AOIs pass QC. The deposit corresponds to the 24 tumour-budding patients of the published 43-patient series (24 budding/19 non-budding); the 19 non-budding patients are not deposited. The manuscript previously described this dataset as 43 patients and 293 ROIs, which is the published series rather than the deposit actually analysed.
- STRING v12.0 high-confidence physical interactions (combined_score ≥ 700; 173,038 edges after filtering, 19,699 nodes) [33].
- NicheNet ligand–target regulatory potential matrix (NSGA-II final v2; 33,354 targets × 1226 ligands) retrieved from Zenodo [34] and parsed via pyreadr.
- DepMap Public 24Q4 CRISPR Chronos gene-effect scores (CRISPRGeneEffect.csv), the Common Essential gene list (CRISPRInferredCommonEssentials.csv), and Model.csv were retrieved from figshare article 27993248 [59]. Cell-line panel membership is derived from Model.csv on Oncotree annotation only; no cell-line identifiers are hard-coded (see Section 2.2.10 and Section 3.11).
- LINCS L1000 chemical-perturbation signatures [56,57] were queried via the Enrichr API [54] (accessed 21 May 2026; Enrichr is not versioned, so the access date is the provenance record) using gseapy v1.2.1 [39]. All API responses were cached as dated JSON.
- DGIdb v5 drug–gene annotations were queried via the GraphQL endpoint https://dgidb.org/api/graphql (accessed on 21 May 2026) [58]; responses were cached as dated JSON.
- Gene-set libraries were accessed through the Enrichr endpoint [48] under the library names MSigDB_Hallmark_2020, KEGG_2021_Human, Reactome_Pathways_2024 and GO_Biological_Process_2023 [47,48,50,51,64], at a recorded fetch date. No MSigDB release pin is recorded for these libraries; the Enrichr fetch date is the provenance record.
- PROGENy (top-500 footprint genes per pathway) [43] and CollecTRI [44] were snapshotted from OmniPath on 25 July 2026 (PROGENy 6463 rows/14 pathways; CollecTRI 42,990 rows/1185 TFs, of which 750 were scored after decoupler min_npruning), with SHA-256 recorded in the deposited OMNIPATH_SNAPSHOT.json; OmniPath/SIGNOR signed-direction overlay [65,66] was applied.
- Curated GMSC secretome (v1.0; 46 factors with per-row PMID provenance) is deposited with the code.

#### 2.2.3. Target Prioritisation (Layers L1, L2, L3, L4)

**Figure 3:**
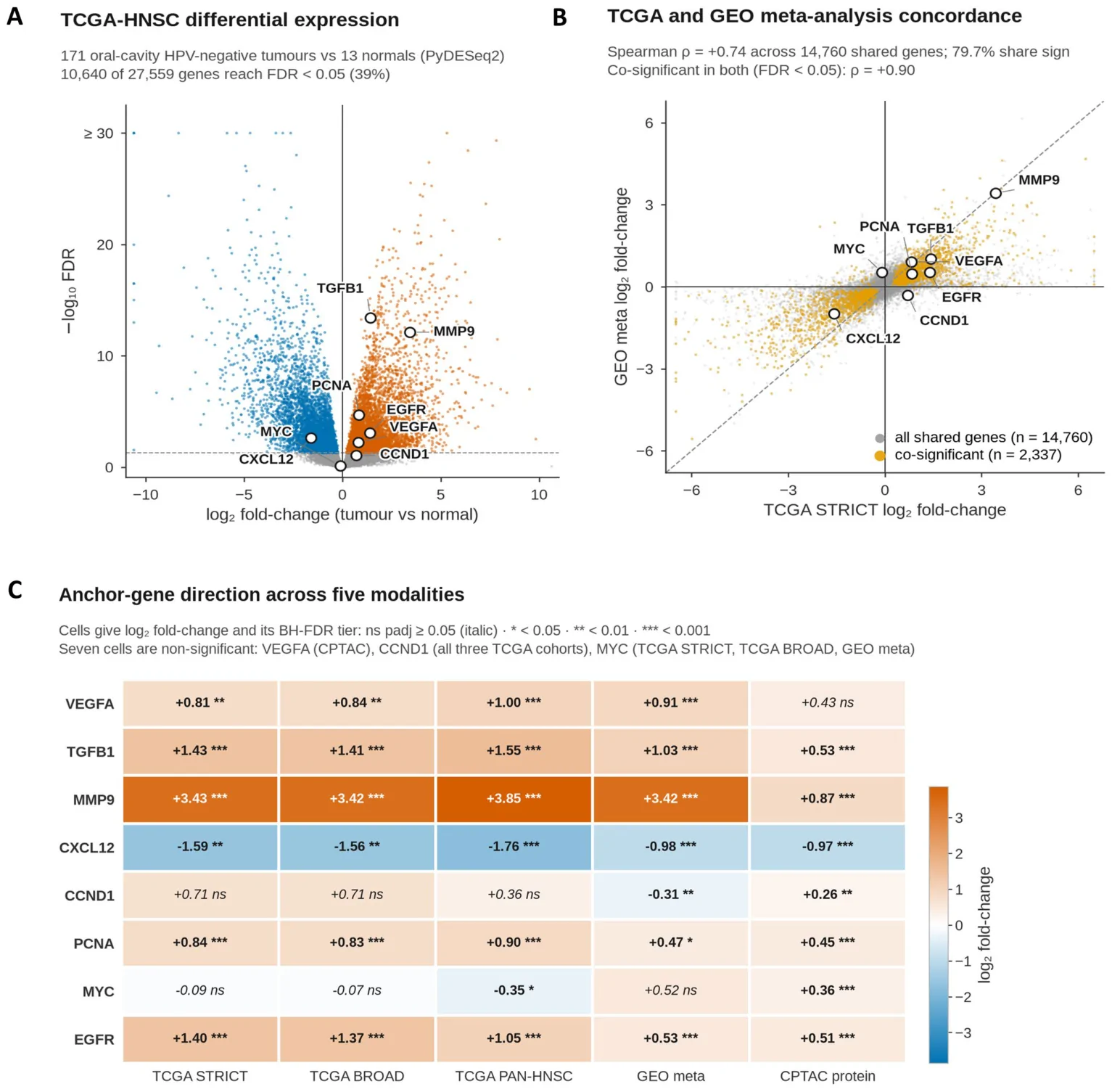
**TCGA-HNSC differential expression and GEO meta-analysis concordance.** (**A**) Volcano plot of TCGA-HNSC STRICT tumour-vs-normal differential expression (PyDESeq2; n = 171 tumours/13 normals; 27,559 genes; 10,640 significant at padj < 0.05), with the eight anchors highlighted. (**B**) Cross-cohort concordance: TCGA STRICT log_2_FC versus GEO REML meta log_2_FC. Spearman ρ = +0.738 across the 14,760 genes shared by the two tables, 79.7% of which share sign, rising to ρ = +0.896 among the 2337 genes co-significant at padj < 0.05 in both (Pearson r = 0.84 on that subset). The agreement is insensitive to where the co-significance threshold is set: relaxing the GEO criterion to padj < 0.10 admits 2665 genes and gives ρ = +0.893 and Pearson r = 0.84. (**C**) Anchor-gene direction across five modalities (TCGA STRICT/BROAD/PAN_HNSC, GEO meta, CPTAC protein). Each cell prints the log_2_ fold-change with its BH-FDR tier: ns padj ≥ 0.05 (italic), * padj < 0.05, ** padj < 0.01, *** padj < 0.001. Seven cells are non-significant—VEGFA in CPTAC protein, CCND1 in all three TCGA cohorts, and MYC in TCGA STRICT, TCGA BROAD and the GEO meta-analysis. Dotted lines mark the significance thresholds: padj = 0.05 on the vertical axis and |log2 fold-change| = 1 on the horizontal axis.

**Figure 4:**
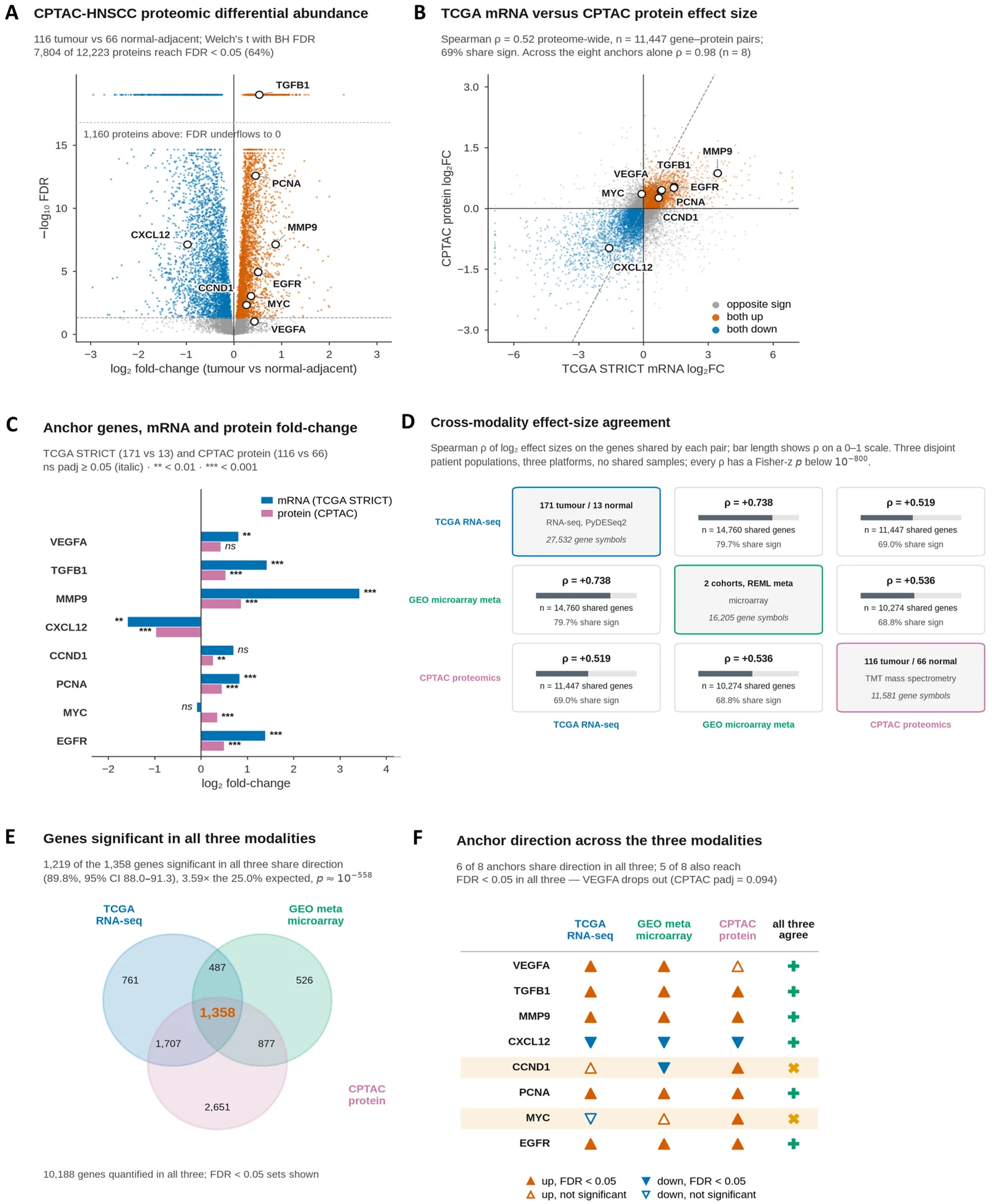
**CPTAC proteomic differential abundance, mRNA × protein concordance and three-way cross-modality replication.** (**A**) Volcano plot of CPTAC HNSCC umich proteomics (n = 116 tumours/66 normal-adjacent; 12,223 quantified proteins; 7804 significant at padj < 0.05). (**B**) mRNA × protein concordance. Two distinct quantities are labelled separately: proteome-wide Spearman ρ = 0.52 over 11,447 unique gene symbols, 69% sign-concordant, and anchor-only ρ = 0.98 (n = 8). (**C**) Anchor genes: TCGA mRNA versus CPTAC protein log_2_ fold-change with FDR tiers. (**D**) Pairwise effect-size agreement across three disjoint patient populations and three platforms: TCGA × GEO ρ = +0.738 (n = 14,760, 79.7% sign agreement), TCGA × CPTAC +0.519 (n = 11,447, 69.0%), GEO × CPTAC +0.536 (n = 10,274, 68.8%), with the co-significant subset shown alongside. (**E**) Directional concordance among genes significant in all three modalities: of 1358 such genes, 1219 (89.8%) share direction in all three against 25.0% expected by chance (2 of 8 sign patterns); the exact binomial *p* is below double-precision floating-point range and is not quoted. (**F**) Wet-lab anchor direction across the three modalities: 6 of 8 agree in all three; CCND1 and MYC are the exceptions, and the previous version named only MYC. Filled symbols denote genes concordant in direction across all three modalities and open symbols denote discordant genes; symbol size is proportional to the absolute log2 fold-change in TCGA-HNSC. Panels (**D**–**F**) present the cross-platform reproducibility check on the transcriptional programme, which is quality control and not evidence for the paracrine model (Section 3.3 and Section 3.12). The CPTAC proteoform collapse rule (12,223 rows → 11,581 symbols; 642 duplicates collapsed on maximum sample coverage, tie-broken on padj then row order) is stated in the figure footnote; none of the eight anchors is duplicated, so the rule cannot affect the anchor results. Dotted lines mark the significance thresholds: padj = 0.05 on the vertical axis and |log2 fold-change| = 1 on the horizontal axis.

Tumour-vs-normal differential expression in the STRICT oral-cavity HPV-negative TCGA-HNSC subset (n = 171 tumours/13 normals) used PyDESeq2 v0.5.4 [25,26] with design ~ condition, low-count filter row_sums ≥ 10 in ≥5 samples and default Cook’s distance refit; 27,559 protein-coding genes were tested and 10,640 reached padj < 0.05 (Figure 3A and Figure S1A). L1 is a tumour-versus-normal contrast and legitimately requires both arms; the patient-level survival models of L7 do not and are restricted to primary tumours (Section 2.2.6). GEO bulk cohorts were quantile-normalised within platform, collapsed probe-to-gene by the max-mean rule, scored with Welch’s t (auto-log_2_-transformed when median > 50) and combined by REML random-effects meta-analysis using PyMARE v0.0.10 [28]; per-anchor forests with cohort weights and Higgins I^2^ are in Figure S2A–H. CPTAC HNSCC differential abundance was computed by Welch’s t-test on log_2_-normalised umich abundance (n = 116 tumour vs. 66 normal-adjacent), yielding 7804 of 12,223 proteins at padj < 0.05 (Figure 4A and Figure S1C). For the cross-modality analysis of Section 3.3, the 12,223 CPTAC rows were collapsed to 11,581 gene symbols, the 642 duplicated symbols being resolved on maximum sample coverage (n_tumour + n_normal), tie-broken on padj then row order—the only rule among nine tested that does not select based on the statistic under test. None of the eight anchors is duplicated in any of the three tables, so the collapse rule cannot affect the anchor results.

The STRING shortest-path analysis used STRING v12.0 high-confidence physical edges mapped to gene symbols [33] via networkx v3.6.1 single_source_shortest_path_length with cutoff 4. Source set: curated GMSC secretome minus the eight wet-lab anchors. Target module: union of Hallmark EMT/Myc Targets V1/Myc Targets V2/G2-M Checkpoint/mTORC1/Hypoxia/PI3K-Akt-mTOR with KEGG focal adhesion/cell cycle/TGFβ/PI3K-Akt/MAPK/Ras/Wnt/pathways-in-cancer, restricted to genes present in the TCGA DE table and excluding the eight anchors. Empirical *p*-values were computed against 200 degree-preserving rewires (networkx.double_edge_swap, 2× edge-count swap attempts); per-pair *p* = (null_count + 1)/201. The OmniPath/SIGNOR signed-direction overlay [65,66] was applied to high-significance pairs (Supplementary Table S4).

#### 2.2.4. NicheNet Ligand Decoder (Layer L5)

**Figure 5:**
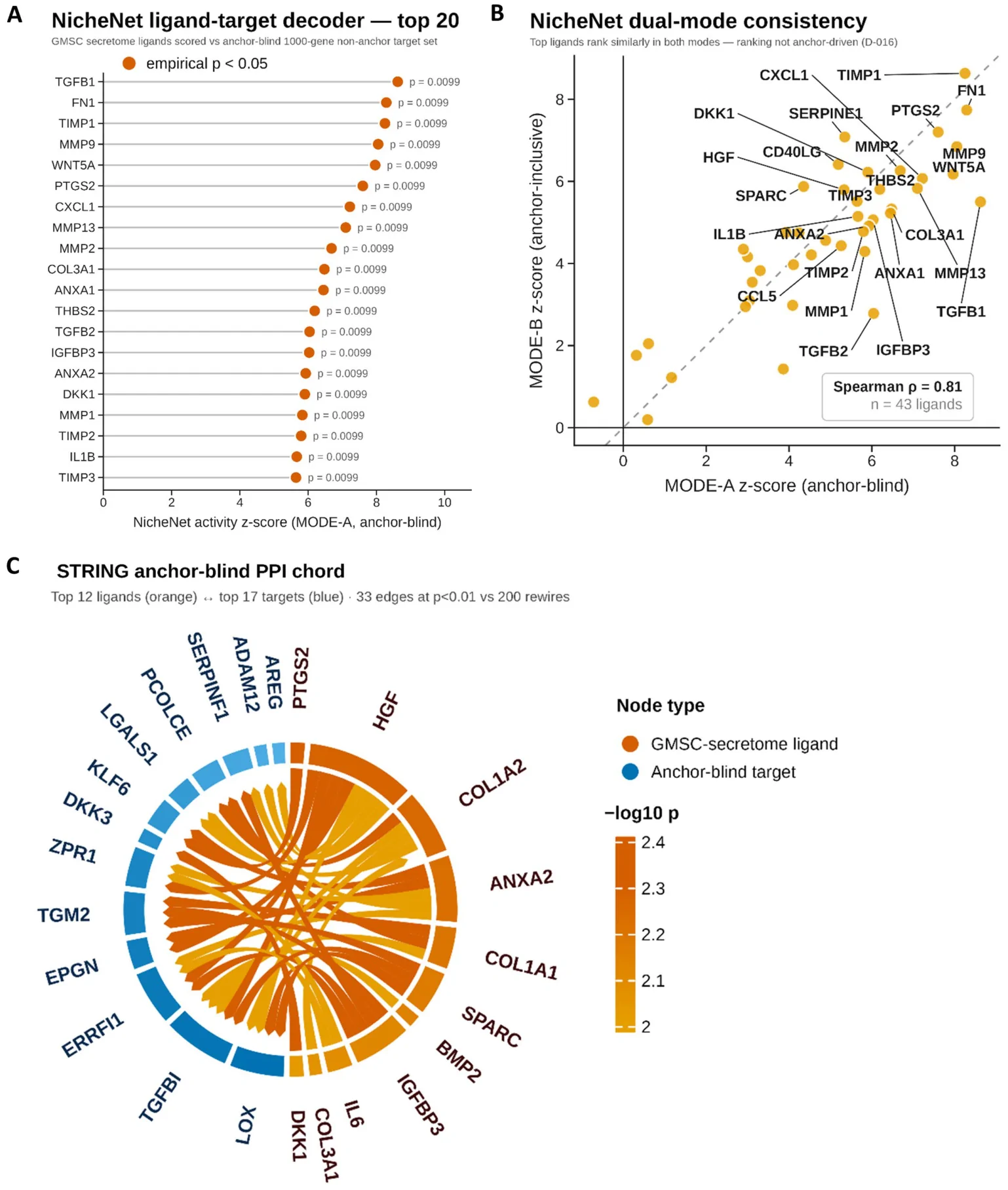
**NicheNet ligand decoding and STRING shortest-path analysis over the curated secretome.** (**A**) NicheNet activity z-scores for the top 20 GMSC secretome ligands against the anchor-excluded 1000-gene non-anchor target set (MODE-A; empirical *p* against 100 random gene-set permutations); all top 15 reach empirical *p* ≤ 0.01. (**B**) MODE-A (anchor-excluded) versus MODE-B (anchor-inclusive) z-score consistency. (**C**) STRING v12.0 shortest-path chord diagram: top 12 secretome ligands (orange arcs) → top 17 non-anchor target genes (blue arcs) at empirical *p* < 0.01 against 200 degree-preserving rewires; arc shade encodes the rewire-null −log_10_(p). These layers draw on one shared 46-ligand candidate space and are not independent priors (Section 3.4, Table 3).

Two complementary decoders were applied: (a) a ligand–receptor “fraction-in-target” decoder via liana-py v1.7.1 consensus resource [35]; (b) the NicheNet NSGA-II ligand–target regulatory potential matrix [34] (33,354 targets × 1226 ligands), scored by mean potential against the anchor-excluded 1000-gene non-anchor DE target set versus 100 random gene-set permutations. Two modes are reported: MODE-A (anchor-excluded, primary) with anchors removed from the target query, and MODE-B (anchor-inclusive sensitivity) with anchors retained (Figure 5A,B).

#### 2.2.5. Single-Cell Processing and EMT Trajectory (Layer L6)

OSCC scRNA-seq from GSE103322 [38] was re-parsed with the Puram et al. metadata schema and processed with scanpy v1.11.5 [36] (cell-level filtering n_genes = 1500–10,000; HVG selection n_top_genes = 3000, seurat-v3 flavour; PCA to 50 components; batch correction across the deposit’s 17 batch labels with harmonypy v2.0.0 [37]; Leiden clustering at resolution = 0.6 yielding 23 clusters with leidenalg v0.11.0). Full-length SMART-seq2 data do not support RNA velocity; trajectory inference therefore used PAGA on Leiden connectivity [67] plus diffusion pseudotime [68] rooted in the epithelial cluster with the highest CDH1 − VIM signal. scFates v1.2.4 [69] was retained as a scaffolding utility.

#### 2.2.6. Patient Signature, Survival and External Validation (Layer L7)

Three pre-registered signature variants were evaluated on PyDESeq2 variance-stabilising-transformed (VST) expression: (i) PRIMARY z-sum, the sum of standardised z-scores of the eight anchor genes (a consistency test, and anchor-dependent by construction); (ii) SENSITIVITY single-sample GSVA over Hallmark EMT + Myc Targets V1 + G2-M Checkpoint with anchors removed from each gene-set’s membership, via gseapy ssgsea [47]; (iii) EXPLORATORY LASSO–Cox over the same Hallmark feature pool minus the eight anchors, fit with scikit-survival v0.27.0 CoxnetSurvivalAnalysis (l1_ratio = 1.0, alpha_min_ratio = 0.01, 20 alphas), selecting the regularisation level that retained 5–15 non-zero features [39,70]. Median-stratified log-rank tests and multivariable Cox regression (adjusted for age and sex) were computed via lifelines v0.30.3 [40].

Sample-type restriction: The VST matrix is now restricted to sample_type == “Primary Tumour” before signature scoring and before the Coxnet fit, and one record per case_id is enforced. In the earlier analysis, 13 (STRICT and BROAD) and 44 (PAN-HNSC) solid-tissue normals were carried into signature scoring, model fitting and every Cox and Kaplan–Meier model; their event rate was 69.2% and 72.7% against 38.0%/36.6%/41.5% in tumours, and 13 of 13 and 43 of 44 were patient-matched to a tumour already in the cohort, so those patients and their events were double-counted. Because the LASSO was fit on the full sample set, the Coxnet coefficients were refit rather than row-filtered. The analysis cohorts are STRICT (171 primary tumours, 171 modelled), BROAD (183 primary tumours, 181 with usable overall survival) and PAN-HNSC (518 primary tumours, 512 with usable overall survival). Barcode sample-type code 01 defines the primary-tumour set. The three cohorts are strictly nested (STRICT ⊂ BROAD ⊂ PAN-HNSC, verified by file_id containment; primary-tumour Jaccard 0.934, 0.353 and 0.330), so fitting in all three is three in-sample fits on overlapping data rather than independent replication.

*Properties of the selection rule that must be stated*: On the 20-point regularisation path, the 5–15-feature rule is satisfied by exactly 1 alpha in STRICT, 2 in BROAD and 3 in PAN-HNSC; non-zero feature counts jump 3 → 9 → 19 between adjacent alphas in STRICT, so the window is straddled rather than searched and the rule behaves as a near-deterministic pick (Figure S7E). Features were not standardised before the L1 penalty (normalise = false); per-feature standard deviations span 0.30–2.96 and the selected features’ median SD sits at the 96.6th–98.4th percentile of the pool, so the penalty is not scale-neutral.

*Internal validation*: Four procedures were added, all re-executing the entire selection procedure (Coxnet path plus the 5–15-feature alpha rule) inside every training fold or resample: (i) repeated 5 × 5-fold cross-validation stratified on event status (RepeatedStratifiedKFold, seed 20260521; 25 fits per cohort, 0 fallbacks) giving a cross-validated C-index and an out-of-fold linear predictor; (ii) 500-resample bootstrap optimism correction; (iii) a 1000-iteration in-sample permutation null on the survival labels; and (iv) a 200-iteration out-of-fold permutation null, each iteration running a complete cross-validation. The submitted work specified no cross-validation scheme, so the choice of stratified repeated 5-fold is ours and is stated as such. Results are in Section 3.6 and Figure S7A–G.

External validation of the prognostic signature. In response to the review, the frozen signature was tested in an independent public cohort. GSE41613 (GPL570, Affymetrix HG-U133 Plus 2.0) [42] comprises 97 HPV-negative oral-cavity squamous cell carcinomas with per-patient follow-up; the series matrix was downloaded on 27 July 2026 (md5 ad645ea2d7fac58a141cc1e9756ca0d5). Eligibility (oral-cavity SCC with overall-survival annotation) was verified in code before any signature was scored. The endpoint is all-cause overall survival (51 events/97 patients; median follow-up 54.4 months), matching the definition used in TCGA-HNSC and not the disease-specific endpoint the original authors modelled; the deposit supports a disease-specific endpoint, which we did not run, because switching endpoints after seeing the pre-specified result would be the outcome-dependent choice this analysis exists to avoid. The complete analysis plan—cohort, preprocessing, endpoint, metrics, the variants to be run and the handling of genes absent from the platform—was fixed and written to disc before any signature score was related to outcome. Expression values were processed by the same pre-registered pipeline used for the L2 GEO cohorts: within-platform quantile normalisation across samples, probe-to-symbol assignment from the pinned GPL570 annotation (first symbol before “///”) and max-mean probe collapse, yielding 21,755 genes from 54,613 probes. The TCGA-derived LASSO–Cox coefficients were frozen: no model was refit, no feature reselected, and no penalty was returned in GSE41613; the risk score is the linear predictor Σ β~g~·x~g~ over the signature genes. All nine STRICT, all eight BROAD, and all six PAN-HNSC genes are represented on GPL570, so no gene was dropped, and the pre-declared rule for an absent gene (imputation at the cohort mean) was never invoked. Six pre-declared cells were evaluated—the full factorial of three-cohort signatures (STRICT, the pre-registered primary; PAN-HNSC and BROAD, sensitivity variants) × two standardisation conventions (within-cohort gene-wise z-score, the primary handling of the cross-platform scale difference; and no gene-wise standardisation, the convention under which the coefficients were originally fitted)—and all six are reported. Discrimination was quantified by Harrell’s C-index computed on the risk score, with a percentile 95% confidence interval from 2000 patient-level bootstrap resamples; patients were additionally dichotomised at the cohort median of the score and compared by two-sided log-rank test; and hazard ratios per standard deviation of the score were estimated by Cox regression (penalizer = 0.001) unadjusted, adjusted for age band and sex (the model used internally), and adjusted for age band, sex, tumour stage (I/II vs. III/IV) and treatment modality—the only covariates the deposit provides. The deposit reports age in bands rather than exact years and carries no grade, margin status or smoking data. No alternative cut-point, collapse rule or subgroup was examined. Because nothing was selected, fitted or tuned in this cohort, this is a single application of a frozen model with no selection step, so the permutation finding described above (which is a property of the selection procedure) does not bear on it. Analyses used scikit-survival v0.27.0 [39] and lifelines v0.30.3 [40] with seed 20260521. Results are in Section 3.6 and Figure S7H.

#### 2.2.7. Functional Activity Inference and Anchor-Excluded GSEA (Layers L8, L9, L13)

Pathway activities were inferred with PROGENy [43] via decoupler-py v2.1.6 dc.mt.mlm [45] and transcription-factor activities with CollecTRI [44] via dc.mt.ulm, both on the 171 STRICT primary tumours (Figure 8A,B). Score matrices were stratified by the z-sum signature and tested by Wilcoxon rank-sum with Benjamini–Hochberg correction. Because PROGENy and CollecTRI were re-fetched from OmniPath, we ran a drift control: re-running the analysis with the 25 July 2026 snapshot exactly reproduced the deposited outputs (max |Δ| = 0.0 across 764 L8 and 13 L13 rows), so every change reported in Section 3.7 is the tumour-only correction and not network drift.

Immune-compartment activity (L13) was inferred with dc.mt.ulm against a curated 17-cell-type marker panel consolidated from CellMarker 2.0 and classical immunology references, of which 13 were scored: cDC1, cDC2, pDC and Pericyte were dropped by decoupler’s min_npruning (four markers present each). This applies to the earlier analysis as well as the corrected one. A marker-based ULM approach was preferred over reference deconvolution such as CIBERSORTx [71] because it requires no external signature matrix and is internally consistent with L8; it scores activity, not abundance. Differential immune-compartment activity between the z-sum strata was tested by Wilcoxon rank-sum with Benjamini–Hochberg correction on the same 171 primary tumours (Figure 8D).

Anchor-excluded GSEA: pre-ranked GSEA [72] was performed via gseapy.prerank against the Hallmark, KEGG, Reactome and GO-BP Enrichr libraries [47,48,49,50,51,64]. Genes were ranked by signed −log_10_(*p*-value), with the eight anchors removed both from the ranking statistic and from each gene set’s membership; 1000 permutations, min_size = 5, max_size = 1000. Fifteen gene sets that dropped below 5 members after anchor exclusion were excluded and are enumerated in Figure S6A,B and Supplementary Table S8.

#### 2.2.8. Cell–Cell Communication and Matrix Decomposition (Layers L10, L10b)

Cell–cell ligand–receptor signalling was inferred using LIANA v1.7.1 [35] consensus aggregation (liana.mt.rank_aggregate) across CellPhoneDB [52], NATMI [53] and Connectome [54], aggregated by geometric-mean specificity rank. Inputs: min_cells = 10 per group, expr_prop = 0.1, n_perms = 1000. Outputs were filtered to fibroblast (Puram’s CAF compartment) → malignant pairs (Figure 9A; full list in Figure S3A).

Context-aware tensor decomposition was attempted via cell2cell [55] but could not be run because of an API incompatibility recorded in our decision log, and a truncated singular value decomposition (SVD) of the 12 patients × 1476 LR-pair score matrix was substituted; five components were retained (Figure 9B,C; loading heatmap in Figure S3B). No tensor was constructed. We also note that the score matrix was not mean-centred, so component 1 carries 91.1% of the retained sum of squares with all 12 patient loadings and all 1476 LR loadings of the same sign: it is an overall-magnitude (scale) axis, not a latent communication pattern. Reference [55] is a review of cell–cell communication inference and is cited as the source of the approach, not as a software citation.

#### 2.2.9. Direction-Resolved Drug Repurposing (Layer L12)

Top 100 up- and top 100 down-regulated genes from the TCGA-HNSC oral-cavity HPV-negative DE contrast (anchors excluded) were queried via gseapy Enrichr [46] against three LINCS L1000 libraries: LINCS_L1000_Chem_Pert_Consensus_Sigs, LINCS_L1000_Chem_Pert_up and LINCS_L1000_Chem_Pert_down [56,57]. The same procedure was applied to the companion apoptosis × ROS contrast [13] using that paper’s L1 DE output (anchor set BAX, BCL2, CASP3, CASP9, NOX1, GPX1; anchors excluded from both). A regex-based parser extracted compound names from Enrichr term strings. Per-compound connectivity was aggregated as the maximum −log_10_(adjusted *p*) across the three libraries, and a score S(drug) = z(primary-axis connectivity) − λ × z(companion-axis connectivity) was computed for λ ∈ {0.5, 1.0, 2.0}.

Three properties of this construction are load-bearing for its interpretation.

1. The aggregation is direction-agnostic. Pooling the tumour-up and tumour-down query results, and within the consensus library the <drug> Up and <drug> Down terms, means a compound whose LINCS-up signature matches the tumour-up list scores identically to one whose LINCS-down signature does. In a direction-resolved re-analysis of the same cached responses, of the 74 genuine compounds with any significant primary-axis term, 35 (47.3%) were reversal-only and 39 (52.7%) mimicry-only; within the top 30 of the reported ranking, 15 were reversal-only, 13 mimicry-only and 2 significant in both. Direction is therefore resolved and reported per compound throughout Section 3.10 and Figure 10.
2. Twenty-five plate identifiers were admitted as compounds in error. ljp005– ljp009and cpc001– cpc020 are LINCS plate barcodes, not compounds: the parser takes the first alphanumeric token of the ‘<PLATE> <CELL> <TIME>-<perturbagen>-<dose>’ grammar, collapsing 36,075 rows carrying 2900 genuine perturbagens into 52 tokens, 25 of them barcodes (ljp006alone absorbs 6955 rows against a median of 4 per token). These are now flagged; the compound universe is approximately 5297 LINCS consensus perturbagens.
3. The companion axis carries no signal. No compound reaches padj < 0.05 anywhere on the companion axis (minimum padj 0.2552), and z (companion) takes only 15 distinct values across 5332 tokens, so the subtraction operates on a null background. This is a property of our re-derivation from the companion’s differential-expression table under this paper’s query construction, and is not a re-run of the procedure by which the companion obtained its own drug candidates (Section 4.5). No quadrant scheme is applied, and Figure 10 does not draw one.

Top compounds were cross-annotated with DGIdb v5 [58] via its GraphQL API. DGIdb’s approved flag is a general regulatory-approval flag and is not specific to any one national regulator. DGIdb v5 returned no ATC code for any compound (0/30) and no approval year (0/30); drug class was returned for 1 of 30. Only the top 30 compounds were queried, so approval status is unknown for 5302 of the 5332 tokens. Where an ATC code is quoted in this paper, it is sourced from the WHO ATC index, not from DGIdb.

#### 2.2.10. DepMap CRISPR Essentiality Cross-Reference (Layer L14)

L12-predicted drug targets and the eight wet-lab anchors were cross-referenced with DepMap Public 24Q4 Chronos gene-effect scores [59] across 53 oral-cavity squamous cell carcinoma lines (OncotreeLineage == ‘Head and Neck’AND OncotreePrimaryDisease == ‘Head and Neck Squamous Cell Carcinoma’AND OncotreeSubtype == ‘Oral-Cavity Squamous Cell Carcinoma’; 62 models, 53 with Chronos data) as the primary stratum, with the 77 head-and-neck-lineage lines (95 models, 77 with data) as a sensitivity stratum. Essentiality was defined as median gene effect < −0.5 and strong essentiality at <−1.0; the per-gene fraction of lines essential is reported (Figure 11A and Figure S5A,B). To distinguish lineage-relevant from generic proliferative dependency, each gene was additionally annotated with its DepMap 24Q4 Common Essential status, read directly from CRISPRInferredCommonEssentials.csv as shipped with the release, and with a lineage-selectivity difference defined as the oral-cavity median Chronos score minus the median across all 1178 lines screened in the release; the fraction of those 1178 lines essential at the same −0.5 threshold is reported alongside (Table 4). For reference, we also computed normLRT, the statistic underlying DepMap’s Strongly Selective designation, as 2 × (log-likelihood of a skew-t fit − log-likelihood of a normal fit) to each gene’s dependency profile across all screened lines, against the conventional threshold of 100. This is our own reimplementation and not the portal value: DepMap fits the Azzalini skew-t whereas we fit the Jones–Faddy skew-t implemented in SciPy 1.17.1, so the ordering is preserved, but the decimals are not expected to match. The Common Essential flag is from the release; the normLRT column is ours, and the selectivity difference, which requires no distributional fit, is the quantity on which the lineage argument in Section 3.11 rests. Panel membership is derived from Model.csv metadata only. The panel analysed was assembled by unioning a hard-coded ModelID list with the lineage filter and consequently contained 11 non-head-and-neck lines; this is corrected here and disclosed in Section 3.11 and Section 4.7. The metric is Chronos, which DepMap 24Q4 ships; the label “CERES” used in the submitted Supplementary Figure S5 was wrong.

#### 2.2.11. Spatial Transcriptomic LR Examination (Layer L15)

GSE300414 [60] was processed by parsing 293 DCC files via a custom Python routine (RTS_ID → gene-symbol map from the Hs_R_NGS_WTA_v1.0.pkc probe-kit file). AOI assignment used the sample-title convention hnscc_XX.ROI_YYY.<Compartment>.<budding|non_budding>. The analysis differs from the earlier one in five respects, each of which changes the result.

- Normalisation: Counts are Q3-normalised—the 75th percentile computed on the 8309 targets above the per-AOI negative-probe limit of quantification in ≥5% of AOIs, then applied to all 18,676 targets—followed by log2(x + 1). The submitted analysis correlated raw, unnormalised DCC counts. AOI library size spans 0 to 5.01 M, and stromal and tumour depth co-vary at ρ = 0.921 across the 24 patients, so on raw counts almost any two expressed genes correlate.
- AOI quality control: GeomxTools segment-QC defaults were applied (raw reads ≥ 1000; ≥80% trimmed and stitched; ≥75% aligned; ≥50% sequencing saturation; >0 endogenous counts). Four no-tissue negative controls and 13 tissue AOIs were dropped, all 13 for sequencing saturation below 50%, leaving 276 AOIs (126 stromal, 125 tumour, 25 immune). Nuclei count and surface area could not be checked because the GeoMx annotation worksheet was not deposited, so segment QC is partial.
- Heteromeric receptors: Complexes are resolved as the minimum across subunits, recovering 30 support-joined pairs that the earlier rule dropped.
- Test set: All 153 GMSC-secretome-filtered fibroblast → malignant pairs are tested. The submitted analysis tested 30 of the smallest (40) cut of the LIANA ranking. The 153-pair set is the pre-specified universe; the N = 40 cut was a post hoc, un-pre-registered analyst choice made before any correlation was computed, so the selection is outcome-independent but was not pre-registered. We state this rather than defend it.
- Null: The permutation null specified in our layer design is run as follows: 10,000 draws of non-cognate ligand × receptor pairs (249 ligands × 245 receptors, 902 cognate combinations excluded), giving a calibrated empirical p per pair alongside the parametric Spearman p. Benjamini–Hochberg correction is applied across all 153 pairs.

#### 2.2.12. Anchor Exclusion as a Bias-Control Procedure

*Method*: The eight wet-lab anchors are excluded from the STRING source and target seed sets (L4); the NicheNet target query in MODE-A (L5); the LASSO–Cox feature pool (L7 method iii); the GSEA ranking statistic and each library’s gene-set membership (L9); and the LINCS query gene lists (L12). Quantitatively, the operation reduces the STRING source set from 46 to 42 ligands, the LASSO–Cox feature pool from 568 to 562 genes, and rewrites 989 of 7882 gene sets across the four GSEA libraries; 15 sets fall below the 5-member floor and are dropped (Supplementary Table S8). An automated audit script asserts compliance after each such layer runs, and the audit log is deposited with the code so that the assertion count can be checked directly rather than quoted.

What this is. It is a bias-control procedure, not a novel statistical methodology, and we withdraw the framing that presented it as one. The problem it addresses—that selecting features using the same information that is later used to test them produces optimistically biassed statistics—is long established in the circular-analysis and selection-bias literature [19,20,21,22], and the remedy of excluding the selection-defining variables from the analysis set is the standard one. Five of the thirteen layers apply the exclusion. Table 2 states which they are and what it does in each.

Limitations of the procedure: three, all of which weaken it.

1. It removes genes, not pathways. In total, 10.0% (56 of 562) of the retained LASSO–Cox feature pool are STRING high-confidence direct interaction partners of an excluded anchor. Two of them are selected features: AREG, an EGFR ligand, in PAN-HNSC, and MMP3, an MMP9 paralogue, in STRICT and BROAD. A signature built without EGFR that selects an EGFR ligand is not blind to EGFR biology.
2. It does not protect the layers that do not apply it. L1, L2, L3, L6, L8, L10, L13 and L14 use the anchors as read-outs or not at all, so the exclusion is silent there; where the anchors are reported as results (Section 3.2, Section 3.3 and Section 3.12), the analysis is anchor-dependent by design and is labelled as a consistency check rather than as independent discovery.
3. It cannot address selection at the level of the candidate space. The 46-ligand secretome list and the Hallmark feature pool were chosen by us from the literature that includes MSC-paracrine studies. Excluding eight genes from a list assembled under that prior does not make the list prior-free.

#### 2.2.13. Reproducibility, Environments and Code Release

All Python analyses were performed in Python 3.11.9 within uv-managed virtual environments: hybrid-main (general bioinformatics, survival, statistics), hybrid-sc (single-cell, cell–cell communication, trajectory) and hybrid-cptac (PNNL cptac 1.5.14). The hybrid-foundation environment has been removed along with the withdrawn L11 layer; the torch and transformers version discrepancy identified in review therefore no longer bears on any reported result, and for the record the locked versions were torch 2.12.0 and transformers 5.9.0 (the manuscript printed 4.57.6, a major-version error, and 2.10.0 for torch in one place). Two further version corrections: matplotlib is 3.11.0rc2, a pre-release, in all locks (the manuscript printed 3.11.0), and pandas is 2.3.3 in hybrid-mainbut 3.0.3 in hybrid-cptac, so the L3 proteomic analysis ran on pandas 3.x. Python lock files are deposited. No renv. lock exists: the repository contained only a renv.lock.skeleton declaring R 4.4.0 against the R 4.3.3 actually used, so the claim “lock-files are saved” was true of Python and false of R; a genuine renvsnapshot is generated and deposited, the skeleton deleted, and the unused envs/hybrid-spatial/directory removed. The core scientific stack is numpy [73], scipy [74], pandas [75] and matplotlib [76] in Python, and R 4.3.3 with ggplot2 [77], ComplexHeatmap [78], circlize [79] and patchwork [80] for figure rendering. The exact version of every package in every environment is fixed by the deposited uvlock-files and the renvsnapshot, which are the authoritative inventory; we have deliberately not duplicated them as a printed table, because a printed table can drift from the lock-file and the lock-file cannot drift from the environment. The global random seed is 20260521, applied to numpy, scanpy, gseapy and LIANA.

## 3. Results

### 3.1. GMSC Characterisation and the Wet-Lab Perturbation: A Transcriptional Shift Without a Functional One (Figure 1 and Figure 2)

Flow cytometry confirmed the tested surface phenotype (CD90 99.1%, CD105 99.9%; CD45 1.50%, HLA-DR 5.02%; a second panel on the same cell system is reported in the companion study [13] and is set out in Section 2.1.3) and characteristic spindle-shaped morphology (Figure 1), consistent with an MSC phenotype; the criteria not assessed, and the values that place the cells outside the ISCT minimal criteria, are set out in Section 2.1.3. Primary OSCC cells were treated for 24 h with GMSC-CM, or maintained under indirect Transwell co-culture, and assayed for metabolic activity (MTT), migration (scratch wound) and the eight-gene RT-qPCR panel (Figure 2).

The transcriptional read-out: All eight transcripts (VEGFA, TGFB1, MMP9, CXCL12, CCND1, PCNA, MYC and EGFR) were raised above the OSCC control in all three treatment arms, and 16 of the 24 gene × arm comparisons reached significance (9 at *p* < 0.01, 7 at *p* < 0.05; 8 not significant) (Figure 2C). Every proliferation-specific transcript in the panel (CCND1, PCNA, MYC) was significantly raised in at least one arm. This is the paper’s strongest wet-lab evidence, and it establishes a coordinated pro-tumour transcriptional shift under GMSC paracrine exposure, opposite in direction to the apoptotic/ROS-suppressive shift reported in the companion study on the same cell systems [13]. We write consistent with, and not establish, for a reason intrinsic to the measurement. The panel is normalised to a single reference gene whose stability under GMSC-CM exposure was not assessed, and target-versus-reference amplification efficiencies were never determined (Section Constraints on Experimental Re-Validation); because all eight targets move in the same direction in all three arms, a global shift in the reference gene remains a coherent alternative explanation for part of that uniform direction. It does not account for the relative ordering across the eight genes, but on its own the panel indicates the shift rather than establishing it.

The functional read-outs did not follow, and we report that plainly. Metabolic activity did not differ significantly between arms: the MTT comparison spanning all seven arms is annotated ns, and the OSCC control absorbance exceeds that of GMSC-CM 5%, 10%, 25% and the Transwell arm (Figure 2A). Wound closure was significantly reduced under GMSC-CM 50% and GMSC-CM 100% relative to OSCC control (both *p* < 0.01), while the Transwell arm did not differ from control (Figure 2B). The pro-tumour claim in this paper therefore rests on the transcript panel, which supports it, and not on the functional assays, which do not.

*A candidate explanation, explicitly untested.* GMSC-CM is harvested into serum-reduced DMEM (Section 2.1.4), so the two conditioned-medium arms are serum-depleted relative to a 10% FBS control—more so if the conditioning followed the serum-free, 48 h protocol described in the companion study [13] (Section 2.1.4), whereas the Transwell arm (the only serum-matched treatment) is null in both functional assays. Twenty-four-hour scratch closure is strongly serum-dependent, and this asymmetry is consistent with the observed pattern: reduced closure exactly where serum is reduced, no change where it is not. We state clearly that this is an inference and not a result. The experiment that would test it is a serum-matched conditioned-medium plate, which we cannot now run (Section Constraints on Experimental Re-Validation). Until it is run, the correct reading of Figure 2 is that GMSC paracrine exposure raises a panel of pro-tumour transcripts in primary OSCC cells, consistent with induction of a pro-tumour transcriptional programme, while the functional consequences under these particular culture conditions remain undemonstrated.

The same asymmetry bears on the transcript panel, and we state where it does, and does not, reach. The two conditioned-medium arms are serum-depleted relative to the 10% FBS control; the Transwell arm is not. All eight transcripts are raised above control in all three arms, including the serum-matched one, which is the main reason we do not attribute the transcriptional shift to serum depletion. We stop short of a stronger statement for two reasons. The arm-by-arm distribution of the 16 significant comparisons is not tabulated here: the underlying Cq values are not recoverable (Section Constraints on Experimental Re-Validation), and we have not re-derived per-arm counts from the plotted figure. And a single serum-matched arm at *n* = 3 cannot exclude a contribution from serum depletion in the conditioned-medium arms. The experiment that would settle it is the serum-matched conditioned-medium plate named in Section 4.8, and until it is run, the correct reading is that the transcriptional programme is present in a serum-matched treatment and is not explained by serum depletion alone.

### 3.2. Cross-Cohort Transcriptomic Prioritisation in TCGA-HNSC and a Two-Cohort GEO Meta-Analysis (Figure 3)

PyDESeq2 differential expression on the STRICT oral-cavity HPV-negative TCGA-HNSC subset (n = 171 tumours/13 normals; cohort definitions in Figure S1A) identified 10,640 genes at padj < 0.05 out of 27,559 protein-coding genes (Figure 3A). Six of the eight anchors reached padj < 0.05 in the predicted direction: MMP9 log_2_FC = +3.43, padj = 7.75 × 10^−13^; TGFB1 +1.43, 3.76 × 10^−14^; EGFR +1.40, 8.64 × 10^−4^; PCNA +0.84, 2.01 × 10^−5^; VEGFA +0.81, 5.81 × 10^−3^; CXCL12 −1.59, 2.29 × 10^−3^. CCND1 (+0.71, padj = 0.082) was not significant, and MYC was non-significant (−0.09, padj = 0.78). The submitted text described “seven of the eight” as significantly dysregulated while listing CCND1’s padj as 0.082 in the same sentence; six is the correct count.

The REML random-effects meta-analysis across GSE30784 + GSE37991 returned 16,205 pooled genes (5015 at meta_padj < 0.10) and recovered the same anchor-direction profile. Across the 14,760 genes shared by the TCGA STRICT and GEO meta tables, the two sets of log_2_ fold-changes agree at Spearman ρ = +0.738 with 79.7% sign agreement, rising to ρ = +0.896 (Pearson r = 0.84) among the 2337 genes co-significant at padj < 0.05 in both, which is the subset Figure 3B highlights. The agreement does not depend on where the co-significance threshold is set: relaxing the GEO criterion to meta_padj < 0.10 admits 2665 genes and returns ρ = +0.893 and Pearson r = 0.84. Per-anchor REML forests with cohort weights and Higgins I^2^ are in Figure S2A–H. Figure 3C now prints the log_2_ fold-change and its BH-FDR tier in every cell of the five-modality anchor grid, with “ns” shown in all seven non-significant cells: VEGFA in CPTAC protein, CCND1 in all three TCGA cohorts, and MYC in TCGA STRICT, TCGA BROAD and the GEO meta-analysis. The missing annotation on the VEGFA CPTAC cell identified in the review reflected genuine non-significance (+0.432, padj = 0.094), not an omission.

### 3.3. Protein-Level Integration and Three-Way Cross-Platform Reproducibility Check on the Transcriptional Programme (Figure 4)

CPTAC HNSCC umich proteomics (n = 116 tumours/66 normal-adjacent) yielded 7804 significant proteins at padj < 0.05 of 12,223 quantified (Figure 4A). Two distinct concordance statistics must be separated, because the earlier text merged them. Proteome-wide, mRNA and protein effect sizes agree at Spearman ρ = 0.52 (0.519) over 11,447 unique gene symbols, *p* < 10^−300^, with 69% sign concordance (Figure 4B); the value ρ ≈ 0.4 printed in the earlier text and Figure 4 caption was wrong, and the figure’s own annotation was right. Restricted to the eight anchors, concordance is ρ = 0.98 (n = 8)—a different and much smaller quantity that should not be quoted as if it were the proteome-wide figure (Figure 4B, lower annotation).

*Two anchors, not one, are cross-modality discordant.* The submitted claim that MYC was “the only anchor showing a frank mRNA → protein discordance” is incorrect on both counts. CCND1 is significantly DOWN in the GEO meta-analysis (−0.313, padj = 2.11 × 10^−3^) while being UP but non-significant in TCGA (+0.706) and UP and significant in CPTAC protein (+0.264, padj = 4.68 × 10^−3^)—a significant contradiction between two independent patient cohorts, and a stronger form of discordance than the one we described. MYC’s mRNA measurements are non-significant in both TCGA (padj = 0.78) and GEO (padj = 0.50) and significant only in CPTAC (+0.36), so there is no established mRNA direction from which the protein could be discordant. Post-transcriptional regulation remains one interpretation of MYC’s profile, but it is an interpretation among alternatives: between-cohort heterogeneity for MYC is extreme (I^2^ = 99%, Figure S2G), so platform and cohort effects are at least as plausible, and we no longer assert the post-transcriptional reading (Figure 4C,F).

The three modalities can also be compared directly, and the comparison is reported here as a cross-platform reproducibility and quality-control check on the transcriptional programme rather than as a line of evidence for the paracrine model (Section 3.12). It does not fill the evidential slot left by the prior-knowledge convergence test (Section 3.4 and Section 4.2). It rests on the L1, L2, and L3 outputs. Across the three modalities, effect sizes agree pairwise at Spearman ρ = +0.738 (TCGA × GEO meta, n = 14,760 shared genes, 79.7% sign agreement), +0.519 (TCGA × CPTAC, n = 11,447, 69.0%) and +0.536 (GEO × CPTAC, n = 10,274, 68.8%), rising to +0.896, +0.765 and +0.750 among co-significant genes (Figure 4D). Of the 1358 genes reaching FDR < 0.05 in all three modalities, 1219 (89.8%, 95% Clopper–Pearson CI 88.0–91.3%) share the same direction in all three (636 up in all three, 583 down in all three) against 25.0% expected by chance (2 of 8 sign patterns), a 3.59-fold enrichment; the exact binomial *p* is below double-precision floating point range and is not quoted (Figure 4E). The figure is robust to the proteoform-collapse rule: across nine alternative rules, the concordance ranges only 88.7–89.8%. At the anchor level, 6 of 8 anchors agree in direction across all three modalities (VEGFA, TGFB1, MMP9, CXCL12, PCNA, EGFR) and 5 of 8 also reach FDR < 0.05 in all three, VEGFA dropping out at CPTAC padj = 0.094; the two exceptions are CCND1 and MYC (Figure 4F).

Three scope statements belong with this result. It is effect-size agreement in the tumour-versus-normal contrast, across three disjoint patient populations and three measurement platforms with no shared samples. It is not prognostic validation—a programme can replicate perfectly in a tumour-versus-normal contrast and carry no prognostic information, which is what Section 3.6 shows—and it is not mechanistic convergence. And the CPTAC arm is HNSCC-wide rather than oral-cavity restricted, the same anatomic scope caveat that applies to the PAN-HNSC survival cohort (Section 3.6 and Section 4.7).

Two qualifications apply to this analysis. First, its status: it is a post hoc analysis, specified after the prior-knowledge convergence test it replaces had been performed. We report it because withdrawing a claim and leaving the section empty would conceal a real feature of the data—not because its *p*-value should be read as though it had been pre-registered. Second, its null: the 25.0% chance expectation treats the three modalities as independent draws on sign, and they are not independent. All three are tumour-versus-normal contrasts on the same disease, so a gene with a large true effect will tend to share direction across them for reasons that have nothing to do with GMSC paracrine signalling. What this analysis therefore establishes is the cross-platform reproducibility of direction in three disjoint patient populations measured on three technologies (a real and, we think, useful property of the programme) and not that the three modalities constitute three independent tests of a hypothesis. It is accordingly presented as quality control on the transcriptional programme and is deliberately excluded from the lines of evidence for the paracrine argument (Section 3.12); we do not use it as an independent test anywhere in the paper, and it does not restore the withdrawn convergence claim.

### 3.4. Ligand Nomination over a Shared Candidate Space: NicheNet and STRING (Figure 5)

The NicheNet NSGA-II ligand–target regulatory potential matrix [34] was queried with the 46 curated GMSC secretome ligands against the 1000-gene anchor-excluded TCGA DE non-anchor target set. Forty-three secretome ligands had coverage in the prior, of which all top 15 (TGFB1, FN1, TIMP1, MMP9, WNT5A, PTGS2, CXCL1, MMP13, MMP2, COL3A1, ANXA1, THBS2, TGFB2, IGFBP3, ANXA2) reached empirical *p* ≤ 0.01 against 100 random gene-set permutations (Figure 5A). MODE-A (anchor-excluded) and MODE-B (anchor-inclusive) z-scores were tightly correlated (Figure 5B), confirming that the anchor genes themselves do not drive the ligand ranking.

The STRING shortest-path analysis, with the eight anchors excluded from both source and target seed sets, identified 1236 source–target pairs at empirical *p* < 0.05 against 200 degree-preserving rewires, from 106,724 candidate pairs evaluated. Top source ligands by number of significant downstream paths were HGF (84 paths), PTGS2 (76), FN1 (66), EGF (53), COL1A2 (49), IGFBP3 (48), IGF1 (42), COL1A1 (42), SPARC (42), WNT5A (42), PDGFB (41), IL6 (40), BMP2 (40), ANXA2 (38) and FGF2 (38). Top downstream targets, independent of the wet-lab anchors, were the pro-invasive ECM-remodelling cluster LOX, TGFBI, TGM2, IBSP, LGALS1, FN1, MMP7, FMOD, FBLN5 and MMP14. Figure 5C visualises the top-12 ligand × top-17 target edges.

We withdraw the claim that these constitute independent converging priors. Two mechanistically distinct rationales (regulatory-potential ranking and PPI shortest-path topology) applied to the same pre-registered 46-ligand candidate space select overlapping subsets of it, and the overlap is close to what the candidate space alone predicts. STRING nominates 40 of 46 ligands, which is 40 of 42 (95.2%) of those actually available to it once the four anchor-collision ligands (TGFB1, VEGFA, MMP9, CXCL12) are removed by construction; NicheNet nominates 38 of 46 and LIANA (Section 3.8) 27 of 46; the union of all layers is 45 of 46—only IL8/CXCL8 is nominated by nothing. The observed three-way intersection of 22 ligands does not exceed a within-universe permutation null (19.38 ± 1.61 over 20,000 draws; empirical *p* = 0.091), and the layers do not agree on ranking (Kendall’s W = 0.312, χ^2^ = 19.68, df = 21, *p* = 0.541). The correct statement is that three distinct rationales cover most of a curated list, which is a statement about the list.

### 3.5. Single-Cell Map of Cell States and an EMT Pseudotime Trajectory (Figure 6)

**Figure 6:**
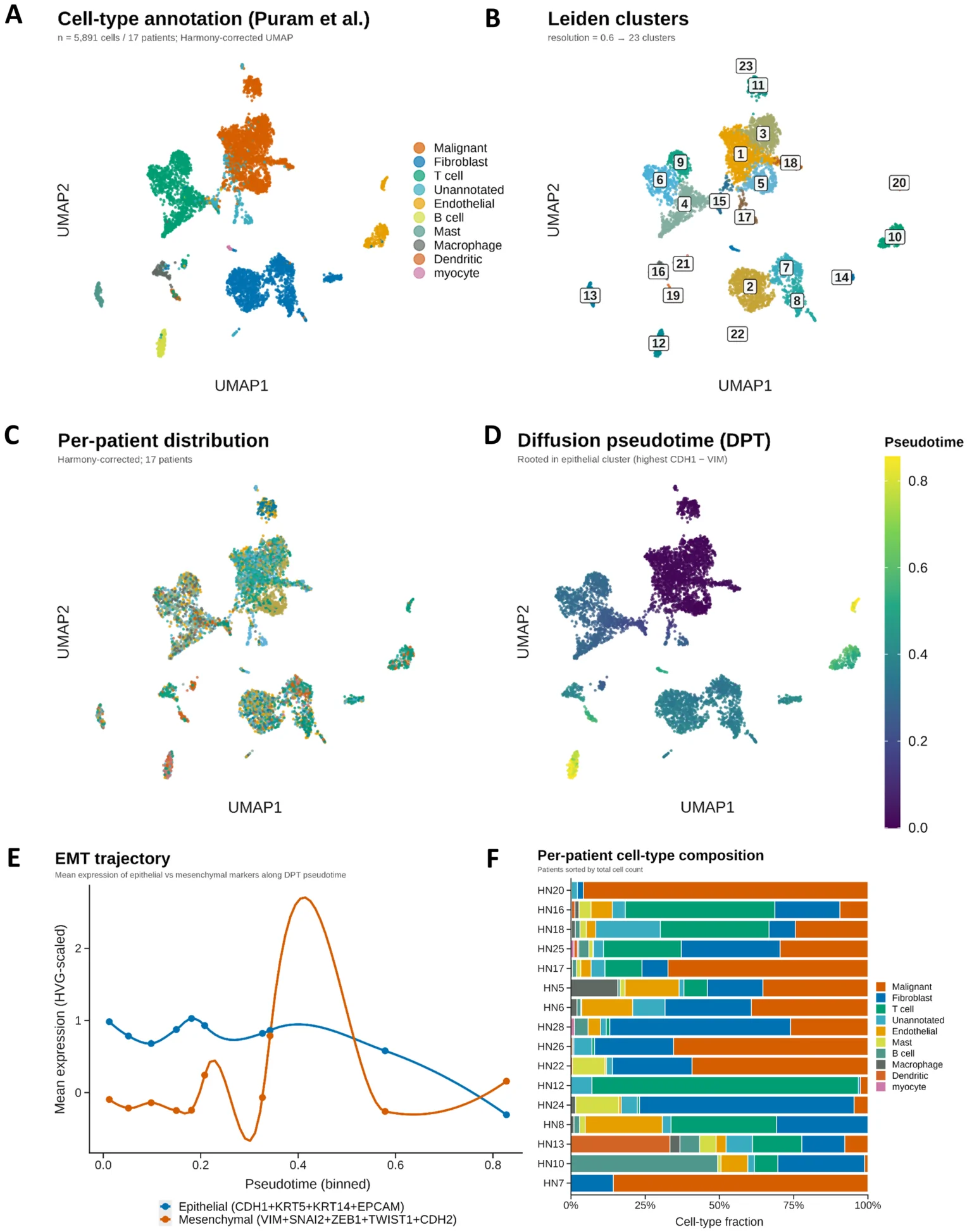
**Single-cell RNA-seq cell-state map and EMT pseudotime trajectory in HNSCC.** (**A**) UMAP of GSE103322 (5891 cells after quality filtering, 17 batch labels; Section 2.2.2) coloured by Puram et al. [38] cell-type annotation (2205 malignant; 1440 fibroblast; 1237 T cell; 260 endothelial; and other annotated types). (**B**) Leiden clustering at resolution 0.6, 23 clusters. Numbers in (**B**) are Leiden cluster indices and carry no ordinal meaning. (**C**) Per-batch distribution after Harmony batch correction. (**D**) Diffusion pseudotime rooted in the epithelial cluster with the highest CDH1 − VIM signal. (**E**) Pseudotime-binned mean expression of epithelial (CDH1, KRT5, KRT14, EPCAM) versus mesenchymal (VIM, SNAI2, ZEB1, TWIST1, CDH2) markers in the malignant compartment. (**F**) Per-batch cell-type composition across the 17 batch labels.

OSCC scRNA-seq from GSE103322 (5891 cells after quality filtering, across the deposit’s 17 batch labels; Section 2.2.2) was processed and annotated using Puram et al.’s classified-as-cancer flag and non-cancer cell-type labels: 2205 malignant cells, 1440 fibroblasts (CAF compartment), 1237 T cells, 260 endothelial, 138 B cells, 120 mast, 98 macrophage, 51 dendritic and 19 myocytes (Figure 6A). Harmony batch-corrected UMAP showed clean separation by cell type (Figure 6B) and good per-batch mixing (Figure 6C). Diffusion pseudotime rooted in the most-epithelial malignant cluster (Figure 6D) ordered an EMT trajectory along which epithelial markers (CDH1, KRT5, KRT14, EPCAM) progressively declined while mesenchymal markers (VIM, SNAI2, ZEB1, TWIST1, CDH2) increased (Figure 6E). The per-batch cell-type composition across the 17 batch labels (Figure 6F) confirmed cross-batch consistency of the strata used for the ligand–receptor inference of Section 3.8. This atlas contains no GMSCs; the fibroblast compartment is a CAF population and is used as a tissue-resident stromal proxy, not as a model of gingival MSCs.

### 3.6. Patient-Cohort Survival: Apparent Stratification Does Not Survive Internal Validation in the Oral-Cavity Cohorts, and the Pre-Registered Signature Does Not Externally Validate (Figure 7 and Figure S7)

**Figure 7:**
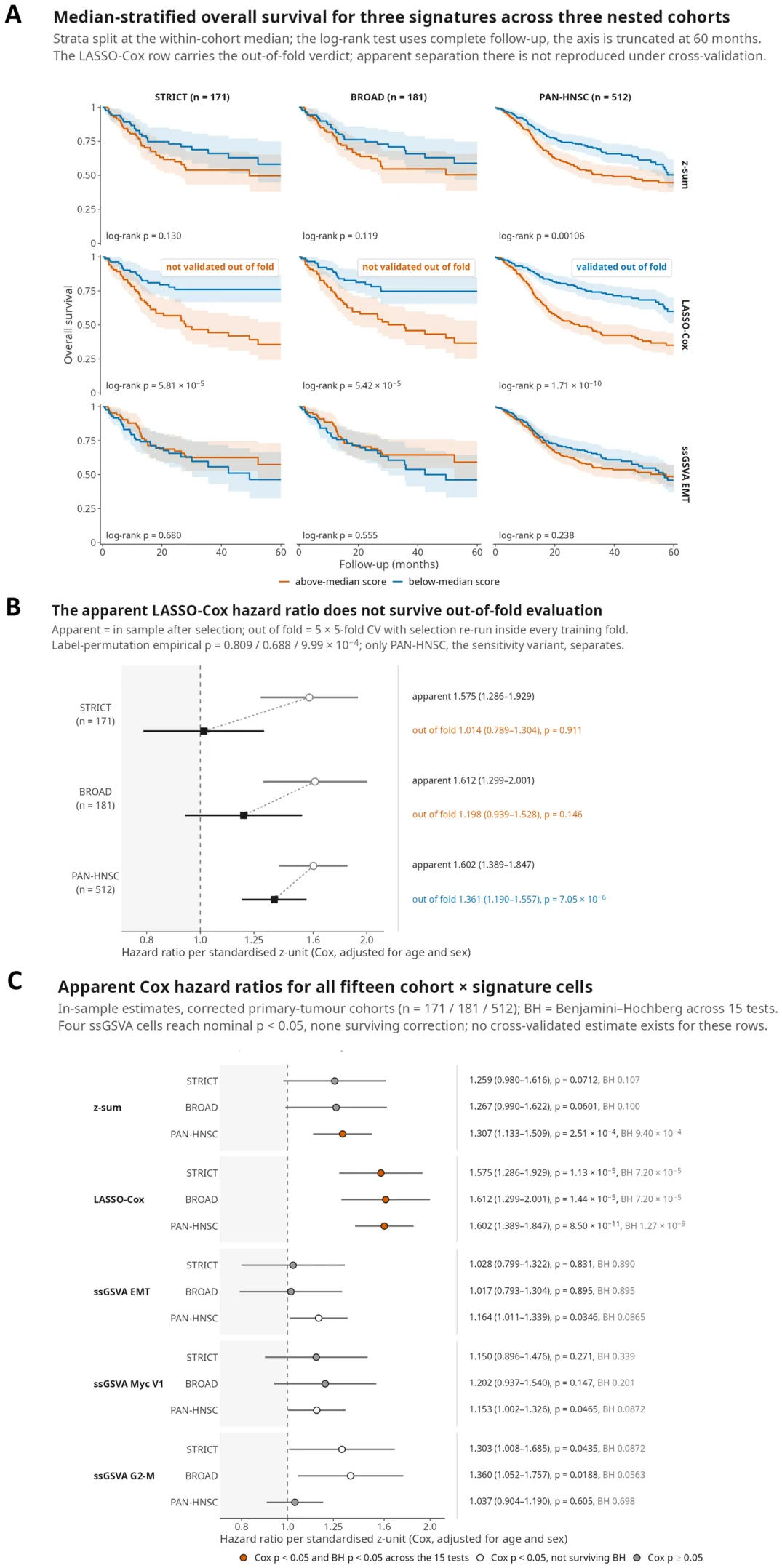
**Patient-cohort survival across three signature methods and three nested cohort variants, primary tumours only.** (**A**) Nine-panel Kaplan–Meier grid: rows are signature method (z-sum/LASSO–Cox/ssGSVA EMT), columns are cohort variant (STRICT n = 171; BROAD n = 181 modelled out of 183 primary tumours; PAN-HNSC n = 512 modelled out of 518). Strata split at the within-cohort median; log-rank *p* uses complete follow-up, and the axis is truncated at 60 months. The LASSO–Cox panels carry the internal-validation verdict established in panel B. (**B**) Apparent versus out-of-fold hazard ratios. Apparent HR 1.575/1.612/1.602; out-of-fold HR 1.014 (*p* = 0.911), 1.198 (*p* = 0.146) and 1.361 (1.190–1.557), *p* = 7.05 × 10^−6^; permutation empirical *p* 0.809, 0.688 and 9.99 × 10^−4^. STRICT is the pre-registered primary and is not validated out-of-fold; PAN-HNSC is the sensitivity variant and is the only cohort that survives. (**C**) Apparent Cox hazard ratios for all fifteen cohort × signature cells, with BH-corrected *p* across the fifteen tests. The ssGSVA EMT hazard ratios move from below 1 to above 1 in STRICT and BROAD once the 13 solid-tissue normals are removed, and four ssGSVA cells reach nominal *p* < 0.05, but none survive BH correction. All hazard ratios are per standardised z-unit, multivariable-adjusted for age and sex. External application of the frozen signatures to the independent GSE41613 cohort is reported in Figure S7H.

Three pre-registered signatures were applied to three nested cohort variants, restricted to primary tumours and with the Coxnet coefficients refit (Section 2.2.6). All fifteen cohort × signature cells are reported (Figure 7C).

Apparent in-sample results: The anchor-excluded LASSO–Cox signature separated overall survival in all three variants, and more strongly than we originally reported: STRICT (n = 171; 65 events) HR = 1.575 (95% CI 1.286–1.929), Cox *p* = 1.13 × 10^−5^, log-rank *p* = 5.81 × 10^−5^; BROAD (n = 181; 67 events) HR = 1.612 (1.299–2.001), Cox *p* = 1.44 × 10^−5^, log-rank *p* = 5.42 × 10^−5^; PAN-HNSC (n = 512; 215 events) apparent HR = 1.602 (1.389–1.847), Cox *p* = 8.50 × 10^−11^, log-rank *p* = 1.71 × 10^−10^ (Figure 7A,B). We no longer describe this as stratification “with high consistency”: the cohorts are strictly nested (primary-tumour Jaccard 0.934, 0.353 and 0.330; BROAD adds twelve tumours to STRICT), so agreement between them is not evidence.

*The primary z-sum and ssGSVA variants*: The z-sum signature reached HR = 1.307 (1.133–1.509), Cox *p* = 2.51 × 10^−4^ in PAN-HNSC, and HR = 1.259 (*p* = 0.071) and 1.267 (*p* = 0.060) in STRICT and BROAD. The Hallmark ssGSVA variants reached nominal *p* < 0.05 in 4 of 9 cohort × variant cells (STRICT G2-M *p* = 0.044; BROAD G2-M *p* = 0.019; PAN EMT *p* = 0.035; PAN Myc Targets V1 *p* = 0.047), none of which survives Benjamini–Hochberg correction across the fifteen cohort × signature tests (BH *p* 0.056–0.087); the three LASSO–Cox cells and PAN z-sum do survive. Both of the comparative statements printed—that z-sum was significant “only in PAN-HNSC” at marginal level, and that ssGSVA was “non-significant across all three cohorts”—are therefore withdrawn. The ssGSVA EMT hazard ratios also move from below one to above one in STRICT and BROAD once the 13 solid-tissue normals are removed.

*Internal validation and what it shows*: With the entire selection procedure re-executed inside every training fold, apparent C-indices of 0.651/0.633/0.635 fall to cross-validated 0.506 ± 0.021 (STRICT), 0.518 ± 0.021 (BROAD) and 0.568 ± 0.013 (PAN-HNSC) (Figure S7A). Bootstrap optimism correction, which estimates optimism differently and should not be read off the same subtraction, gives 0.557, 0.543 and 0.607 from mean optimism of 0.094, 0.090 and 0.028. Out-of-fold hazard ratios are 1.014 (0.789–1.304, *p* = 0.911), 1.198 (0.939–1.528, *p* = 0.146) and 1.361 (1.190–1.557, *p* = 7.0 × 10^−6^).

The permutation nulls are the decisive result. Under 1000 permutations of the survival labels, the same selection-plus-fit pipeline reached Cox *p* < 0.05 in 100.0%, 99.9% and 100.0% of runs (Figure S7C), and in STRICT and BROAD the median permuted hazard ratio (1.702 and 1.696) exceeded the observed value, giving empirical *p* = 0.809 and 0.688; PAN-HNSC gave empirical *p* = 9.99 × 10^−4^ against a null median HR of 1.329 (Figure S7B). Evaluated out-of-fold, the same procedure is correctly calibrated (0.5%, 0.5% and 1.5% of permuted runs significant), and the out-of-fold permutation *p*-values are 0.428, 0.229 and 0.005 (Figure S7D). Feature-selection stability tracks this: all six PAN-HNSC features were retained in ≥64% of the 25 cross-validation folds, whereas five of STRICT’s nine were retained in fewer than half (Figure S7F), and the three cohorts share only DKK1, PCOLCE2 and SFRP1 (Figure S7G).

*External validation*: External validation reproduced the internal verdict. Applying the frozen TCGA coefficients to GSE41613 (97 HPV-negative oral-cavity SCC, 51 deaths; Section 2.2.6) without any refitting, the pre-registered primary STRICT signature did not validate: C = 0.562 (95% CI 0.475–0.649, an interval that includes 0.50), median-split log-rank *p* = 0.301, and an age- and sex-adjusted hazard ratio of 1.280 per standard deviation (0.966–1.697, *p* = 0.085)—the correct direction, but a non-significant trend. The PAN-HNSC sensitivity signature, the only one of the three to survive internal cross-validation and permutation testing, did discriminate: C = 0.625 (0.530–0.708, excluding 0.50), adjusted HR 1.630 per standard deviation (1.176–2.259, *p* = 0.0034), and 1.665 (1.165–2.381, *p* = 0.0052) after further adjustment for tumour stage and treatment modality. BROAD, null internally, was null externally (C = 0.538; adjusted HR 1.125, 0.855–1.480, *p* = 0.398). Results were essentially unchanged without gene-wise standardisation (PAN-HNSC C = 0.627; Spearman ρ = 0.986 between the two scores), and the PAN-HNSC adjusted *p* survives Bonferroni correction over all six pre-declared cells (0.020) and over the three distinct signatures (0.010) (Figure S7H; Table S9.4). Two constraints are stated with the results. First, the median-split log-rank test was non-significant for every variant, including PAN-HNSC (*p* = 0.067), so the data support prognostic information in the continuous score but not a dichotomised high-/low-risk classification. Second, there is a scope mismatch: GSE41613 is oral-cavity and HPV-negative, which matches STRICT’s scope, whereas the validated signature was fitted to the anatomically unrestricted, partly HPV-positive cohort, so the transfer runs across scope. We therefore retain STRICT as the pre-registered primary analysis and report it as null; promoting PAN-HNSC on the strength of this cohort would be an outcome-dependent selection of exactly the kind this revision sets out to avoid (Section 4.4).

The result we take forward is therefore narrow: a prognostic association that survives out-of-fold evaluation, and now external application, in the pan-HNSC sensitivity cohort only, and not in the pre-registered oral-cavity cohorts on which this paper’s OSCC framing depends. It is reported as a supporting observation rather than a headline claim (Section 4.4).

### 3.7. Pathway, Transcription-Factor, Immune-Compartment and Anchor-Excluded Gene-Set Analyses of the High-Anchor Stratum (Figure 8)

All results in this section are computed on the 171 STRICT primary tumours.

**Figure 8:**
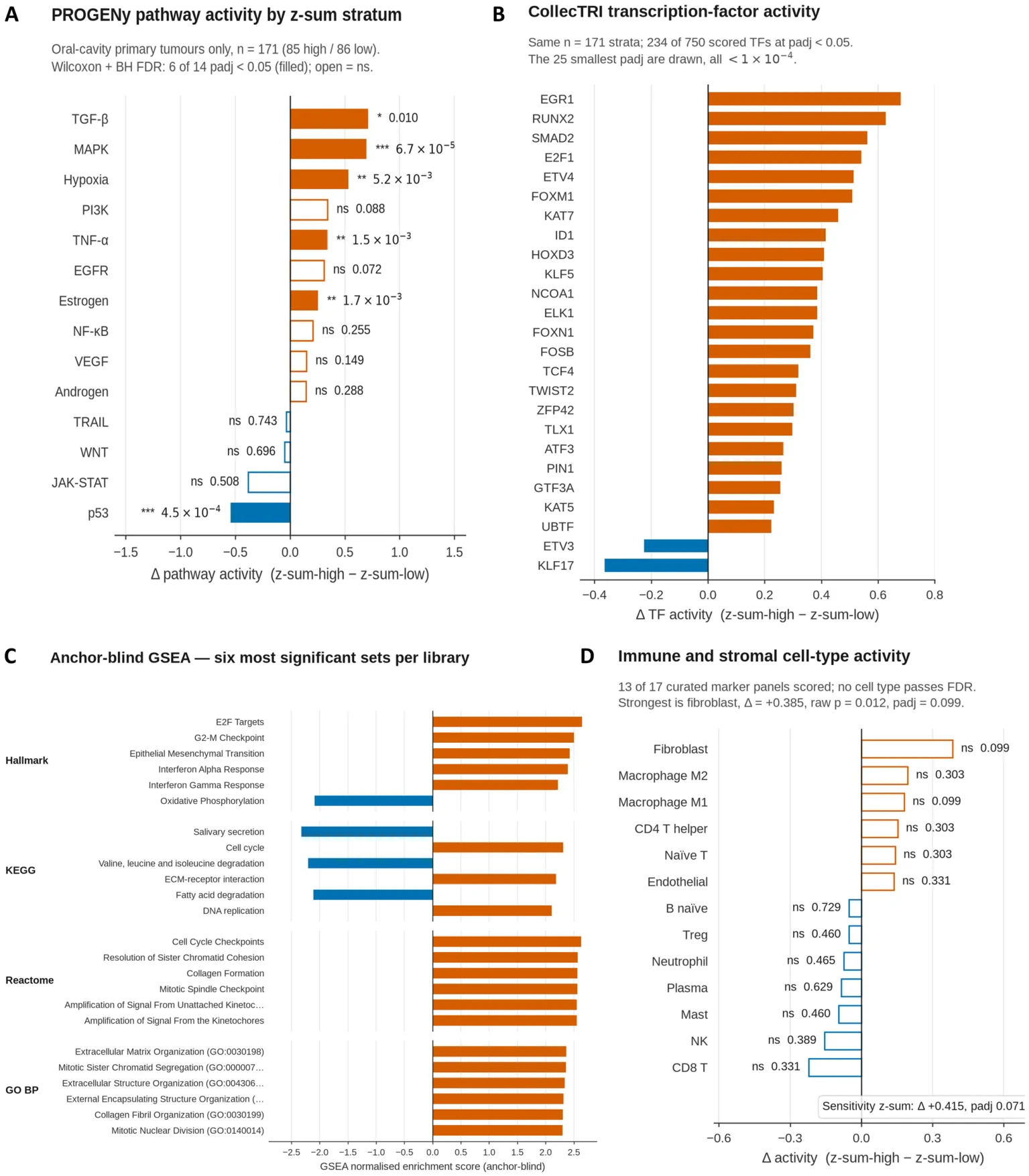
**Pathway, transcription-factor, gene-set and immune-compartment analyses of the high-anchor stratum (171 STRICT primary tumours).** (**A**) PROGENy pathway activity, high minus low z-sum stratum. MAPK, TGF-β, Hypoxia, TNF-α and Oestrogen are elevated, and p53 is reduced at padj < 0.011; EGFR, PI3K and VEGF share the direction but do not survive FDR; JAK-STAT and NF-κB are not significant. * padj < 0.05; ** padj < 0.01; *** padj < 0.001 (Benjamini–Hochberg adjusted). (**B**) CollecTRI transcription-factor activity, top differentially active factors; KLF17 (anti-EMT) is the most significant and is reduced. (**C**) Anchor-excluded GSEA normalised enrichment scores across four gene-set libraries, with the eight anchors removed from both the ranking statistic and gene-set membership. (**D**) Marker-based immune-compartment activity (13 of 17 curated cell types scored). Fibroblast and macrophage M1 are the joint-strongest signals at BH padj = 0.099; no cell type reaches FDR < 0.05, so the CAF-enriched, CD8-low pattern is reported as a trend.

*PROGENy pathway activity (Figure 8A)*: In the z-sum-high stratum, MAPK (Δ +0.698, padj 6.7 × 10^−5^), TGF-β (+0.714, 0.010), Hypoxia (+0.531, 5.2 × 10^−3^), TNF-α (+0.340, 1.5 × 10^−3^) and Oestrogen (+0.255, 1.7 × 10^−3^) were elevated, and p53 was reduced (−0.545, 4.5 × 10^−4^), all at padj < 0.011. EGFR (+0.311, padj 0.072), PI3K (+0.342, padj 0.088), and VEGF (+0.150, padj 0.149) show the same direction but do not survive FDR correction. JAK-STAT and NF-κB are not significantly different (padj 0.508 and 0.255). Three separate errors in the earlier sentence are corrected here, two of which predate the cohort correction: JAK-STAT was never significant (padj was 0.807 in the earlier output itself) and its direction now reverses to −0.382; PI3K was UP (+0.606) and among the most significant pathways in the earlier output (padj 0.0024), so “slightly down” had the sign inverted; and VEGF and EGFR fall below the FDR threshold. Only p53 was correctly described as reduced.

*Transcription-factor activity (Figure 8B)*: CollecTRI inference on the same stratum shows coordinated activation of EGR1 (Δ +0.681, padj 2.1 × 10^−5^), RUNX2 (+0.628, 2.6 × 10^−6^), SMAD2 (+0.562, 2.3 × 10^−5^), E2F1 (+0.541, 5.3 × 10^−5^), ETV4 (+0.513, 6.7 × 10^−5^), FOXM1 (+0.510, 1.2 × 10^−7^), KAT7 (+0.459, 2.1 × 10^−5^), TCF7L2 (+0.428, 1.2 × 10^−4^), ID1 (+0.415, 2.6 × 10^−6^), KLF5 (+0.406, 4.1 × 10^−6^) and ELK1 (+0.385, 2.7 × 10^−6^), and reduced activity of the anti-EMT factor KLF17 (−0.364, padj 9.3 × 10^−9^), the single most significant transcription factor in the corrected analysis, together with ETV3 (−0.226, 9.1 × 10^−6^). The direction of every factor named here was checked against the re-rendered Figure 8B independently of the writer.

*Immune-compartment activity (Figure 8D)*: Marker-based ULM activity against the curated panel—13 of the 17 cell types were scored, cDC1, cDC2, pDC and Pericyte having been dropped by min_npruning in the earlier analysis as well as this one—shows the z-sum-high stratum trending towards fibroblast enrichment and CD8 depletion without reaching significance. Fibroblast activity was higher in the high stratum (Δ = +0.385, raw *p* = 0.012, BH padj = 0.099; a sensitivity analysis holding the published z-sum fixed and row-filtering gives Δ = +0.415, padj = 0.071). Macrophage M1 is now the joint-strongest signal alongside fibroblast (Δ = +0.181, *p* = 0.015, padj = 0.099). CD4 T helper Δ = +0.153 (*p* = 0.117, padj = 0.303), macrophage M2 Δ = +0.196 (*p* = 0.096, padj = 0.303) and CD8 T Δ = −0.222 (*p* = 0.178, padj = 0.331). The submitted heading described a “CAF-enriched, CD8-low” tumour microenvironment on the strength of a padj of 4.9 × 10^−3^; under either cohort variant, this is a trend rather than a significant finding, and only the direction is being claimed. That direction is consistent with the documented immunosuppressed phenotype of high-EMT OSCC, and no more than that.

Anchor-excluded GSEA (Figure 8C): With the eight anchors removed from both the ranking statistic and gene-set membership, pre-ranked GSEA independently recovered the same programme: Hallmark E2F Targets NES = +2.64, G2-M Checkpoint +2.50, EMT +2.42, Interferon-α Response +2.39 and Interferon-γ Response +2.22, with Oxidative Phosphorylation down at −2.09; Reactome Cell Cycle Checkpoints +2.63, resolution of Sister Chromatid Cohesion +2.57, collagen formation +2.56 and Mitotic Spindle Checkpoint +2.56; KEGG cell cycle +2.31, ECM-receptor interaction +2.19 and DNA replication +2.11; GO-BP extracellular matrix organisation +2.36 and Mitotic Sister Chromatid Segregation +2.36 (all at FDR = 0.001). Across libraries, 28 Hallmark sets, 88 KEGG, 386 Reactome and 168 GO-BP sets were significant at FDR < 0.05. Fifteen gene sets fell below five members after anchor exclusion and were dropped per protocol (Figure S6A,B; Supplementary Table S8). This layer is unaffected by the cohort correction because it consumes the L1 differential-expression ranking rather than the survival strata; it is, however, derived from the same L1 output as Section 3.2 and is not independent of it (Table 3).

### 3.8. Stromal–Tumour Ligand–Receptor Inference in Single-Cell Data (Figure 9 and Figure S3)

**Figure 9:**
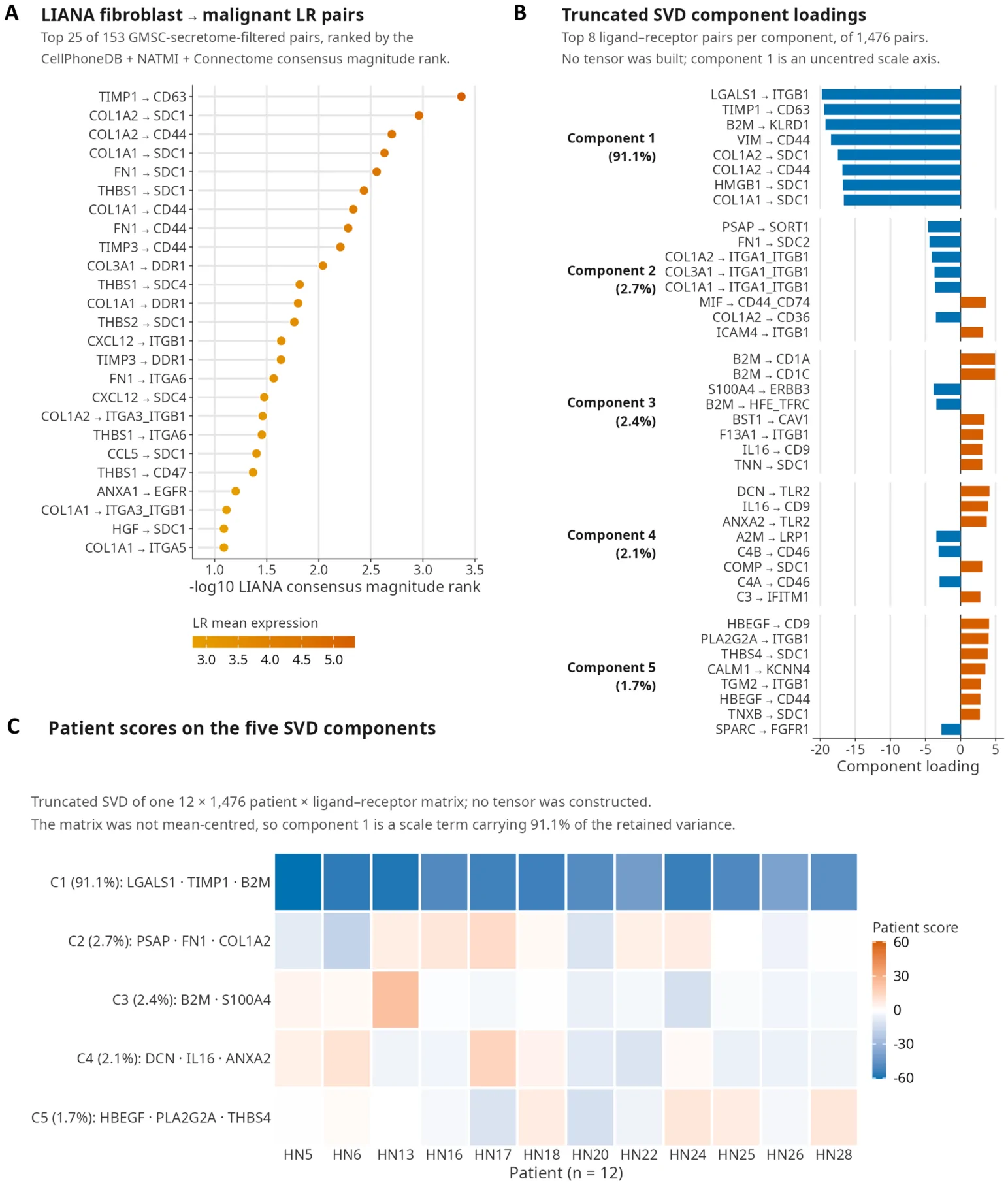
**LIANA cell–cell communication and truncated SVD of the patient × LR-pair matrix.** (**A**) LIANA consensus dot plot of the top 25 fibroblast → malignant ligand–receptor pairs after GMSC-secretome ligand filtering on GSE103322; dot size = −log_10_(magnitude rank), colour = LR mean. (**B**) Component loadings of the truncated SVD, top 8 LR pairs per component (40 of 1476 rows shown). No tensor was constructed. (**C**) Patient × component scores (12 patients × 5 components). Component 1 carries 91.1% of the retained sum of squares with all 12 patient loadings and all 1476 LR loadings of one sign, because the matrix was not mean-centred: it is an overall-magnitude axis, not a latent communication pattern. Components 2–5 carry 2.7%, 2.4%, 2.1% and 1.7%.

LIANA consensus aggregation (CellPhoneDB + NATMI + Connectome) on GSE103322 returned 841 fibroblast → malignant ligand–receptor pairs, of which 153 had ligands present in the curated GMSC secretome; these 153 constitute the pre-specified universe carried into the spatial analysis (Section 3.9). Top pairs by consensus magnitude rank were TIMP1 → CD63, COL1A2 → SDC1, COL1A2 → CD44, COL1A1 → SDC1, FN1 → SDC1, THBS1 → SDC1, COL1A1 → CD44, FN1 → CD44, TIMP3 → CD44, COL3A1 → DDR1, THBS1 → SDC4, COL1A1 → DDR1, THBS2 → SDC1, CXCL12 → ITGB1 and TIMP3 → DDR1 (Figure 9A; top 50 in Figure S3A).

The truncated SVD of the 12 patients × 1476 LR-pair score matrix retained five components (Figure 9B,C and Figure S3B). Component 1 carries 91.1% of the retained sum of squares, with all 12 patient loadings and all 1476 ligand–receptor loadings of the same sign, because the matrix was not mean-centred: it is an overall-magnitude axis, not a latent communication pattern, and components 2–5 account for only 2.7%, 2.4%, 2.1% and 1.7%. The submitted description of five latent factors “capturing patient-specific CCC patterns” overstated what this decomposition provides, and the term “tensor decomposition” has been removed throughout, no tensor having been constructed (Section 2.2.8).

### 3.9. Spatial Examination of the Predicted Ligand–Receptor Pairs Shows No Set-Level Enrichment (Figure 11B–D)

In GSE300414 (24 patients, 133 ROIs, 289 tissue AOIs of which 276 passed QC: 126 stromal, 125 tumour and 25 immune), all 153 GMSC-secretome-filtered fibroblast → malignant pairs were tested by correlating stromal-AOI ligand expression with tumour-AOI receptor expression per patient, on Q3-normalised, log_2_-transformed counts.

There is no set-level enrichment. In total, 3 of 153 pairs (2.0%) exceeded ρ > 0.6, against a 10,000-draw non-cognate ligand × receptor permutation background of 3.29% (95% CI 2.95–3.66%); binomial *p* = 0.88. The whole ρ distribution is indistinguishable from the null (Mann–Whitney *p* = 0.58; observed median ρ +0.088 versus null +0.091), and a decile-matched null gives 2.35%, *p* = 0.70 (Figure 11D). Under an unnormalised pipeline, 92.8% of random pairs also exceeded ρ > 0.6, so the reported figure of 23 of 30 (76.7%) was below the random background of the very analysis that produced it (Figure 11C). The cause is depth: AOI library size spans from 0 to 5.01 M, and stromal and tumour depth co-vary at ρ = 0.921 across the 24 patients.

Three pairs reach FDR < 0.05 on the nominal Spearman statistic (Figure 11B): TIMP1 → CD63 (ρ = +0.811, *p* = 1.5 × 10^−6^, BH padj = 2.3 × 10^−4^), THBS1 → SDC4 (+0.704, 1.2 × 10^−4^, 9.3 × 10^−3^) and THBS2 → SDC1 (+0.681, 2.5 × 10^−4^, 1.3 × 10^−2^). TIMP1 → CD63 is stronger after correction than before (0.764 as published), so this is not a wholesale collapse. None of the three survives FDR once the *p*-value is calibrated against the permutation null (the best is TIMP1 → CD63 at empirical FDR 0.076), and 0 of 153 pairs do. The parametric Spearman *p* is approximately 34× anti-conservative in this design. A fourth pair, ANXA2 → TLR2, reaches nominal FDR at ρ = −0.639: it is anti-correlated, not co-localised, and was +0.606 on raw counts.

Four of the previously highlighted co-localisations do not reach significance: TIMP2 → ITGB1 +0.351 (*p* = 0.092); ANXA1 → EGFR, which moves from ρ = 0.79 (*p* = 5.1 × 10^−6^) to +0.199 (*p* = 0.351); COL1A1 → SDC1 +0.552 (*p* = 5.2 × 10^−3^, padj = 0.073); HGF → SDC1 +0.001 (*p* = 0.997). Most consequentially, the single pair involving a wet-lab anchor, CXCL12 → ITGB1, moves from ρ = 0.70 (*p* = 1.5 × 10^−4^) to ρ = −0.030 (*p* = 0.891, padj = 0.947, empirical *p* = 0.648), and across all six sensitivity variants its ρ ranges from −0.061 to +0.128 without ever approaching significance. We note that removing ANXA1 → EGFR also removes the only spatial support for EGFR as a receiving receptor in this study.

We withdraw the claim that this cohort constitutes independent validation. Its entire candidate space is L10’s output (L15’s ligands are a strict subset of L10’s, and every pair tested is an L10 pair), so it is a check of L10, not a prior independent of it (Table 3). We also withdraw the statement that the cohort “recapitulates the LR ranking”, but on the correct grounds. Across all 153 pairs, the LIANA magnitude rank is in fact weakly associated with the spatial correlation in the supportive direction (Spearman −0.262, *p* = 0.001; specificity rank −0.290, *p* = 3 × 10^−4^). That association is fully attributable to transcript abundance: conditioning on the mean GeoMx abundance of both partners reduces it to a partial Spearman of −0.059 (*p* = 0.47), LIANA rank correlating with ligand abundance at −0.475 and with receptor abundance at −0.528, and receptor abundance correlating with ρ at +0.322. For completeness, on raw counts the same 153-pair association has the anti-supportive sign (+0.284, *p* = 3.7 × 10^−4^). We therefore no longer claim that the spatial data recapitulate the ligand–receptor ranking, while reporting that a nominally supportive association exists and does not survive abundance conditioning.

### 3.10. A Direction-Resolved LINCS L1000 Screen Nominates Gefitinib as a Hypothesis-Generating Candidate (Figure 10 and Figure S4)

**Figure 10:**
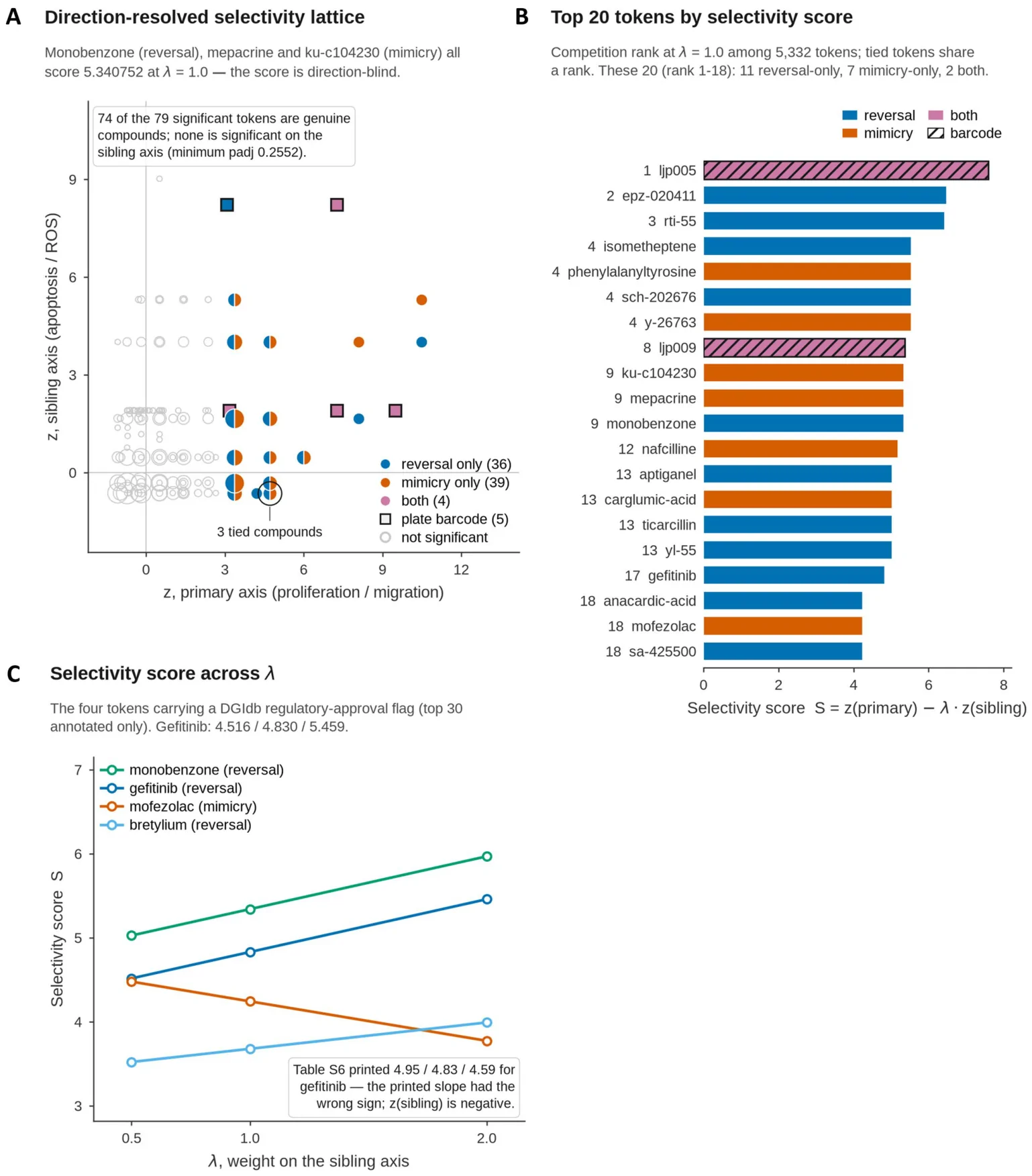
**Direction-resolved LINCS L1000 connectivity screen.** (**A**) Score lattice: x = z(primary, proliferation/migration connectivity), y = z(companion, apoptosis/ROS connectivity), coloured by the resolved geometry of each token’s significant primary-axis terms. No quadrant scheme is drawn: 0 of 5332 tokens reach padj < 0.05 anywhere on the companion axis (minimum padj 0.2552) and 79 (1.48%) reach it on the primary axis, and the lattice is discrete—53 distinct z(primary) × 15 distinct z(companion) values yield only 140 distinct λ = 1.0 scores. Gefitinib is reversal-only, z 4.202/−0.628, S(λ = 1) 4.830, rank 17 of 5332. Monobenzone (reversal), mepacrine, and ku-c104230 (mimicry) share an identical score of 5.340752. (**B**) Top 20 by score at λ = 1.0, annotated with resolved geometry and DGIdb regulatory-approval flag; hatched bars are LINCS plate barcodes, not compounds. (**C**) Score across λ ∈ {0.5, 1.0, 2.0} for the four tokens carrying a DGIdb approval flag; gefitinib 4.516/4.830/5.459, and monobenzone is ahead at every λ. The score cannot distinguish reversal from mimicry: among all 79 significant tokens, 36 are reversal-only, 39 mimicry-only, and 4 both (all plate barcodes); among the 74 genuine compounds, 35 and 39; among the top 30 at λ = 1.0, 15 and 13.

The proliferation/migration contrast was queried against the LINCS L1000 chemical-perturbation libraries in parallel with the companion apoptosis × ROS contrast [13], anchors excluded from both (Figure 10A). The compound universe is approximately 5297 LINCS consensus perturbagens plus 25 plate identifiers admitted in error by our name parser (Section 2.2.9), the remaining tokens being unresolved name variants; no corrected output reproduces a coverage figure of 5064 of 5332 tokens (95%) with connectivity data for both axes.

The scoring statistic cannot distinguish reversal from mimicry, and roughly half the significant compounds are mimicry. Of the 74 genuine compounds with any significant primary-contrast term, 35 (47.3%) are reversal-only, and 39 (52.7%) are mimicry-only; within the top 30 of the reported ranking, 15 are reversal-only, 13 mimicry-only, and two are significant in both—the latter two being plate barcodes. The submitted text described the top compounds as “drugs that reverse the pro-proliferation/migration shift while having minimal reversing effect on the protective apoptotic shift” and named mepacrine among them; mepacrine is mimicry-only and does the opposite of what that sentence claims. The clearest demonstration that the score is direction-blind is that mepacrine, monobenzone and ku-c104230 all score exactly 5.340752 at λ = 1.0 (identical z_primary 4.712632, z_companion −0.628120, padj 0.011816) with opposite geometry (Figure 10A).

*Gefitinib’s numbers and their context.* Gefitinib scores selectivity of 4.830408 at λ = 1.0 (z_primary = 4.202288, z_companion = −0.628120) and ranks 17 of 5332 at λ = 1.0 (18 at λ = 0.5, 5 at λ = 2.0). Only 79 of 5332 tokens (1.48%) reach padj < 0.05 on the primary contrast, so z_primary = 4.20 is a rank position against a 98.5% null background, and ljp005 is rank 1 and ljp009 is rank 8—two of the top eight “compounds” are plate barcodes (Figure 10B). The λ-sweep’s apparent rank stability is partly a tie artefact: there are only 53 distinct z_primary values, 15 distinct z_companion values and 140 distinct λ = 1.0 selectivity values across 5332 tokens, so rank differences in a few places are not meaningful (Figure 10C). We also correct the λ profile itself: gefitinib’s selectivity is 4.516348/4.830408/5.458528 at λ = 0.5/1.0/2.0. Supplementary Table S6 printed 4.95/4.83/4.59, a profile that decreases with λ, whereas the true profile increases, because z_companion is negative; only the λ = 1.0 value was correct.

*The direction is correct, and the support is thin.* In the direction-resolved re-analysis, gefitinib’s single BH-significant primary-contrast association is the LINCS consensus Gefitinib Down set intersecting the tumour-up gene list—reversal geometry, the direction our interpretation requires (9 of 245 genes, *p* = 3.9 × 10^−6^, padj = 0.017). Three qualifications attach to it. The association is carried by seven MAGE-family cancer-testis antigens (MAGEA1, MAGEA3, MAGEA4, MAGEA6, MAGEA12, MAGEB2, MAGEC2, alongside HMGA2 and PAEP) and does not survive their removal (*p* = 0.30, against a BH threshold of 1.1 × 10^−5^ at that rank). It is not replicated in the per-signature libraries: across 78 gefitinib signatures, the split is 25 reversals to 17 mimicry against a 51.3% baseline (binomial *p* = 0.18), with no individual signature at padj < 0.05. And no EGFR-pathway gene appears among the drivers, nor could one: EGFR is one of the eight excluded anchors, so this analysis was blind to EGFR by construction. Gefitinib does have 67 DGIdb-curated interactions including EGFR and HER-family members (verified and retained), but that fact is a property of the drug, not evidence produced by the screen.

Approval annotation: DGIdb v5 provides a general regulatory-approval flag, not one specific to any single national regulator, and returned ATC class and approval year for 0 of 30 queried compounds; the ATC code L01EB01 quoted for gefitinib is sourced from the WHO ATC index. Only four compounds in the annotated set carry the approval flag, and monobenzone outranks gefitinib at every λ (competition ranks 11/9/1 versus 18/17/5). The submitted supplementary note claiming gefitinib was “rank 1 among FDA-approved compounds” is false and was falsified by the table printing it. A reversal-only re-ranking of the 4901 tokens carrying any reversal term places monobenzone fifth and gefitinib ninth, behind sch-202676, rti-55, epz-020411 and isometheptene.

### 3.11. DepMap CRISPR Essentiality: EGFR Is the One Lineage-Selective Dependency Among the Predicted Targets (Figure 11A and Figure S5; Table 4)

L12-predicted drug targets and the eight anchors (90 unique genes, of which 88 had DepMap Public 24Q4 Chronos data) were cross-referenced against 53 oral-cavity SCC lines as the primary stratum, 77 head-and-neck-lineage lines as a sensitivity stratum and the full 1178-line 24Q4 screened panel as the all-lineage reference. The panel used previously contained 11 non-head-and-neck cell lines (12.5%), introduced by a hard-coded ModelID list that was unioned with, rather than substituted for, the lineage filter; this is a data-integrity error that no reviewer raised, and we report and correct it here. The correction is conservative in aggregate—Spearman ρ = 0.9971 between published and corrected medians across the 88 measured genes, median |Δmedian| = 0.0074, maximum 0.082 (CRKL)—but it changes the rank order at positions 3–7 and the membership of the essential set.

Twelve genes cross the −0.5 essentiality threshold in the oral-cavity stratum. A median gene effect measured in one lineage is not, on its own, evidence about that lineage, because a gene that almost every cell line requires will clear the threshold in whatever panel is assembled. The question each gene has to answer is whether it is a stronger dependency in oral-cavity SCC than in cancer cell lines generally, so all twelve are annotated with the DepMap 24Q4 Common Essential flag as shipped with the release and with the difference between the oral-cavity median and the all-lineage median (Table 4).

One gene separates on both criteria. EGFR does not carry the Common Essential flag, and it has the largest lineage selectivity of the twelve: median Chronos −0.677 across the 53 oral-cavity SCC lines against −0.189 across all 1178 screened lines, a difference of −0.488, and essentiality in 69.8% of oral-cavity lines (37/53) against 19.0% of lines overall. It is the only gene in the set whose dependency is close to lineage-restricted rather than general, and it is the gene on which the target-level argument of Section 4.5 rests.

Seven of the twelve carry the Common Essential flag: PCNA, MYC, MTOR, CRKL, EIF4A1, NUP62 and CDK4. For these genes, appearing in an oral-cavity ranking is the expected behaviour of a gene that nearly every screened line requires, and it is not evidence about oral-cavity SCC. PCNA heads the ranking at −2.864 and is essential in 53 of 53 oral-cavity lines, but it is also essential in 99.9% of all 1178 screened lines, an oral-cavity-minus-all-lineage difference of −0.115; MTOR and EIF4A1 are in fact marginally less depleted in oral cavity than across lineages (+0.031 and +0.149). CRKL is the one Common Essential gene with an appreciable difference (−0.308; essential in 94.3% of oral-cavity lines against 74.9% overall). Of the five genes carrying no Common Essential flag, the four other than EGFR are intermediate (CDK6 −0.335, RELA −0.181, PIK3CA −0.127 and CCND1 +0.024) and none approaches EGFR’s separation.

Table 4 also reports normLRT, the statistic underlying DepMap’s Strongly Selective designation, in our own reimplementation (Section 2.2.11). It is not the portal value, and it is not the measure of selectivity used here. normLRT describes the shape of a gene’s dependency distribution across all screened lines and is indifferent to which lines the dependency falls on, so a gene can be pan-essential and strongly skewed at once: CDK4 carries the Common Essential flag, a normLRT of 569.1, and an oral-cavity-minus-all-lineage difference of +0.039, which is to say no oral-cavity selectivity at all. The selectivity column is what answers the lineage question, and it requires no distributional fit.

The full rank order in the oral-cavity stratum is given in Table 4. GMNN reaches the essential set in neither stratum (−0.497 in oral cavity, −0.491 in head and neck), while RELA reaches it in the oral-cavity stratum only (−0.549 there against −0.492 in the 77-line stratum), so RELA is reported as borderline and possibly OSCC-specific. Twelve genes cross the −0.5 threshold in the oral-cavity stratum and eleven in the head-and-neck stratum, and beyond the first five, no gene is strongly essential (<−1.0) in the majority of lines.

The layer therefore supports a narrower statement than the rank order alone would suggest. PCNA, MYC, MTOR, CRKL and EIF4A1 are strong dependencies in oral-cavity SCC lines, but they are strong dependencies almost everywhere, and their presence here carries no information specific to OSCC. What this layer contributes to the argument is EGFR: a dependency in roughly seven of every ten oral-cavity SCC lines that is a dependency in fewer than one line in five across all lineages. That is target-level support and nothing more. It does not show that a drug directed at EGFR reverses the GMSC-induced programme (Table 3), and the connectivity screen that nominated gefitinib was blind to EGFR by construction (Section 3.10 and Section 4.5).

### 3.12. Integrative Model: Three Lines of Evidence (Figure 11)

Three lines of evidence bear on the paracrine argument, and each is stated here with its scope. Two candidate lines do not survive testing and are withdrawn, and two further items that could be mistaken for evidence are not evidence at all; all four are set out after the list.

1. A prognostic association, out of fold, in the pan-HNSC sensitivity cohort only: n = 512; cross-validated C = 0.568; out -of -fold HR = 1.36 (1.19–1.56), *p* = 7.0 × 10^−6^; out-of-fold permutation *p* = 0.005—and not in the pre-registered oral-cavity cohorts, where the same pipeline reaches Cox *p* < 0.05 in 100% of label-permuted runs (Section 3.6).
2. Lineage-enriched CRISPR dependency in oral-cavity SCC lines, which is a narrow line and must be stated as one. Of the twelve genes crossing the essentiality threshold, four (PCNA, MYC, CCND1 and EGFR) are themselves wet-lab anchors, and a dependency measured on an anchor cannot count as independent support for the programme those anchors define; five of the remaining eight (MTOR, CRKL, EIF4A1, NUP62 and CDK4) carry the DepMap 24Q4 Common Essential flag, so their essentiality here is the expected behaviour of a pan-essential gene rather than a statement about oral-cavity SCC. What is left is a modest lineage-enriched signal in three non-anchor, non-Common Essential genes: CDK6 (median Chronos −0.839; essential in 77.4% of the 53 oral-cavity lines against 50.5% across all screened lineages), PIK3CA (−0.553; 56.6% against 39.0%) and RELA (−0.549; 50.9% against 26.7%). EGFR is the most lineage-selective gene in the panel (−0.677 in oral cavity against −0.189 across all lineages), but it is an anchor, so it supports the target and not the argument (Section 3.11 and Section 4.5).
3. A described candidate ligand–receptor geometry over the curated 46-ligand secretome, presented as coverage of a pre-registered candidate space by three mechanistically distinct rationales, with a spatially consistent THBS/TIMP–syndecan subset that does not reach set-level significance (Section 3.4, Section 3.8 and Section 3.9).

Two candidate lines are withdrawn. The convergence of NicheNet, STRING and LIANA “on a single canonical GMSC paracrine ligand set” is agreement at chance level (*p* = 0.091) with no rank agreement (Kendall’s W = 0.312, *p* = 0.541), and the GeoMx cohort shows no set-level enrichment (3 of 153 versus a 3.29% background, *p* = 0.88), has no pair surviving FDR against the permutation null, and was never independent of LIANA.

**Figure 11:**
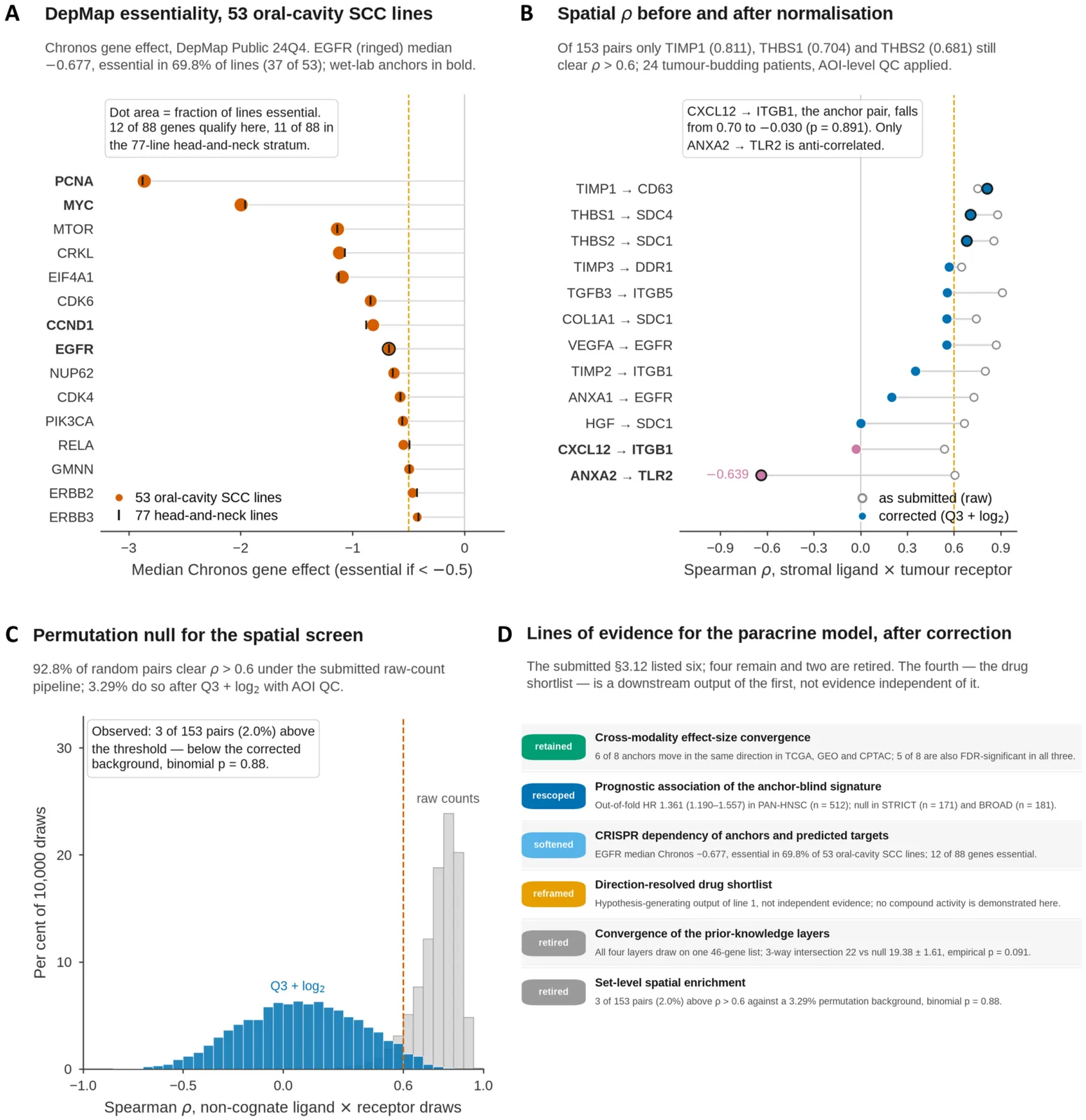
**DepMap CRISPR essentiality, spatial ligand–receptor examination and the permutation calibration of both spatial pipelines.** (**A**) Median Chronos gene effect across 53 oral-cavity SCC lines (dot area scaled to the fraction of essential lines; tick marks the 77-line head-and-neck sensitivity stratum; wet-lab anchors in bold; EGFR ringed). Twelve genes cross the −0.5 threshold in this stratum and eleven in the 77-line stratum, RELA crossing in the oral-cavity stratum only and GMNN in neither. The panel plots the oral-cavity stratum alone; the DepMap 24Q4 Common Essential flag and the all-lineage comparison that separates lineage-relevant from pan-essential dependency are given in Table 4. Seven of the twelve are Common Essential, PCNA among them (−2.864 here and essential in 53 of 53 oral-cavity lines, but also essential in 99.9% of all 1178 screened lines). EGFR is not: −0.677 here against −0.189 across all lineages, essential in 69.8% of oral-cavity lines (37/53) against 19.0% overall, the largest lineage selectivity of the twelve. The 53-line panel is defined by Oncotree annotation alone (Section 2.2.11 and Section 3.11). The dashed line marks the Chronos = −0.5 threshold conventionally used to call a gene essential. (**B**) Spatial Spearman ρ before and after normalisation for the named ligand–receptor pairs: open circles are raw DCC counts and filled circles are Q3 + log2 with AOI QC. A filled circle is blue where the corrected coefficient remains positive and pink where it is negative: CXCL12 → ITGB1 (ρ = −0.030) and ANXA2 → TLR2 (ρ = −0.639), the latter∙s printed value being in the same pink. A ring marks BH padj < 0.05 on the nominal statistic; bold type marks the two pairs discussed in the text, the wet-lab anchor pair CXCL12 → ITGB1 and the anti-correlated pair ANXA2 → TLR2. Only TIMP1 → CD63, THBS1 → SDC4 and THBS2 → SDC1 reach nominal FDR; ANXA2 → TLR2 reaches it while anti-correlated; and CXCL12 → ITGB1, the only pair involving a wet-lab anchor, moves from 0.70 (*p* = 1.5 × 10^−4^) to −0.030 (*p* = 0.891). Points are coloured by ligand–receptor pair family; the filled points are the pairs retained after Q3 normalisation and the open points are those that do not survive it. (**C**) Permutation null of the earlier pipeline: 92.76% of 10,000 non-cognate ligand × receptor draws exceed ρ > 0.6 on raw counts, so the reported 23 of 30 (76.7%) is *below* its own background (binomial *p* = 0.999); stromal and tumour library size co-vary at ρ = 0.921 across the 24 patients. (**D**) Permutation null of the corrected pipeline: background 3.29% [2.95–3.66], observed 3 of 153 (2.0%), binomial *p* = 0.882; whole-distribution Mann–Whitney *p* = 0.579; the nominal Spearman *p* is ~34× anti-conservative, and 0 of 153 pairs survive FDR against this null.

Two further items sit outside the list because they are not evidence for the paracrine argument. The first is the gefitinib nomination: it is a downstream output of the direction-resolved connectivity screen (L12) run on the L1 tumour contrast, and it is prior-motivated rather than screen-discovered (Section 3.10 and Section 4.5)—an output of the framework cannot also be an input to it. The second is the three-way agreement across TCGA RNA-seq, the GEO meta-analysis and CPTAC proteomics: 1219 of the 1358 genes significant in all three modalities (89.8%, 95% CI 88.0–91.3%) are sign-concordant in all three against 25% expected by chance, and six of eight anchors are direction-concordant in all three (Section 3.3). All three modalities are tumour-versus-normal contrasts on the same disease and are not independent of one another, so a gene with a large true effect will share direction across them for reasons that have nothing to do with GMSC paracrine signalling. That result is therefore reported as a cross-platform reproducibility and quality-control property of the transcriptional programme, measured across three disjoint patient populations and three platforms with no shared samples, and not as a line of evidence for the paracrine model.

The prognostic layer is retained as a supporting observation, not as evidence for this paper’s OSCC framing. Its pre-registered oral-cavity signature is null under internal validation and null again on external application in an independent oral-cavity cohort; the association that survives out-of-fold and externally is confined to the anatomically unrestricted pan-HNSC sensitivity cohort. Because the scope of this paper is oral-cavity disease, the honest statement is that this layer contributes little to the OSCC argument, and it is reported here for completeness and for the negative result it establishes.

The evidence-convergence alluvial panel that appeared in the previous version has been retired. Its caption described ribbons tracing each wet-lab anchor through supporting layers, whereas the code drew prior → ligand family → programme, and its subtitle asserted “4 independent priors”, which is false for one of the four. Figure 11 now carries the DepMap panel, the corrected spatial panel and the two permutation-null panels that make the spatial correction auditable.

## 4. Discussion

### 4.1. Synthesis

Using the same primary OSCC cells and the same GMSC secretome, we and our companion study report paracrine output that engages two opposing programmes: a tumour-protective apoptosis × ROS arm (companion study [13], where the wet-lab quantification is itself reported as preliminary and underpowered at n = 3 donors) and a pro-tumour proliferation × migration arm (this work). Two qualifications must travel with that sentence from the outset: First, in the present work the pro-tumour arm rests on the transcript panel alone, and that panel indicates the shift rather than establishing it: it is normalised to a single reference gene whose stability under GMSC-CM exposure was not assessed, and to amplification efficiencies that were never determined, so a global normalisation shift remains a coherent alternative explanation for part of the uniform direction (Section Constraints on Experimental Re-Validation). Second, the functional read-outs did not follow (metabolic activity was unchanged and wound closure was reduced under conditioned medium), so the two arms are opposed at the level of gene expression and apoptosis assays, not at the level of proliferation and migration measured here (Section 3.1).

These are not contradictory findings; they reflect the well-described pleiotropy of MSC-paracrine signalling. The secretome’s factor mix (TGF-β family, IGFs, FGFs, IL-6, MMPs, thrombospondins, fibronectin, collagens, chemokines) drives different downstream programmes depending on the receiving cell state, paracrine concentration and timepoint. Our framework identifies which ligands are plausible carriers of the pro-tumour programme, tests whether the programme is visible in patient tumours, asks whether it carries prognostic information, and screens catalogued compounds for signatures that oppose it. It does not translate the duality into a clinical strategy, and we withdraw the claim that it does: the two contrasts we scored are near-duplicates of one another (Section 1.4 and Section 4.5) and the companion contrast carries no significant compound-level signal, so nothing in this study supports axis-selective targeting.

### 4.2. Ligand–Receptor Geometry, and What “Convergence” Can and Cannot Mean Here

Three mechanistically distinct rationales (PPI shortest-path topology, ligand–target regulatory potential and expression-based cell–cell communication) applied to a shared, pre-registered 46-ligand candidate space, select overlapping subsets of that space at approximately the rate expected by chance (observed three-way intersection 22 ligands versus 19.38 ± 1.61 under 20,000 within-universe permutations, empirical *p* = 0.091) and do not agree on which ligands matter most (Kendall’s W = 0.312, χ^2^ = 19.68, df = 21, *p* = 0.541; W ranged 0.180–0.579 across 48 alternative scoring choices, with only 2 of 48 reaching *p* < 0.05, both using uncalibrated abundance-driven statistics). This is a statement about coverage of a curated list, not about independent convergence, and the submitted claim that “three independent prior-knowledge analyses point to the same canonical MSC-paracrine ligand set” is withdrawn. Nothing takes its place: the three-way cross-platform agreement of Section 3.3 is a reproducibility check on the transcriptional programme, not a substitute line of evidence for convergence (Section 3.12).

What survives is a description. The ligands repeatedly nominated are the canonical MSC-paracrine set—collagens 1A1/1A2/3A1, fibronectin, thrombospondins 1/2, TIMPs 1/2/3, MMPs 2/9/13, HGF, IGF1/IGFBP3, ANXA1/2, WNT5A, PTGS2, CXCL1, IL6, TGFB1/2/3, BMP2, FGF2—and the receivers named on the malignant compartment are SDC1/4, CD44, ITGA5/ITGB1 and DDR1. EGFR has been removed from that receiver list: its only spatial support, ANXA1 → EGFR, collapses from ρ = 0.79 to +0.199 (Section 3.9). Downstream, anchor-excluded GSEA recovers Hallmark EMT, G2-M Checkpoint, E2F Targets and Interferon responses together with Reactome ECM-receptor and collagen formation modules (the programme one would expect from a CAF-like paracrine input), and CollecTRI shows coordinated activation of EGR1, RUNX2, SMAD2, E2F1, FOXM1, KLF5, ELK1 and TCF7L2 with reduced activity of the anti-EMT factor KLF17 in the high-anchor stratum (Section 3.7). The defensible synthesis is that GMSC paracrine input engages stromal-receptor signalling on malignant OSCC cells and is associated with an EMT and cell-cycle programme accompanied by ECM-remodelling enzymes that would be expected to enable invasion [81]. The word “engages” is doing hypothesis-level work: none of these layers measures the interaction in our own system.

### 4.3. Cell–Cell Communication Hypotheses Examined in Independent Spatial Data

The GSE300414 GeoMx cohort (24 HPV-negative, tumour-budding HNSCC patients; 133 ROIs) [50] provides a tissue-level check on the LIANA predictions. It is a check, not the “strongest external validation” the previous version called it, for three reasons: its candidate space is LIANA’s own output; the deposit is a subgroup (all 24 patients are from the tumour-budding arm of a 43-patient series, and the 19 non-budding patients are not deposited); and the result is null at the set level.

Correctly normalised, 3 of 153 predicted pairs exceed ρ > 0.6 against a permutation background of 3.29%, binomial *p* = 0.88, with the whole ρ distribution indistinguishable from the null (Section 3.9). Three pairs (TIMP1 → CD63, THBS1 → SDC4 and THBS2 → SDC1) reach FDR < 0.05 on the nominal statistic, and TIMP1 → CD63 is stronger after correction than before; none survives permutation calibration. A THBS/TIMP–syndecan subset that is spatially consistent in a small budding-enriched cohort is a modest, honest finding and is what we now claim. The wet-lab anchor pair CXCL12 → ITGB1, which the previous version reported at ρ = 0.70, is null on normalised counts (−0.030, *p* = 0.891).

We also withdraw the statement that the independent cohort “recapitulates the LR ranking”, and we withdraw it on grounds a re-reviewer can verify. Across all 153 pairs, the LIANA rank is weakly associated with spatial correlation in the supportive direction (Spearman −0.262, *p* = 0.001; specificity rank −0.290, *p* = 3 × 10^−4^), but the association is fully attributable to transcript abundance (partial Spearman conditioning on mean GeoMx abundance of both partners: −0.059, *p* = 0.47). Highly expressed genes rank well in LIANA and correlate well in GeoMx, and that is sufficient to produce the association. On the earlier raw-count pipeline, the same 153-pair association has the opposite, anti-supportive sign (+0.284, *p* = 3.7 × 10^−4^). All three numbers are reported so that nothing here depends on which subset a reader chooses to recompute.

It is fair to ask why the foundation-model layer was withdrawn entirely while this one is retained and reported, and the distinction should be stated. The two failures are of different kinds. The foundation-model layer failed a calibration gate: it was never shown to recover known ligand–target relationships, so nothing it subsequently produced could be interpreted in either direction, and a pre-registered rule required its removal. The spatial layer needed no such gate and failed none. What did not hold here was not the instrument but the hypothesis the instrument was built to test. A corrected analysis returning a null is a result, and we report it as one; an uncalibrated instrument is not a result at all. We have therefore removed the first and retained the second—reporting its negative set-level finding, the collapse of the wet-lab anchor pair CXCL12 → ITGB1 under normalisation, and the one pair that improves. We note in the same place that the layer could never have served as independent validation of the LIANA predictions, for a structural rather than a statistical reason: its entire candidate space was read out of LIANA’s own output file.

### 4.4. Patient-Cohort Relevance and the Prognostic Layer

The defence against the post hoc circularity critique in this study is structural, not statistical: the eight anchors cannot enter the LASSO–Cox feature pool, by construction. The prognostic performance is reported below and is weaker than we previously stated; it is no longer offered as the answer to the circularity question, and demoting it costs that argument nothing.

The prognostic layer requires an explicit and uncomfortable statement, because two things we report are both true and pull in opposite directions. After restricting the models to primary tumours and refitting the Coxnet coefficients, the anchor-excluded LASSO–Cox signature separated overall survival in all three-cohort variants, and more strongly than we originally reported (apparent HR 1.575, 1.612 and 1.602 in STRICT, BROAD and PAN-HNSC; Cox *p* 1.1 × 10^−5^, 1.4 × 10^−5^ and 8.5 × 10^−11^). Those in-sample statistics, however, do not survive internal validation in the two oral-cavity cohorts. With the entire selection procedure (the Coxnet path and the 5–15-feature regularisation rule) re-executed inside every training fold, cross-validated discrimination in STRICT was 0.506 ± 0.021, indistinguishable from chance, and the out of fold hazard ratio was 1.014 (0.789–1.304, *p* = 0.911); BROAD gave 0.518 ± 0.021 and 1.198 (0.939–1.528, *p* = 0.146). Under 1000 permutations of the survival labels, the same pipeline reached Cox *p* < 0.05 in 100.0% and 99.9% of runs, and the median permuted hazard ratio (1.702 and 1.696) exceeded the observed value, giving empirical *p* = 0.809 and 0.688. We therefore state plainly that the in-sample significance of a LASSO-selected signature at these sample sizes carries no information about survival, and that our previous report of “replication across three-cohort variants” was doubly unsupported: the cohorts are strictly nested (STRICT ⊂ BROAD ⊂ PAN-HNSC; BROAD adds twelve tumours to STRICT, Jaccard 0.934), and neither oral-cavity fit survives a permutation null.

Only PAN-HNSC survives. Its cross-validated C-index was 0.568 ± 0.013, its out-of-fold hazard ratio 1.361 (1.190–1.557, *p* = 7.0 × 10^−6^), its in-sample permutation *p* = 0.001 and its out-of-fold permutation *p* = 0.005; all six of its selected features were retained in at least 64% of cross-validation folds, whereas five of STRICT’s nine were retained in fewer than half. The scope caveat must be stated in the same breath: PAN-HNSC is the cohort variant we pre-registered as a legacy sensitivity analysis precisely because it is anatomically unrestricted and includes HPV-positive disease, and STRICT—the cohort whose oral-cavity, HPV-negative composition underpins our answer to the extrapolation question raised in review—is a 33% subset of it. We do not resolve this tension by relabelling cohorts. We keep STRICT as the pre-registered primary read-out and report it as null after validation; we report PAN-HNSC as the sensitivity variant it was always labelled, in which a prognostic signal is present out of fold but cannot be attributed specifically to oral-cavity disease; and we withdraw the prognostic result from the paper’s headline claims, retaining it as a supporting observation. One further disclosure belongs here: AREG, an EGFR ligand and hence a direct network partner of an excluded anchor, is among the six features selected in PAN-HNSC, and 10.0% (56 of 562) of the retained feature pool are STRING high-confidence direct partners of an excluded anchor. Anchor exclusion removes the measured genes; it does not remove their pathways. The defence against circularity in this study is structural (the eight anchors cannot enter the feature pool) and not the size of the resulting hazard ratio.

An external cohort has now been analysed. Neither GSE30784 nor GSE37991 carries outcome data, so we went to an outcome-annotated series: GSE41613 (GPL570; 97 HPV-negative oral-cavity SCC, 51 deaths) [42], with the TCGA coefficients frozen and the analysis plan (cohort, preprocessing, endpoint, metrics and all six variants) fixed before any score was related to outcome (Section 2.2.6 and Section 3.6). The result is split, and the split is the informative part. The pre-registered primary signature does not externally validate: STRICT gives C = 0.562 (0.475–0.649), log-rank *p* = 0.301 and an adjusted HR of 1.280 per SD (0.966–1.697, *p* = 0.085)—the right direction, a non-significant trend, exactly as the internal package predicted. The PAN-HNSC sensitivity signature, the only variant that survived internal validation, does discriminate: C = 0.625 (0.530–0.708), adjusted HR 1.630 per SD (1.176–2.259, *p* = 0.0034), rising to 1.665 (1.165–2.381, *p* = 0.0052) with stage and treatment in the model; BROAD is null externally as it was internally. That the same signature separates from the same two in an independent cohort is a pre-specified prediction that could have failed and did not.

Three restraints belong with that statement, and none of them is optional. First, we do not promote PAN-HNSC to the primary result. Figure S1A designates STRICT as the pre-registered primary and PAN-HNSC as a legacy sensitivity variant; swapping them after seeing which one survives would be outcome-dependent selection, and doubly so here, since PAN-HNSC was already the variant that survived internally. Second, no risk-group claim is made: the median-split log-rank test is non-significant for every variant, PAN-HNSC included (*p* = 0.067), so the prognostic information resides in the continuous score and dichotomising discards it. Third, the scope mismatch is stated rather than buried: GSE41613 is oral-cavity and HPV-negative, matching STRICT’s scope, whereas the signature that was validated was fitted on the anatomically unrestricted, partly HPV-positive cohort—a transfer across scope, and the opposite of the arrangement one would design. We also note what this external test does not undo: the 100% false-positive rate under label permutation is a property of the selection procedure, and no selection took place here, so it neither discounts the external result nor rescues the withdrawn in-sample claims. For context rather than replication, our companion study validated its own, different signature in this same deposit and reported C = 0.57, log-rank *p* = 0.18 and a multivariable HR of 1.24 per SD (0.95–1.61, *p* = 0.11) [13]; the two signatures share neither anchor panel nor feature pool nor fitting cohort, so the useful observation is only that both papers independently find at most modest prognostic information in this cohort. At n = 97 with 51 events, the lower bound of the PAN-HNSC C-index interval is a discrimination too weak to be clinically useful, and one deposit on one platform from one centre is a single external cohort. The honest reading is detectable prognostic information in a sensitivity signature, not a usable prognostic test.

Two further consequences of the correction should be recorded rather than buried. The submitted claim that the LASSO–Cox signature “outperforms” the anchor-based z-sum signature, and that this argued against circularity, is withdrawn: the corrected z-sum reaches *p* = 2.51 × 10^−4^ in PAN-HNSC and *p* = 0.071 and 0.060 in STRICT and BROAD, and the LASSO’s apparent advantage was partly manufactured by the 13 solid-tissue normals, of which 12 fell in the LASSO HIGH stratum and all 13 in the z-sum and G2-M LOW strata. And the permutation result should be stated in its own right, because it is a general finding rather than a property of this dataset: at n ≈ 171 with a 5–15-feature LASSO–Cox selection rule, 100% of label-permuted runs reach Cox *p* < 0.05, and the median null hazard ratio exceeds the observed one. That is a cautionary result about a very widely used pipeline; it is deposited with the code, and it is the most defensible methodological contribution this revision makes.

### 4.5. Therapeutic Candidates: What the Screen Supports and What It Does Not

The submitted manuscript presented a dual-axis-selectivity score as the translational output of this work. That framing is withdrawn. Quadrant assignment was driven almost entirely by the primary axis, because no compound reaches padj < 0.05 anywhere on the companion axis (minimum padj 0.2552), and z (companion) takes only 15 distinct values across 5332 tokens: subtracting that axis removes noise.

One clarification belongs here, because the two papers can otherwise appear to disagree. The companion study nominates HSP90 inhibitors and mitoxantrone from its apoptosis–ROS axis [13]. That is not in conflict with the statement above. The companion-axis connectivity reported here is our own re-derivation from the companion’s differential-expression table, using this paper’s anchor-excluded top-100 query lists, this paper’s three LINCS libraries, and Benjamini–Hochberg correction across all 5332 tokens; it is not the procedure by which the companion’s own candidates were obtained, and nothing here should be read as an attempt (successful or otherwise) to reproduce them.

The score additionally cannot distinguish reversal from mimicry (Section 3.10). Figure 10 draws no quadrant scheme, and no axis-selectivity claim is made anywhere in this paper.

Gefitinib remains our nominated candidate, but the basis for the nomination must be stated accurately, and it is narrower than we previously implied. In a direction-resolved re-analysis of the same cached LINCS responses, gefitinib’s single Benjamini–Hochberg-significant association on the proliferation/migration contrast is the LINCS consensus *Gefitinib Down* signature intersecting the tumour-up gene list—that is, reversal geometry, which is the direction our interpretation requires (9 of 245 genes, *p* = 3.9 × 10^−6^, padj = 0.017). Three qualifications follow. First, the association is carried by seven MAGE-family cancer-testis antigens (MAGEA1, MAGEA3, MAGEA4, MAGEA6, MAGEA12, MAGEB2, MAGEC2, alongside HMGA2 and PAEP); because these lie largely in one co-regulated cluster, the independence assumption of the enrichment test is violated, and removing them takes the association from *p* = 3.9 × 10^−6^ to *p* = 0.30, against a Benjamini–Hochberg threshold of 1.1 × 10^−5^ at that rank. Second, the direction is not independently replicated: across 78 gefitinib signatures in the per-perturbation LINCS libraries the split is 25 reversal to 17 mimicry against a 51.3% baseline (binomial *p* = 0.18) and no individual signature reaches padj < 0.05, while across the EGFR-inhibitor class as a whole the strongest mimicry association (*p* = 1.4 × 10^−5^) is stronger than the strongest reversal association (*p* = 1.3 × 10^−3^). Third, no EGFR-pathway gene appears among the driver genes, and none could: EGFR is one of the eight excluded anchors, so this analysis was blind to EGFR by construction.

We should therefore be explicit about why gefitinib, and not another compound. It was not the top-scoring compound (rank 17 of 5332 at λ = 1.0, and ranks 1 and 8 of that ranking are LINCS plate barcodes admitted in error by our name parser), and it was not the top-scoring compound carrying a regulatory-approval flag—monobenzone outranks it at every value of λ, and does so after direction correction as well. Nor is the “selectivity” component doing work, for the reasons given above. Gefitinib was selected because it is the only compound in the annotated set with a coherent prior link to the biology under study: EGFR is one of our wet-lab anchors and is up-regulated in the tumour contrast, DepMap CRISPR data identify EGFR as a dependency in 69.8% (37 of 53) of oral-cavity SCC lines against 19.0% of all 1178 screened lines, the largest lineage selectivity among the twelve genes crossing the essentiality threshold (Section 3.11), PROGENy shows EGFR-pathway activity in the same direction in the high-anchor stratum (nominal *p* = 0.025, though not surviving FDR correction, padj = 0.072), and EGFR-directed therapy is established in head-and-neck oncology [82]. That is a prior-driven nomination that our screen did not contradict, not a discovery made by the screen. We also withdraw, without qualification, the claim that EGFR inhibition leaves the protective apoptotic arm intact: no experiment in this study or its companion tests addresses it, and the citation previously attached to it does not address it. Gefitinib is reported here as a hypothesis-generating candidate requiring the experimental test that reviewers 1 and 3 correctly identified as missing, and which the conclusion of the study programme prevents us from performing (Section Constraints on Experimental Re-Validation).

One consequence for the DepMap result should be stated in the same place, because it is the seam a careful reader will look for. EGFR is a genuine dependency in this lineage, and, unlike the seven Common Essential genes in the same panel, a dependency that is selective for it: median Chronos −0.677 and essentiality in 69.8% of the 53 oral-cavity SCC lines, against −0.189 and 19.0% across all 1178 screened lines (Section 3.11, Table 4). That result supports the target. It cannot support the discovery, and never could have, because the screen that nominated gefitinib was blind to EGFR. The defensible chain runs in one direction only: EGFR is up-regulated in the tumour contrast and is a genuine dependency in oral-cavity SCC lines → an EGFR inhibitor is a reasonable prior-motivated candidate for this programme → a connectivity screen that was blind to EGFR did not contradict that prior. The words “validates”, “confirms” and “orthogonal signals converge” have been removed accordingly. Two further limits belong with the datum. EGFR’s absolute effect size is mid-tier—seven genes are more strongly depleted in this panel, and what makes EGFR the informative one is selectivity rather than magnitude—and in close to a third of the oral-cavity lines (16 of 53) EGFR does not reach the essentiality threshold at all, so the dependency is real but partial.

### 4.6. Reconciliation with the Companion Study

This work and the companion study [13] may at first look like they reach opposite conclusions: GMSC paracrine output increases apoptosis and reduces ROS in the companion paper, and induces a pro-proliferative, pro-migratory transcriptional programme in the present one, in the same OSCC cell cultures. The reviewer comment asked precisely the right question—whether the two effects occur simultaneously in the same cells, whether they are temporally distinct, or whether one depends on the other—and it deserves an answer in three parts rather than a reconciling narrative.

*First, what the data show: concurrency at the read-out level.* Both arms were measured at the same 24 h timepoint on the same three primary OSCC cultures, so the two sets of read-outs are concurrent as measurements. That is the whole of what is established.

Second, what the data cannot show: concurrency in the same cells. Bulk RT-qPCR and plate-level apoptosis and MTT read-outs average over the population. A mixture in which one subpopulation undergoes apoptosis while another proliferates would produce exactly the observed pattern, and so would a uniform population in which both programmes are engaged. Population heterogeneity is, in our view, the more probable explanation—it would also help account for the tension between an apoptotic response in the companion arm and unchanged metabolic activity here—but we label that an inference, not a result. The experiment that would settle it is a single-cell co-stain, Annexin V together with an incorporation-based proliferation marker such as EdU, on the same treated population.

Third, dependency is untested. Nothing in either paper addresses whether either arm requires the other, or whether blocking one alters the other. We therefore state that the two arms co-occur at the level of the assays performed, that concurrency within individual cells is not established, and that dependency is unknown.

Two provenance statements from the earlier text are also qualified here. The two papers share the cell systems and the experimental design, and the wet-lab work was performed in the same laboratory on the same three primary OSCC cultures and the same single GMSC donor preparation [13]; we no longer assert this as though it were independent corroboration of either arm, and we no longer describe it as making “axis-selective targeting feasible”. At the computational level the two arms are not separable at all: the two differential-expression contrasts—the same tumour-versus-normal contrast computed on two heavily overlapping definitions of the same TCGA-HNSC oral-cavity cohort (Section 2.2.2)—correlate at Spearman 0.949 and Pearson 0.932 over 27,532 shared genes (94.49% sign concordance; 0.9923 and 99.80% among the 9885 jointly significant genes), and their anchor-excluded top-100 up lists share 74 of 100 genes. The single region in which the two runs differ is a high-variance cancer-testis antigen family handled differently by two DESeq2 runs of the same contrast (six of seven MAGE genes carry padj = NaN in the companion run at near-identical baseMean), and that region is exactly the one that generated the entire gefitinib nomination (Section 4.5). L12 must not be read anywhere as evidence that the two arms are separable.

#### Constraints on Experimental Re-Validation

This subsection states once, openly, a constraint that bears on five of the requests made in review; a sixth (the donor and specimen counts of reviewer 1’s major 3) is now answered from the published companion study on the same cell systems [13] and is dealt with in the first bullet below. The study programme that generated the experimental data reported here has concluded. No further biological material, no further funding and no further laboratory records are available to the authors; part of the work, including the flow cytometry, was outsourced, and the outsourced facility retained neither the raw files nor the per-donor clinical metadata [13]. Several reasonable requests therefore cannot be answered with data, and we set out what could not be performed together with the interpretive consequence of each, rather than distributing partial answers across the response. Every limitation below is carried into Section 4.7.

- Donor and specimen counts, and biological versus technical replication—answered, and our earlier position corrected. At submission, we stated that these counts could not be retrieved. They are documented in the companion study on the same cell systems, now published [13]: three independent patient-derived primary OSCC cultures (one tumour per donor; n = 3 biological replicates), each assayed in technical triplicate, with the donor as the unit of analysis, and a single GMSC donor preparation applied across all three. Consequence: n = 3 samples from three OSCC donors rather than one culture three times, which is a stronger design than we previously described; but GMSC donor-to-donor secretome variation is not sampled at all, and n = 3 still powers only large effects. What remains unavailable is the per-donor clinical metadata (stage, site, sex and age), which the outsourced facility did not retain [13].
- MIQE reaction and cycling details: Reaction composition and volume, cycling conditions, instrument model, and melt-curve and no-template-control records are not recoverable [24]. Consequence: the RT-qPCR cannot be replicated exactly from this manuscript; it can be replicated in design but not in parameters.
- Primer amplification efficiency: Standard curves were not run and cannot now be run. This is the substantive point behind the request, and we concede it rather than deflect it: the 2^−ΔΔCq^ method assumes comparable amplification efficiencies between target and reference, and that assumption was verified only in silico (by primer specificity checking) and never experimentally. Consequence: the fold-changes are relative and internally consistent; they are not absolutely calibrated, and the magnitude of any individual fold-change should not be over-interpreted. The direction and the significance tier, which this paper uses, are unaffected by a constant efficiency offset but would be affected by a large target-versus-reference efficiency mismatch.
- GAPDH stability: GAPDH was used as the single reference gene, and its stability across GMSC-CM exposure was not formally assessed. Consequence: if GAPDH itself shifted under treatment, every fold-change would shift with it. Because all eight targets move in the same direction in all three arms, a reference shift is a coherent alternative explanation for part of the effect size, though not for the relative ordering across genes. A multi-reference design with a stability statistic is the correct remedy and is named in Section 4.8.
- Cell-line authentication (STR profiling) and HPV typing: The primary OSCC cultures were not STR-profiled, and their HPV status was not determined; neither can now be done. Epithelial and stromal purity was likewise not assessed by immunophenotyping, so stromal contamination cannot be formally excluded [13]. Consequence: identity and cross-contamination cannot be excluded, and the correspondence between our HPV-untyped primary culture and the HPV-negative-restricted computational cohorts rests on epidemiology rather than on measurement—HPV-driven disease in the head and neck is largely confined to the oropharynx, and oral-cavity SCC in tobacco- and betel-quid-exposed populations is predominantly HPV-negative [4]—which is an assumption, not a verification.
- Mycoplasma testing: No dedicated mycoplasma testing was performed [13], and none can now be performed. Consequence: an unrecognised contaminant could alter proliferation and migration behaviour; this is a general caveat on all functional read-outs.
- Serum-matched functional repeat: The conditioned-medium arms are serum-depleted relative to the control, while the Transwell arm is not (Section 2.1.4); the degree of serum depletion is itself uncertain, with the companion study describing a serum-free 48 h conditioning step [13] against the serum-reduced 24 h step recorded here. The plate that would test whether this explains the reduced wound closure cannot be run. Consequence: the serum confounds stated in Section 3.1 remain an untested candidate explanation, and the functional result stands as reported (closure reduced under conditioned-medium, unchanged under the serum-matched arm) rather than being explained away.
- Numeric correction of Figure 2, and the scope of the companion’s availability statement. The absorbance values, wound areas and Cq values underlying Figure 2 are not available to us, so no numeric quantity in Figure 2 can be recomputed or corrected. One point of precision is owed here, because the companion study’s Data Availability Statement offers its raw wet-lab data (flow-cytometry files and qPCR Ct values) from the corresponding author on reasonable request [13]. That statement covers the companion’s own experiments and therefore its six-gene apoptosis–ROS panel (BAX, BCL2, CASP3, CASP9, NOX1, GPX1). It does not cover the eight-gene proliferation/migration panel reported here, whose Ct file was not retained, nor the metabolic-activity absorbances or wound areas of our Figure 2, which were generated in the same period and are likewise not available to us. The two panels share only the GAPDH reference assay. We state this rather than leave the two availability statements to be read against each other. Consequence: this manuscript reports only the direction and the significance tier that the figure’s own annotations licence, and prints no mean or standard error for any wet-lab measurement.
- Catalogue and lot numbers, and the flow-cytometry schedule: Manufacturer names and corporate locations are public and are supplied in Section 2.1 for every item whose manufacturer is on the record; no supplier is recorded for trypsin–EDTA, PBS, the GMSC isolation enzymes, the 0.22 µm filter, MTT reagent or dimethyl sulfoxide, and none has been supplied. Catalogue numbers, lot numbers, and the microplate reader, microscope and thermocycler models cannot be retrieved. Separately, the antibody clones and isotype controls, the cytometer and the analysis software used for the flow run shown in Figure 1 are not recorded; the companion publishes a full flow schedule on the same cell system [13], but for a demonstrably different acquisition (Section 2.1.3), so it has not been adopted here. Consequence: reagent-level reproducibility is partial, and Figure 1 acquisition cannot be repeated to specification.
- Any test of gefitinib: Gefitinib was never applied to our cells, with or without GMSC-CM, and cannot now be. Consequence: the drug-repurposing result is a prediction. It is presented as such throughout, and Section 4.8 specifies the experiment that would test it.

We recognise that this list is long and that some readers will regard it as disqualifying for the translational claims originally made. That is why those claims have been withdrawn rather than hedged. What the constraint does not touch is the computational programme: every correction described above—the survival refit and its validation package, the spatial re-run, the DepMap panel correction, the direction-resolved connectivity analysis, the withdrawal of the foundation-model layer, and the version, citation and terminology repairs—was performed on public data and deposited code, and none of it depended on further laboratory work.

### 4.7. Limitations

The limitations below are ordered from the most consequential downwards, and each is stated with the conclusion it bounds.

(i) The wet-lab foundation is narrow, and thirteen computational layers do not widen it. The experimental evidence comes from n = 3 biological replicates (three patient-derived OSCC cultures, one tumour per donor) against a single GMSC donor preparation, at a single 24 h timepoint, with no cell-line authentication, no HPV typing and no protein- or phospho-level measurement in our own system; between-GMSC-donor variability is not sampled at all [13], and at n = 3 donors the wet-lab arm is statistically underpowered and is reported throughout as preliminary. Only the transcript panel supports the pro-tumour direction, and it does so as an indication rather than a demonstration: it is normalised to a single reference gene of unassessed stability under GMSC-CM, with target-versus-reference amplification efficiencies never determined, so a global normalisation shift remains a coherent alternative explanation for part of the uniform direction (Section Constraints on Experimental Re-Validation). The metabolic-activity assay is null, and the migration assay runs against it (Section 3.1). All downstream computation inherits this, and the constraints in Section Constraints on Experimental Re-Validation mean it cannot be addressed here.
(ii) The foundation-model layer has been withdrawn, and the reason should be stated plainly. The submitted manuscript reported that a Geneformer-based in silico perturbation “reached the pre-registered passing criterion on two of four ligands”. It did not. Our pre-registered criterion was a signed Spearman ρ ≥ 0.4 and *p* < 0.05 on held-out canonical ligand → target pairs. The calibration returned TGFB1 ρ = +0.378 (*p* = 0.356, n = 8), IL6 ρ = −0.612 (*p* = 0.144, n = 7), and undefined values for IGF1 and TNF—undefined by construction, because their curated down-sets are empty, not because those ligands were target-poor as the supplementary caption stated. Under the pre-registered criterion, 0 of 4 ligands pass; at best 1 of 4 passes on unsigned magnitude, and the one ligand exceeding the magnitude threshold does so in the direction opposite to the curated ground truth. Three further facts compound this. The code tested median(|ρ|) ≥ 0.4 with no *p*-value test at all; the words “absolute” and “median” first appear in a decision recorded after the result was seen, so the absolute value was introduced post hoc; and the file on which the recorded “pass” rests is a hard-coded decision string emitted by a script containing no calibration code. The pre-registered fallback (demotion to a supplementary technical demonstration) was triggered and was not applied. We have therefore removed the layer, its figure and its supplementary figure entirely rather than demoting it, since the outputs would carry no evidential weight in either position. The scripts, outputs and the hard-coded decision file are retained under a deprecated directory with a dated decision-log entry and are deposited with the rest of the code, because deleting the artefacts while a deposited decision log still claimed a pass would be the worst available outcome. The layer count is correspondingly 13, not 14, and the numbering gap at L11 is retained so that deposited outputs remain addressable.
(iii) The prognostic layer does not survive validation in the cohorts the paper’s framing depends on. The pre-registered oral-cavity signature is null internally and does not validate externally either (GSE41613: C = 0.562, log-rank *p* = 0.301, adjusted HR 1.280 per SD, *p* = 0.085); only the pan-HNSC sensitivity variant discriminates externally, and it does so out of its own anatomic and HPV scope. The external arm is a single cohort of 97 patients and 51 events from one centre on one platform, on an all-cause rather than disease-specific endpoint, with a coarse adjustment set (banded age, two-level stage, treatment modality; no grade, margin or smoking); no median-split risk-group claim is supported for any signature (log-rank *p* ≥ 0.067 throughout). The three internal cohorts are strictly nested; LASSO–Cox optimism at these sample sizes is severe (100% of label-permuted runs reach Cox *p* < 0.05, a property of the selection procedure and not of the frozen external test); features were not standardised before the L1 penalty; and the pre-registered stage covariate was never implemented internally, so the internal multivariable adjustment covers age and sex only (Section 2.2.6, Section 3.6 and Section 4.4).
(iv) The spatial layer is not an independent validation and shows no set-level enrichment. Its candidate space is L10’s output; the deposit provides 24 patients, all from the tumour-budding subgroup, rather than the 43 patients of the published series; 3 of 153 pairs exceed ρ > 0.6 against a 3.29% background (*p* = 0.88) and none survives permutation-calibrated FDR; the parametric Spearman *p* is ~34× anti-conservative in this design; AOI-level correlation across 24 patients has low power; and nuclei-count and surface-area QC fields were not deposited, so segment QC is partial (Section 3.9).
(v) The essentiality analysis is restricted to lines whose Oncotree lineage is head and neck. The primary analysis is now restricted to the 53 oral-cavity SCC lines, with the 77 head-and-neck lines as a sensitivity stratum. The correction is conservative (ρ = 0.9971 between published and corrected medians) and strengthens the EGFR result, but it was an error in the deposited analysis and is reported as one (Section 3.11). A second limit bounds what the layer can carry even when correctly stratified: seven of the twelve genes crossing the essentiality threshold are DepMap Common Essential genes, required by almost every screened line, so their appearance in an oral-cavity ranking is uninformative about oral-cavity SCC. EGFR shows by some margin the largest lineage selectivity of the twelve (oral-cavity median Chronos −0.677 against −0.189 across all screened lines; essential in 69.8% of oral-cavity lines against 19.0% of all lines), and the layer should be read as evidence about that one gene (Table 4).
(vi) Anatomic and platform scope: The CPTAC analysis is HNSCC-wide and not oral-cavity restricted, and PAN-HNSC (the only cohort in which the prognostic signal survives) is anatomically unrestricted and includes HPV-positive disease. These are the same caveat and are disclosed together: two of the results this paper leans on are HNSCC-level, while its clinical framing is oral-cavity-level. An oral-cavity-restricted CPTAC sensitivity analysis is a named follow-up; it is not run here because the pooled CPTAC output is the source for Figure 3C and Figure 4A–C and for the ρ = 0.52 and VEGFA results, and overwriting it would invalidate results reported elsewhere in this paper.
(vii) Prior-knowledge dependence: The NicheNet regulatory-potential prior includes contributions from earlier MSC-paracrine studies. MODE-A (anchor-excluded) is reported as primary and MODE-B as sensitivity, but residual prior bias cannot be excluded, and the 46-ligand candidate space was itself assembled from that literature (Section 2.2.12).
(viii) The matrix decomposition is not the method originally intended. Tensor-cell2cell could not be run, and a truncated SVD was substituted; it captures the dominant variance mode, but component 1 (91.1%) is an uncentred scale term rather than a latent communication pattern, so only components 2–5 (2.7%, 2.4%, 2.1%, 1.7%) are candidates for interpretation, and none is large (Section 3.8).
(ix) Meta-analysis breadth: GSE25099 was excluded from the L2 meta-analysis because its platform (GPL5175) lacked a usable probe-to-gene mapping in our cache. A two-cohort meta-analysis remains informative (Spearman ρ = +0.738 against TCGA across 14,760 shared genes) but has less robustness to cohort-specific artefacts than a three-cohort one would. The companion study included GSE25099 in its own three-cohort meta-analysis [13], so the exclusion here reflects the state of our probe-annotation cache at the time of analysis rather than an inherent property of the series. Re-acquiring the GPL5175 mapping and adding the cohort is a straightforward extension; we have not performed it here because the L2 output feeds Figure 3B,C and Figure 4D–F and Section 3.3, and regenerating it would change quantities that are answered elsewhere in this response.
(x) Companion-study dependency: The companion study is published and open-access [13], and its code and data are public (github.com/SPatil555/oscc-gmsc-apoptosis-ros-pipelinev1.0.0, accessed on 21 May 2026; Zenodo 10.5281/zenodo.20740146). The dependency is narrow in any case: the L12 analysis consumes exactly one companion file, byte-identical to a file in that public archive, and the derived 200-gene list together with the six cached companion-axis Enrichr responses are deposited with this paper’s code, so the layer reproduces offline from either deposit.

### 4.8. Future Directions

The experiments that would convert the present predictions into results are specified here so that they are named rather than gestured at, and we note explicitly that we are not in a position to perform them (Section Constraints on Experimental Re-Validation).

(a) The gefitinib test: An in vitro treatment of primary OSCC cells was performed with gefitinib, with and without GMSC-CM, with read-outs on the same metabolic activity, migration, and eight-gene RT-qPCR panel used here, using a dose–response and a vehicle arm. This is the experiment both reviewer 1 and reviewer 3 identified as missing; it is the only experiment that can test the paper’s translational prediction, and orthotopic xenograft work co-implanting GMSCs would follow only if it were positive.
(b) *A serum-matched functional repeat*: A conditioned-medium plate in which the control is matched to the conditioning medium’s serum content, alongside an incorporation-based proliferation read-out (EdU or BrdU) to replace the metabolic-activity proxy. This would convert an explained-away negative into a real result in either direction, and it needs no new reagent class.
(c) A concurrency test: An Annexin V/EdU co-stain on the same treated population, to establish whether the apoptotic and proliferative arms are engaged in the same cells or in different subpopulations (Section 4.6).
(d) A longitudinal time-course (3, 12, 24, 48 and 72 h) to dissect the temporal relationship between the protective and pro-tumour arms, which a single 24 h timepoint cannot resolve.
(e) Reporting-standard remediation at the bench: primer-efficiency standard curves for all nine assays, a multi-reference-gene stability assessment, STR profiling and HPV typing of the primary preparation, and full MIQE documentation.
(f) *Further external prognostic validation*: The first external test has now been performed in GSE41613 (Section 2.2.6 and Section 3.6): the pre-registered oral-cavity signature did not validate, and the pan-HNSC sensitivity signature did, on the continuous score only. What remains is a second, independent oral-cavity HPV-negative cohort—ideally with grade, margin status and smoking recorded, and large enough to test a dichotomised risk-group claim that 97 patients cannot—together with a signature refitted within oral-cavity scope, since the variant that validated was fitted on anatomically unrestricted disease. Only that can determine whether the pan-HNSC signal belongs to the disease this paper is about.

## 5. Conclusions

The evidence assembled here and in our companion study indicates programme-dependent dual effects of gingival mesenchymal stem cell paracrine signalling on oral squamous cell carcinoma: protective in the apoptosis × ROS arm reported in our companion study [13], and pro-tumour in the proliferation × migration transcriptional programme reported here. In the present work, that pro-tumour arm is indicated transcriptionally and not further (all eight panel genes raised in all three treatment arms, 16 of 24 comparisons significant), while the functional read-outs did not follow, and we report that discrepancy rather than resolving it by assertion. One limit must be read in the same breath as that claim. The transcript panel is normalised to a single reference gene whose stability under GMSC-CM exposure was not assessed, and target-versus-reference amplification efficiencies were never determined, so a global normalisation shift remains a coherent alternative explanation for part of the uniform direction across the eight genes, though not for their relative ordering (Section Constraints on Experimental Re-Validation). The panel is therefore consistent with a coordinated pro-tumour transcriptional shift; it does not establish one.

The framework combines wet-lab perturbation with cross-cohort transcriptomic prioritisation, protein-level integration, ligand decoding, anchor-excluded network and gene-set analysis, single-cell and spatial cell–cell communication inference, patient-cohort survival modelling with full internal validation and an external test in an independent cohort, CRISPR dependency cross-referencing and a direction-resolved drug-repurposing screen, under explicit anchor exclusion whose scope and residual leakage are quantified. Its quality control across platforms is strong—of 1358 genes significant in TCGA RNA-seq, a two-cohort GEO meta-analysis and CPTAC proteomics, 89.8% share direction in all three against 25% expected by chance, across three disjoint patient populations and three platforms—but that is a reproducibility property of the transcriptional programme and not evidence for the paracrine model, because all three modalities are non-independent tumour-versus-normal contrasts on the same disease. Its most transferable result is methodological: at n ≈ 171, a 5–15-feature LASSO–Cox selection rule reaches in-sample Cox *p* < 0.05 under 100% of permuted survival labels.

The study describes candidate ligand–receptor routes for GMSC-driven pro-tumour transcriptional signalling and nominates gefitinib as a hypothesis-generating candidate that has not been tested in our system and whose computational support rests on a single gene-set association driven by one co-regulated gene family. The design addresses circularity by construction, through the exclusion of the measured genes from the analyses that could rediscover them; mechanism and clinical translatability remain open, and the experiments that would close them are specified in Section 4.8.

## Figures and Tables

**Table 1:** RT-qPCR primer sequences for the eight-gene OSCC panel, with GAPDH as the reference gene for 2^−ΔΔCq^ normalisation. The panel covers the canonical EMT, ECM-remodelling, proliferation and angiogenesis programmes implicated in OSCC progression [14,15,16,17,18]. Amplicon lengths and transcript accessions were determined by in silico PCR of each pair against every human RefSeq transcript of the target gene; the accession shown is the MANE Select or canonical transcript, and lengths (75–226 bp) are the product lengths it yields. GAPDH is the one assay whose product length differs between transcript variants (172 bp and 226 bp); all others give a single length across every amplified variant. Specificity was confirmed by BLAST against human RefSeq RNA; every primer’s best alignment is to its intended gene, and no pair yields a product in any other transcript. Amplification efficiencies were not determined from standard curves, so 2^−ΔΔCq^ values are relative and direction-bearing rather than absolutely calibrated (Section 2.1.8).

Gene	Forward (5′ → 3′)	Reverse (5′ → 3′)	RefSeq Transcript	Amplicon (bp)
*GAPDH*	GAAGGTGAAGGTCGGAGTC	GAAGATGGTGATGGGATTTC	NM_002046.7	226
*VEGFA*	AGGGCAGAATCATCACGAAGT	AGGGTCTCGATTGGATGGCA	NM_001025366.3	75
*TGFB1*	CTAATGGTGGAAACCCACAACG	TATCGCCAGGAATTGTTGCTG	NM_000660.7	209
*MMP9*	TGTACCGCTATGGTTACACTCG	GGCAGGGACAGTTGCTTCT	NM_004994.3	97
*CXCL12*	ATTCTCAACACTCCAAACTGTGC	ACTTTAGCTTCGGGTCAATGC	NM_000609.7	88
*CCND1*	GCTGCGAAGTGGAAACCATC	CCTCCTTCTGCACACATTTGAA	NM_053056.3	135
*PCNA*	CCTGCTGGGATATTAGCTCCA	CAGCGGTAGGTGTCGAAGC	NM_002592.2	109
*MYC*	GTCAAGAGGCGAACACACAAC	TTGGACGGACAGGATGTATGC	NM_002467.6	162
*EGFR*	AGGCACGAGTAACAAGCTCAC	ATGAGGACATAACCAGCCACC	NM_005228.5	177

## Data Availability

All analysis code, environment lock-files, cached third-party API responses, the curated GMSC secretome, the anchor-exclusion audit script with its log, and all derived result tables are publicly available. Code and derived outputs are deposited at https://github.com/SPatil555/oscc-gmsc-paracrine-duality-pipeline (accessed on 21 May 2026) and archived with a version-of-record DOI at Zenodo (doi:10.5281/zenodo.21787752), which also holds the four large derived matrices that exceed the repository size policy. The deposit contains both the as-submitted and the corrected result tables, so every correction reported in this revision can be re-derived and checked. Three categories of input are not redistributed: the raw sequencing and array downloads (≈ 4.2 GB), which are obtainable from their primary archives under the version pins below; the single-cell AnnData intermediates (≈ 1.1 GB), which are fully regenerated by scripts/07b_reparse_scrna.py and scripts/16_scrna_processing.py from GSE103322; and the Enrichr gene-set matrix-transposed files together with the CPTAC abundance matrix, whose licences restrict redistribution—the KEGG-derived library in particular. Each is re-obtainable by the exact call recorded in REFERENCE_PINS.md. Public datasets are listed in Section 2.2.2. The pinned versions are: TCGA-HNSC via GDC Data Release 45.0 (4 December 2025); STRING v12.0 (combined score ≥ 700; 173,038 edges, 19,699 nodes); the Enrichr gene-set libraries MSigDB_Hallmark_2020, KEGG_2021_Human, Reactome_Pathways_2024 and GO_Biological_Process_2023, fetch date 2026-05-21; CPTAC HNSCC via PNNL cptac 1.5.14, fetch date 2026-05-21; DepMap Public 24Q4 (CRISPRGeneEffect.csv, Model.csv; figshare article 27993248); GSE300414 NanoString GeoMx Whole Transcriptome Atlas with probe-kit file Hs_R_NGS_WTA_v1.0.pkc; single-cell GSE103322; GEO bulk OSCC GSE30784 (GPL570) and GSE37991 (GPL6883); the external prognostic validation cohort GSE41613 (GPL570; series matrix downloaded 2026-07-27, md5 ad645ea2d7fac58a141cc1e9756ca0d5, cached under data/raw/geo/GSE41613/with download_provenance.json); the NicheNet NSGA-II ligand–target prior v2 (Zenodo); LINCS L1000 chemical-perturbation libraries queried through the Enrichr endpoint, all responses cached as dated JSON; DGIdb v5 via its GraphQL endpoint, responses cached as dated JSON; and an OmniPath snapshot of 25 July 2026 for the PROGENy (top = 500; 6463 rows, 14 pathways) and CollecTRI (42,990 rows, 1185 transcription factors) priors, with SHA-256 checksums recorded in data/raw/omnipath/OMNIPATH_SNAPSHOT.json. The global random seed is SEED = 20260521. The single input derived from the companion study (its oral-cavity differential-expression table) is byte-identical to a file in that study’s public repository and Zenodo archive; the 200-gene list derived from it, and the six cached Enrichr responses computed from that list, are deposited here as well. The companion is now published (doi:10.3390/ijms27146480), and its archive is public so that the file can be obtained from either deposit, and the dual-contrast layer can be reproduced offline.

## Supplementary Materials

The following supporting information can be downloaded at: https://www.mdpi.com/article/10.3390/cells15171538/s1. Figure S1: Cohort definitions for Layers L1–L3. Figure S2: Per-anchor random-effects meta-analysis forest plots. Figure S3: Ligand–receptor inference and singular-value decomposition detail (Layer L10/L10b). Figure S4: Top 50 compounds by Layer L12 score at λ = 1.0 with resolved reversal/mimicry geometry. Figure S5: CRISPR essentiality detail on the corrected cell-line panel (Layer L14). Figure S6: Sensitivity analyses. Figure S7: Internal and external validation of the prognostic layer (Layer L7). Table S1: RT-qPCR primer schedule. Table S2: Public-cohort definitions and sample sizes. Table S3: Curated GMSC secretome v1.0 with PubMed provenance. Table S4: STRING shortest-path output. Table S5: Ligand–receptor pairs and SVD component loadings. Table S6: Direction-resolved drug-repurposing shortlist, top 50, with DGIdb annotations. Table S7: CRISPR essentiality per gene. Table S8: Gene sets dropped after anchor exclusion. Table S9: Internal and external validation of the prognostic layer.

## Abbreviations

The following abbreviations are used in this manuscript:

Abbreviation	Definition
ANOVA	Analysis of variance
AOI	Area of illumination (NanoString GeoMx nomenclature)
ATC	Anatomical Therapeutic Chemical classification
BH	Benjamini–Hochberg
CAF	Cancer-associated fibroblast
cDNA	Complementary DNA
CI	Confidence interval
CPTAC	Clinical Proteomic Tumour Analysis Consortium
CRISPR	Clustered regularly interspaced short palindromic repeats
DCC	Digital count conversion (NanoString GeoMx count file format)
DE	Differential expression
DepMap	Cancer Dependency Map
DGIdb	Drug–Gene Interaction Database
DMEM	Dulbecco’s Modified Eagle Medium
ECM	Extracellular matrix
EGFR	Epidermal growth factor receptor
EMT	Epithelial–mesenchymal transition
FBS	Foetal bovine serum
FDR	False discovery rate
GDC	Genomic Data Commons
GEO	Gene Expression Omnibus
GMSC	Gingival mesenchymal stem cell
GMSC-CM	Gingival mesenchymal stem cell–conditioned medium
GO-BP	Gene Ontology Biological Process
GSE	GEO Series accession
GSEA	Gene-set enrichment analysis
HNSCC	Head-and-neck squamous cell carcinoma
HPV	Human papillomavirus
HR	Hazard ratio
ISCT	International Society for Cellular Therapy
KEGG	Kyoto Encyclopedia of Genes and Genomes
LASSO	Least absolute shrinkage and selection operator
LIANA	Ligand–receptor analysis framework
LINCS	Library of Integrated Network-Based Cellular Signatures
LR	Ligand–receptor
MIQE	Minimum Information for Publication of Quantitative Real-Time PCR Experiments
mRNA	Messenger RNA
MSC	Mesenchymal stem cell
MSigDB	Molecular Signatures Database
MTT	3-(4,5-dimethylthiazol-2-yl)-2,5-diphenyltetrazolium bromide
NES	Normalised enrichment score
NSGA-II	Non-dominated sorting genetic algorithm II
OSCC	Oral squamous cell carcinoma
PBS	Phosphate-buffered saline
PPI	Protein–protein interaction
PROGENy	Pathway RespOnsive GENes
QC	Quality control
REML	Restricted maximum likelihood
RNA-seq	RNA sequencing
ROI	Region of interest
ROS	Reactive oxygen species
RT-qPCR	Reverse-transcription quantitative polymerase chain reaction
SCC	Squamous cell carcinoma
SD	Standard deviation
STR	Short tandem repeat
STRING	Search Tool for the Retrieval of Interacting Genes/Proteins
SVD	Singular value decomposition
TCGA	The Cancer Genome Atlas
TCGA-HNSC	The Cancer Genome Atlas—head-and-neck squamous cell carcinoma
TF	Transcription factor
ULM	Univariate linear model
UMAP	Uniform manifold approximation and projection
VST	Variance-stabilising transformation
WTA	Whole Transcriptome Atlas
