# Supplementary material for: The Dual Face of Gingival Mesenchymal Stem Cell Paracrine Signalling in Oral Squamous Cell Carcinoma: A Pro-Tumour Transcriptional Programme and a Hypothesis-Generating Drug-Repurposing Screen
Source: Cells. 2026 Aug 26;15(17):1538. doi: 10.3390/cells15171538 (PMC13565543; doi:10.3390/cells15171538)
Supplement: Supplementary file 1 [file cells-15-01538-s001.zip › 04_SUPPLEMENTARY_FIGURE_CAPTIONS.pdf]

Supplementary figure captions

MDPI back-matter element (insert into the manuscript)

, although

Figures S1–S7 and Tables S1–S8 were cited throughout the text. The block below is the MDPI back-matter element and belongs in the manuscript immediately after the Informed Consent Statement and before the Data Availability Statement.

Supplementary Materials: The following supporting information can be downloaded at: [MDPI-assigned URL]. **Figure S1:** Cohort definitions for Layers L1–L3. **Figure S2:** Per-anchor random-effects meta-analysis forest plots. **Figure S3:** Ligand–receptor inference and singular-value decomposition detail (Layer L10/L10b). **Figure S4:** Top 50 compounds by Layer L12 score at $\lambda = 1.0$ with resolved reversal/mimicry geometry. **Figure S5:** CRISPR essentiality detail on the corrected cell-line panel (Layer L14). **Figure S6:** Sensitivity analyses. **Figure S7:** Internal and external validation of the prognostic layer (Layer L7). **Table S1:** RT-qPCR primer schedule. **Table S2:** Public-cohort definitions and sample sizes. **Table S3:** Curated GMSC secretome v1.0 with PubMed provenance. **Table S4:** STRING shortest-path output. **Table S5:** Ligand–receptor pairs and SVD component loadings. **Table S6:** Direction-resolved drug-repurposing shortlist, top 50, with DGIdb annotations. **Table S7:** CRISPR essentiality per gene. **Table S8:** Gene-sets dropped after anchor exclusion. **Table S9:** Internal and external validation of the prognostic layer.

Figure S1. Cohort definitions for Layers L1–L3

(A) TCGA-HNSC cohort variants. STRICT — HPV-negative oral cavity (oral tongue, floor of mouth, buccal mucosa; ICD-10 C00, C02–C06, excluding C01, C05.1, C07–C10, C13, C32); pre-registered primary; n = 171 tumour / 13 solid-tissue normal. BROAD — STRICT plus HPV-unknown oral cavity; n = 183 tumour / 13 normal. PAN_HNSC — all HNSC anatomic sub-sites; legacy proxy, retained as a sensitivity variant; n = 518 tumour / 44 normal. The differential-expression analysis (L1) legitimately uses both arms; the survival models (L7) use primary tumours only. Following the correction described in Section 2.2.6, the survival cohorts are restricted to GDC sample type 01 (Primary Tumor) before any signature scoring or model fitting, giving n = 171 (STRICT), 181 (BROAD) and 512 (PAN_HNSC) patients entering the models. Two primary tumours in BROAD and six in PAN_HNSC carry no usable overall-survival record and are excluded in addition to the normals. The solid-tissue normals that had been carried into the submitted models were patient-matched: 13 of 13 in STRICT and BROAD, and 43 of 44 in PAN_HNSC, duplicated a patient already represented by a tumour in the same cohort. The three variants are strictly nested (STRICT $\subset$ BROAD $\subset$ PAN_HNSC; Jaccard on primary tumours 0.934, 0.353 and 0.330), so agreement between them is not independent replication. (B) Independent GEO cohorts for the L2 random-effects meta-analysis. GSE30784 (Affymetrix HG-U133 Plus 2.0; 167 tumour / 45 normal) and GSE37991 (GPL6883 Illumina HumanRef-8 v3.0, as recorded in the series matrix — the submitted caption gave HumanWG-6 v3.0; 40 tumour / 40 normal). GSE25099 was excluded after acquisition because its platform (GPL5175) lacked a usable probe-to-gene mapping in our cache. (C) CPTAC HNSCC proteomic subset for L3. 116 tumour / 66 normal-adjacent samples; 12,223 quantified protein rows collapsing to 11,581 gene symbols (642 duplicate symbols resolved on maximum sample coverage, tie-broken

on adjusted p then row order); all eight wet-lab anchors retained. None of the eight anchors is duplicated in any of the three tables, so the collapse rule cannot affect the anchor results; three-way concordance is stable at 88.7–89.8 % across nine alternative collapse rules.

Figure S2. Per-anchor random-effects meta-analysis forest plots (Layer L2)

One panel per anchor gene: VEGFA (S2A), TGFB1 (S2B), MMP9 (S2C), CXCL12 (S2D), CCND1 (S2E), PCNA (S2F), MYC (S2G), EGFR (S2H). Squares = per-cohort $\log_2$ fold change ± 95 % CI, marker size proportional to inverse-variance weight; red diamond = REML pooled estimate. Each panel reports the pooled $\log_2$ fold change, the pooled p-value and Higgins I^2 in an upper-left stat box.

Heterogeneity and cross-modality discordance — revised interpretation. MYC (S2G) shows the highest between-cohort heterogeneity (I^2 = 99 %). We previously offered this heterogeneity as being consistent with post-transcriptional regulation of MYC; we now regard that reading as one possibility among several and not the most parsimonious, because I^2 of this magnitude is at least as consistent with platform and cohort effects between a two-colour-era Affymetrix array and an Illumina bead array as it is with any biological mechanism, and because MYC is non-significant in both TCGA (padj = 0.78) and the GEO meta-analysis (padj = 0.50) and significant only at the protein level — so there is no established transcript direction from which its protein-level behaviour could be discordant. The heterogeneity is reported as an observation, not as corroboration of a mechanism this study did not test.

CCND1 (S2E) is the anchor with a genuine cross-cohort contradiction, and it was not remarked on in the previous version. CCND1 is significantly down in the GEO meta-analysis (pooled $\log_2$FC = −0.313, padj = 2.11 × 10^{-3}), up but not significant in TCGA (+0.706, padj = 0.082) and up and significant at the CPTAC protein level (+0.264, padj = 4.68 × 10^{-3}). A significant disagreement in direction between two independent patient cohorts is a stronger form of discordance than anything observed for MYC, and both are now reported in Section 3.3.

Figure S3. Ligand–receptor inference and SVD detail (Layers L10, L10b)

(A) Top 50 Fibroblast → Malignant ligand–receptor pairs by LIANA consensus aggregated `lrscore` on GSE103322. Every significant pair in this atlas shares the same source–target edge type (Fibroblast → Malignant), disclosed in the subtitle to avoid a monotone colour legend. Of the 841 pairs returned, 153 have a ligand present in the curated GMSC secretome; that 153-pair set is the universe carried forward to the spatial layer. (B) Component-loading heatmap from the singular-value decomposition. Terminology corrected: no tensor was constructed and Tensor-cell2cell was not run. The `cell2cell` v0.8.4 API was incompatible with our environment, as recorded in the Decision Log, and a singular-value decomposition of the 12 patient × 1,476 ligand–receptor score matrix was substituted; five components were retained. Panel B shows the highest-`|loading|` ligand–receptor pairs per component (union across components), on a diverging blue (negative) / red (positive) scale with the `|loading|` range disclosed on the colourbar. The score matrix was never mean-centred. Component 1 consequently carries 91.1 % of the variance with all 12 patient loadings and all 1,476 ligand–receptor loadings of the same sign: it is a scale term reflecting overall communication magnitude, not a latent communication programme, and it should be read as such. Components 2–5 carry the sign structure. A mean-centred re-derivation is named in Section 4.8 as committed follow-up work. SVD is not a non-negative tensor factorisation and no result in this manuscript is described as one.

Figure S4. Top 50 tokens by direction-resolved connectivity score, with resolved geometry (Layer L12)

(Submitted Figure S5; renumbered after the deletion of the Geneformer supplementary figure.)

Tokens sorted by S = z(primary) − $\lambda \cdot$ z(sibling) at λ = 1.0, where z(primary) is the LINCS L1000 connectivity z-score for the proliferation/migration contrast of this study and z(sibling) the same quantity for the companion apoptosis/ROS contrast. Rank is the competition rank among all 5,332 tokens, so tied tokens share a rank. Ranks 1–25 are drawn in the left block and 26–50 in the right. The figure prints six columns (rank; token; S at λ = 0.5 / 1.0 / 2.0; resolved geometry) and the remaining fields of the shortlist (token type; z(primary); z(sibling); minimum BH-adjusted p on the primary axis; DGIdb v5 regulatory-approval flag; DGIdb curated interaction count) are given in **Table S6**.

Reading the figure. A dagger (†) after a token marks the DGIdb v5 regulatory-approval flag described in *(iii)*. Pink-shaded rows are LINCS plate barcodes, not compounds, as set out in *(iv)*. Gefitinib and monobenzone are set in bold. The in-figure footnote is deliberately short and states only what a reader needs while reading the table itself — the definition of S and the fact that S is direction-blind; every other definition and correction is given here.

Four corrections and one new column relative to the previous figure. *(i) Resolved geometry is new.* Reversal = the LINCS down-signature intersects the tumour-up gene list (or up intersects down); mimicry = the same-direction intersections; ns = no primary-axis term reaches BH padj < 0.05. The submitted selectivity score is direction-agnostic and cannot distinguish the two: of these 50 tokens only 44 have any significant primary-axis term (19 reversal-only, 23 mimicry-only, 2 significant in both), and monobenzone (reversal), mepacrine (mimicry) and ku-c104230 (mimicry) all score exactly 5.340752 at λ = 1.0. Overall, 79 of 5,332 tokens (1.48 %) reach padj < 0.05 on the primary axis and none reaches padj < 0.05 anywhere on the sibling axis (minimum padj 0.2552), so the $\lambda \cdot$ z(sibling) term subtracts a null quantity and the Q1–Q4 quadrant framing of the submitted Figure 11A rests on a null axis. *(ii) The λ profile for gefitinib is corrected* to 4.516348 / 4.830408 / 5.458528 at λ = 0.5 / 1.0 / 2.0 (competition ranks 18 / 17 / 5). The submitted table printed 4.95 / 4.83 / 4.59: only λ = 1.0 was right, and the printed profile decreased with λ whereas the true profile increases, because z(sibling) is negative. Every other row checked matches the deposited table to two decimal places, so the error was specific to that row. *(iii) The approval flag is relabelled.* DGIdb v5's `approved` field is a general regulatory-approval flag, not FDA-specific — mofezolac is approved in Japan only and bretylium is discontinued in the United States. DGIdb was queried for the top 30 tokens only; it returned a record for 9 of them and an approval flag for 4. The remaining 5,302 tokens were never annotated, so no rank "among approved compounds" is well defined, and the submitted claim that gefitinib ranked first among FDA-approved compounds is withdrawn. `atc_class` and `approval_year` came back empty for all 30 annotated tokens; those two columns are deleted rather than printed empty. Gefitinib's ATC code L01EB01 is correct but its source is the WHO ATC index, not DGIdb. *(iv) Plate barcodes are separated from compound codes.* Rows shaded pink are LINCS plate barcodes, not compounds: the parser's first alphanumeric token for the `Chem_Pert_up/down` libraries is the plate identifier, so such a row's score is a maximum over thousands of unrelated perturbagens. Two of the top 50, including the token at rank 1, are barcodes. The submitted label "LINCS-internal compound code" conflated these with genuine internal compound codes such as *epz-020411*; they are different problems.

Figure S5. CRISPR essentiality detail on the corrected cell-line panel (Layer L14)

(Submitted Figure S6; renumbered.)

The metric is Chronos throughout. The submitted figure and caption referred to "median CERES"; DepMap retired CERES after release 21Q2 and Public 24Q4 ships Chronos gene effects. All three occurrences are corrected and the rendered panels carry no burned-in CERES label.

The cell-line panel is corrected. The published 88-line panel was the `OncotreeLineage == "Head and Neck"` selection unioned with 17 hard-coded model identifiers, and all 15 findable hard-coded identifiers resolve to non-head-and-neck lines — 11 of the 88 lines (12.5 %) are pancreatic, lymphoid, bladder, lung, bowel, ovarian or peripheral-nervous-system models. The primary stratum is now the 53 lines with `OncotreeSubtype == "Oral Cavity Squamous Cell Carcinoma"` and CRISPR data; the 77-line head-and-neck lineage stratum is retained as a sensitivity analysis.

(A) Median Chronos gene effect per candidate gene, ranked, across the 53 oral-cavity SCC lines for the 90-gene candidate list (L12-predicted drug targets plus the eight wet-lab anchors); 88 of 90 genes are present in the 24Q4 matrix — MAP4K5 and UHRF1 are genuinely absent, verified against the file header, and are not a symbol-matching artefact. Bars are coloured by bucket: essential (median < −1.0), moderately essential (−1.0 to −0.5), not essential (≥ −0.5). (B) Cross-line essentiality fraction. Each point is one gene; size ∝ number of lines tested; colour = median Chronos gene effect; the most essential genes are labelled. Effect of the panel correction — reported numerically, not plotted. Figure S5 has two panels, A and B. The before/after comparison is given here and in Section 3.11 rather than as a third panel, because it is a two-number result that a scatter plot would not clarify: published versus corrected medians across all 88 measured genes give Spearman $\varrho = 0.9971$, median $|\Delta \text{ median}| = 0.0074$, maximum 0.082 (CRKL). Two genes change bucket: GMNN leaves the essential bucket (median −0.497 in the oral-cavity stratum and −0.491 in the head-and-neck stratum, above the −0.5 threshold in both) and RELA enters it in the oral-cavity stratum only (−0.549, essential in 50.9 % of lines; −0.492 and non-essential in the 77-line stratum), so RELA should be read as an oral-cavity-specific, borderline call. The count of genes called essential is coincidentally unchanged at 12. The layer's substantive result is unaffected and the EGFR result strengthens: median Chronos −0.677, essential in 69.8 % (37/53) of oral-cavity lines against 61.4 % (54/88) on the erroneous panel.

Figure S6. Sensitivity analyses

(Submitted Figure S7; renumbered.)

(A) Gene-sets dropped per pathway library after anchor exclusion in the GSEA filter (Layer L9) — Reactome_Pathways_2024 contributed 2, GO_Biological_Process_2023 contributed 13. (B) The dropped gene-sets enumerated, with library, gene-set name, set size before exclusion and anchor-overlap count. All are small sets (≤ 5 members) that fall below the pre-registered `min_size = 5` once the anchor driving their inclusion is removed. (C) λ-sensitivity sweep across the top 30 tokens by the $\lambda = 1.0$ ranking; each line is one token. Corrected λ values throughout, including the gefitinib profile (4.516348 / 4.830408 / 5.458528 — increasing, not decreasing, in λ). (D) Rank-shift histogram between λ values, anchored on the top 50 at $\lambda = 1.0$. Interpretation corrected. Small rank shifts across λ were previously presented as evidence of a stable ranking. They are substantially an artefact of ties: across 5,332 tokens there are only 53 distinct z(primary) values, 15 distinct

z(sibling) values and 140 distinct $\lambda = 1.0$ selectivity values. Rank differences of a few places are not meaningful, and the λ-stability claim is reported with that caveat.

Figure S7. Internal and external validation of the prognostic layer (Layer L7)

The submitted manuscript reported in-sample hazard ratios and Cox p-values with no internal or external validation. This figure supplies both, in answer to Reviewer 2's fourth point. Panels A–G are internal and use the corrected primary-tumour-only TCGA cohorts (n = 171 / 181 / 512); panel **H** is the external test in GSE41613 and is the only panel of this figure that uses data external to TCGA. All panels use seed 20260521.

(A) Discrimination. Apparent (in-sample), 5 × 5-fold cross-validated (full feature selection repeated inside every fold) and 500-resample bootstrap optimism-corrected Harrell C-index per cohort. Apparent C = 0.651 / 0.633 / 0.635 falls to a cross-validated 0.506 ± 0.021 / 0.518 ± 0.021 / 0.568 ± 0.013 and a bootstrap-corrected 0.557 / 0.543 / 0.607 for STRICT / BROAD / PAN_HNSC. In the pre-registered oral-cavity cohort the signature is indistinguishable from a coin toss. CV optimism is 0.146 / 0.115 / 0.067; bootstrap optimism is 0.094 / 0.090 / 0.028 — the two are different quantities and are labelled as such. (B) In-sample label-permutation null of the hazard ratio, 1,000 iterations per cohort, with the observed HR marked. Empirical p = 0.809 (STRICT), 0.688 (BROAD), 0.0010 (PAN_HNSC). In STRICT and BROAD the median null HR (1.702 and 1.696) exceeds the observed HR (1.575 and 1.612). (C) Fraction of label-permuted runs reaching Cox $p < 0.05$ — in sample and out of fold. In sample: 100.0 % (STRICT), 99.9 % (BROAD), 100.0 % (PAN_HNSC). Out of fold: 0.5 % / 0.5 % / 1.5 %. This is the most consequential panel in the supplementary set: at these sample sizes the in-sample Cox p-value of a LASSO-selected signature carries no evidential weight, and the discrepancy between the two rows is the entire correction. (D) Out-of-fold label-permutation null of the cross-validated C-index, 200 iterations (resolution floor $p \geq 1/201 \approx 0.005$). Empirical p = 0.428 (STRICT), 0.229 (BROAD), 0.0050 (PAN_HNSC). Only the pan-HNSC sensitivity cohort separates from its null. The corresponding test on the out-of-fold HR gives p = 0.458 / 0.015 / 0.0050; BROAD's two statistics disagree and the conservative reading is taken. (E) Coxnet regularisation path. Number of non-zero features at each of the 20 alphas, with those satisfying the pre-registered 5–15-feature rule marked. Only 1 (STRICT), 2 (BROAD) and 3 (PAN_HNSC) alphas satisfy it, and in STRICT the non-zero count jumps 3 → 9 → 19 between adjacent alphas: the rule is a near-deterministic pick, not a search. Identical before and after correction. (F) Feature-selection frequency across the 25 cross-validation folds for the features retained by the full-data fit. Four of nine final STRICT features are selected in ≥ 50 % of folds (MMP3 0.96, DKK1 0.88, PCOLCE2 0.64, SFRP1 0.56); 4 of 8 in BROAD; 6 of 6 in PAN_HNSC (all ≥ 0.64). Only in the pan-HNSC cohort is the whole signature stable. (G) Cross-cohort overlap of the refit feature sets (Jaccard): STRICT–BROAD 0.545, BROAD–PAN 0.400, STRICT–PAN 0.250. Three features are common to all three cohorts (DKK1, PCOLCE2, SFRP1). (H) External validation in GSE41613. Harrell C-index with 95 % bootstrap confidence interval (2,000 patient-level resamples) for each of the six pre-declared cells — three cohort signatures (STRICT, the pre-registered primary; PAN-HNSC and BROAD, sensitivity variants) × two standardisation conventions (z, within-cohort gene-wise z-score; raw, unstandardised quantile-normalised $\log_2$) — applied to 97 HPV-negative oral-cavity squamous cell carcinomas with 51 deaths (GSE41613, GPL570). The TCGA-derived LASSO-Cox coefficients were frozen: nothing was refit, reselected or retuned in this cohort, and all signature genes are represented on the platform (STRICT 9/9, PAN-HNSC 6/6, BROAD 8/8). The dashed vertical line at C = 0.50 marks no discrimination; because the C-index is computed on the risk score directly, a value below 0.50 would indicate a reversed direction relative to TCGA. Coloured points mark the pre-registered primary (STRICT, orange) and the only signature that survived internal validation (PAN-HNSC, blue); grey points are the remaining pre-declared cells. The inset gives hazard ratios per standard

deviation of the score from Cox models adjusted for age band and sex. The pre-registered primary does not validate (STRICT · z: C = 0.562, 0.475–0.649; adjusted HR 1.280, 0.966–1.697, p = 0.085; median-split log-rank p = 0.301), whereas PAN-HNSC discriminates on the continuous score (C = 0.625, 0.530–0.708; adjusted HR 1.630, 1.176–2.259, p = 0.0034). The median-split log-rank test is non-significant for every cell, PAN-HNSC included (p = 0.067), so no high-/low-risk group claim is supported by this panel. Panels A–G report internal validation.

Two disclosures that belong with this figure. Features were not standardised before the L1 penalty (`CoxnetSurvivalAnalysis(normalize=False)`, never overridden); per-feature standard deviations span 0.30–2.96 (a factor of 9.5–10.1) and the selected features' median SD sits at the 96.6th–98.4th percentile of the pool, so the unstandardised penalty systematically favours the highest-variance genes. And AREG, an EGFR ligand and therefore a direct interaction partner of an excluded anchor, is selected only in PAN_HNSC — a residual-leakage disclosure that bears on the choice not to promote that cohort to primary — a choice that panel H does not reverse. An external outcome cohort is now analysed (panel H): GSE30784 and GSE37991 carry no survival data, so the external test uses GSE41613, in which the pre-registered primary signature is null and only the pan-HNSC sensitivity variant discriminates — and it does so across a scope mismatch, since GSE41613 is oral-cavity and HPV-negative while PAN-HNSC was fitted on anatomically unrestricted, partly HPV-positive disease (§2.2.6, §3.6, §4.4).

End of supplementary figure captions.
