# Supplementary material for: The Dual Face of Gingival Mesenchymal Stem Cell Paracrine Signalling in Oral Squamous Cell Carcinoma: A Pro-Tumour Transcriptional Programme and a Hypothesis-Generating Drug-Repurposing Screen
Source: Cells. 2026 Aug 26;15(17):1538. doi: 10.3390/cells15171538 (PMC13565543; doi:10.3390/cells15171538)
Supplement: Supplementary file 1 [file cells-15-01538-s001.zip › 05_SUPPLEMENTARY_TABLES.pdf]

Supplementary Tables

Companion document to the manuscript "*The dual face of gingival mesenchymal stem cell paracrine signalling in oral squamous cell carcinoma: a pro-tumour transcriptional programme and a hypothesis-generating drug-repurposing screen*".

Table S1. RT-qPCR primer sequences

The nine-assay panel used in Section 2.1.8 of the main text: eight targets spanning the angiogenesis, EMT, ECM-remodelling, chemotaxis, cell-cycle and receptor-tyrosine-kinase programmes implicated in OSCC progression, with GAPDH as the single reference gene for $2^{-\Delta\Delta Cq}$ normalisation. Sequences are given 5′ → 3′ and are identical to those printed in main-text Table 1.

Gene	Forward (5′ → 3′)	Reverse (5′ → 3′)	RefSeq transcript	Amplicon (bp)
GAPDH	GAAGGTGAAGGTCGGAGTC	GAAGATGGTGATGGGATTTC	NM_002046.7	226
VEGFA	AGGGCAGAATCATCACGAAGT	AGGGTCTCGATTGGATGGCA	NM_001025366.3	75
TGFB1	CTAATGGTGGAAACCCACAACG	TATCGCCAGGAATTGTTGCTG	NM_000660.7	209
MMP9	TGTACCGCTATGGTTACACTCG	GGCAGGGACAGTTGCTTCT	NM_004994.3	97
CXCL12	ATTCTCAACACTCCAAACTGTGC	ACTTTAGCTTCGGGTCAATGC	NM_000609.7	88
CCND1	GCTGCGAAGTGGAAACCATC	CCTCCTTCTGCACACATTTGAA	NM_053056.3	135
PCNA	CCTGCTGGGATATTAGCTCCA	CAGCGGTAGGTGTCGAAGC	NM_002592.2	109
MYC	GTCAAGAGGCGAACACACAAC	TTGGACGGACAGGATGTATGC	NM_002467.6	162
EGFR	AGGCACGAGTAACAAGCTCAC	ATGAGGACATAACCAGCCACC	NM_005228.5	177

Assay conditions. SYBR Green Master Mix (Thermo Fisher Scientific); biological triplicate ($n = 3$), each assayed in technical triplicate with the donor as the unit of analysis; ΔCq normalised to GAPDH; relative expression by $2^{-\Delta\Delta Cq}$; one-way ANOVA with Tukey's post-hoc test per gene across treatment conditions.

What the panel does and does not support. Amplicon lengths were determined by in-silico PCR against every human RefSeq transcript of the target gene, and specificity was confirmed by BLAST; each pair yields a single product in its intended gene. Amplification efficiencies were **not** determined from standard curves, and GAPDH stability under GMSC-conditioned medium was **not** assessed. Because $2^{-\Delta\Delta Cq}$ assumes comparable efficiencies between target and reference, and because all eight targets move in the same direction in all three treatment arms, a global normalisation shift remains a coherent alternative explanation for part of the effect size — though not for the relative ordering across genes. The panel should be read as an internally consistent set of relative, direction-bearing measurements rather than as absolutely calibrated fold-changes (main text Section 4.6.1).

Table S2. Public-cohort definitions and sample sizes (Layers L1–L3)

S2.1. TCGA-HNSC cohort variants

Variant	Definition	Tumour (n)	Normal (n)	Total VST columns	Survival model (n)	Events
STRICT	HPV-negative oral cavity (oral tongue, floor of mouth, buccal; ICD-10 C00, C02–C06); **pre-registered primary**	171	13	184	**171**	**65**
BROAD	STRICT + HPV-unknown oral cavity	183	13	196	**181**	**67**
PAN_HNSC	All HNSC anatomic sub-sites; **legacy proxy, sensitivity**	518	44	562	**512**	**215**

Sample-type restriction. The differential-expression layer legitimately uses both arms. The earlier survival models did not: they were fit on all VST columns, so 13, 13 and 44 solid-tissue normals entered the Cox and Kaplan–Meier models and, being patient-matched (13/13, 13/13 and 43/44 duplicated a patient already present as a tumour), also double-counted patients and deaths. Normal-tissue "events" are the donor's death, and their event rate is far higher than the tumour arm's (69.2 % vs 38.0 % in STRICT), so their inclusion was not neutral. The models are refit after restricting to GDC **sample type 01 (Primary Tumor)** before any signature scoring. Earlier drafts additionally gave BROAD as both 194 and 196 in different places, and PAN_HNSC as both 556 and 562; the corrected counts above are the number of patients actually entering each model.

The three variants are **strictly nested**: STRICT ⊂ BROAD ⊂ PAN_HNSC, with Jaccard on primary tumours of 0.934 (STRICT–BROAD), 0.353 (BROAD–PAN) and 0.330 (STRICT–PAN). BROAD adds 12 tumours to STRICT. Agreement across the three variants is therefore not independent replication and is no longer described as such.

Data release: GDC v45.0 (2025-12-04). HPV status confirmed against the Liu *et al.* PanCanAtlas Clinical Data Resource via the cBioPortal Datahub mirror.

Cohort-definition difference from the companion study. The companion study reports a cohort of the same name (TCGA-HNSC strict oral-cavity HPV-negative) comprising 217 primary tumours, one metastatic sample and 15 normals. The two cohorts are defined differently and neither count is an error. Our STRICT cohort applies the oral-cavity restriction by ICD-10 code only and additionally requires an explicit HPV-negative molecular call from the PanCanAtlas Clinical Data Resource; the companion's deposited cohort script infers HPV-negativity from anatomic subsite alone and resolves subsite from the free-text organ-of-origin field where the ICD-10 code is absent. Applying only the anatomic restriction to our own sample table returns 194 primary tumours, one metastatic sample and 15 normals (the same single metastatic sample and the same 15 normals the companion reports) and adding the molecular HPV-negative requirement gives the 171 / 13 used here (§2.2.2). Sources: `results/parquet/r1_corrected/r1_L7_sample_filter_audit.csv`, `r1_L7_event_rates.csv`, `r1_L7_cohort_nesting.csv`.

S2.2. GEO cohorts (Layer L2, random-effects meta-analysis)

GEO ID	Platform	Anatomic site	Tumour (n)	Normal (n)	Genes tested
GSE30784	Affymetrix HG-U133 Plus 2.0 (GPL570)	Oral cavity / oropharynx	167	45	21,755
GSE37991	**Illumina HumanRef-8 v3.0 (GPL6883)**	Oral tongue	40	40	17,544

Platform. GSE37991 is deposited on GPL6883, Illumina HumanRef-8 v3.0. The series matrix records **GPL6883, Illumina HumanRef-8 v3.0**. REML pooling across the two cohorts; per-cohort weights, τ^2 and Higgins I^2 in `results/parquet/L2_geo_meta_de.parquet` (per-cohort statistics in `L2_geo_per_cohort_de.parquet`).

GSE25099 was excluded after acquisition because its platform (GPL5175) lacked a probe-to-gene mapping in our cache. Neither GEO cohort carries outcome data, which is why the external validation of the prognostic signature uses a third deposit, GSE41613 (Table S9.4, §2.2.6), rather than either of these two. The companion study included GSE25099 in its own three-cohort meta-analysis, so this exclusion reflects our cache state rather than a property of the series (§4.7 ix).

S2.3. CPTAC HNSCC proteomic subset (Layer L3)

Source	Modality	Tumour (*n*)	Normal (*n*)	Protein rows	Gene symbols after collapse	Anchors retained
CPTAC HNSCC proteome (PNNL `cptac` 1.5.14, fetch date 2026-05-21)	TMT LC-MS/MS, log_2 median-centred	116	66	12,223	**11,581**	8 of 8

New disclosure. 642 duplicate gene symbols were collapsed on **maximum sample coverage** (n_tumour + n_normal), tie-broken on adjusted p then row order. This rule was chosen because it is the only candidate rule that does not select on the statistic being tested. None of the eight anchors is duplicated in any of the three tables, so the rule cannot affect the anchor results, and three-way sign concordance is stable at 88.7–89.8 % across nine alternative rules. Proteome-wide mRNA–protein concordance is **Spearman ϱ = 0.517 (0.52) on 11,447 gene–protein pairs**, 69 % sign-concordant; earlier text gave "ϱ ≈ 0.4" in two places against ϱ = 0.52 in the Figure 4B annotation (Reviewer 3 Major 3(b)). The anchor-only concordance (ϱ = 0.98, n = 8) is a different quantity and is reported separately. Source: `results/parquet/L3_cptac_protein_da.parquet`.

S2.4. External prognostic cohort (Layer L7 external validation)

GEO ID	Platform	Anatomic site / HPV	Patients (*n*)	Deaths (all-cause)	Median follow-up	Covariates deposited	Covariates absent
**GSE41613**	Affymetrix HG-U133 Plus 2.0 (GPL570)	Oral cavity, **HPV-negative** by design	97	51	54.4 months (range 0.46–85.03)	Stage I/II vs III/IV; age **band** (19–39 / 40–49 / 50–59 / 60–88); sex; treatment modality (uni- vs multi-modality)	Grade, margin status, smoking, exact age

Composition: stage III/IV 56 vs I/II 41; male 66 / female 31; age bands 60–88 (47), 50–59 (28), 40–49 (16), 19–39 (6); treatment multi-modality 53, uni-modality 43, unknown 1. Vital status is Alive 46, dead of oral cancer 30, dead of other cause 14, dead of unknown cause 7; **the endpoint used is all-cause overall survival**, matching the TCGA definition used internally, not the disease-specific endpoint the original authors modelled. This deposit shares no sample with any other layer, is used **once** with frozen coefficients, and no second external cohort was screened. Series matrix downloaded 2026-07-27, md5 `ad645ea2d7fac58a141cc1e9756ca0d5`; 54,613 probes → 45,108 with a symbol → **21,755 genes** after max-mean collapse against the same pinned GPL570 annotation used for the L2 cohorts. Results in Table S9.4 and Figure S7H; method in §2.2.6.

Table S3. Curated GMSC secretome v1.0

The curated GMSC secretome (**46 factors**) used as the source-ligand set in Layers L4 (STRING PPI), L5 (NicheNet decoder) and L10 (LIANA filter), each row carrying an explicit PubMed citation.

Full table with per-row PMID provenance: `data/curated/gmsc_secretome_v1.csv` (previously mis-cited as `results/tables/supplementary/S3_gmsc_secretome_v1.csv`, which does not exist). **All three ligand-**

nomination layers and the spatial layer draw their candidates from this one list, which is why their agreement is not independent evidence (Table 3, and Section 4.2). Four of the 46 factors (TGFB1, VEGFA, MMP9, CXCL12) collide with the wet-lab anchors, so the anchor-blind layers can only ever draw from 42.

10-row preview:

Ligand	Class	Evidence type	Representative reference (PMID)
TGFB1	Cytokine	Proteomics + transcriptomics	16778041
TGFB2	Cytokine	Proteomics	18786420
IGF1	Growth factor	Proteomics	18621737
FGF2	Growth factor	Proteomics	18621737
IL6	Cytokine	Proteomics + ELISA	16113402
HGF	Growth factor	Proteomics	18621737
PDGFB	Growth factor	Proteomics	18621737
MMP2	Protease	Zymography + transcript	11279140
MMP9	Protease	Zymography + transcript	11279140
THBS1	ECM glycoprotein	Proteomics	17786257

Table S4. STRING shortest-path output (Layer L4)

1,236 significant source–target pairs at empirical p < 0.05 against 200 degree-preserving rewires (`networkx.double_edge_swap`, 2 × edge-count swap attempts); per-pair empirical p = (null count + 1)/201. Sources: curated GMSC secretome minus the eight wet-lab anchors (42 ligands). Targets: union of Hallmark EMT / Myc Targets V1 / Myc Targets V2 / G2-M Checkpoint / mTORC1 / Hypoxia / PI3K-Akt-mTOR with KEGG focal adhesion / cell cycle / TGF-β / PI3K-Akt / MAPK / Ras / Wnt / pathways in cancer, restricted to TCGA-DE-detected genes and excluding the eight anchors. Distance ∈ {1, 2, 3, 4}.

Full table: **`results/parquet/L4_string_paths.parquet`**. The 22 MB rewiring null distribution (`L4_string_null_distribution.parquet`) is deposited to the Zenodo archive only. 10-row preview ranked by empirical p ascending:

Source	Target	Distance	Empirical p	Source class	Target class
DKK1	CAPRIN2	2	0.00498	Wnt antagonist	Wnt pathway scaffold
DKK1	DKK3	2	0.00498	Wnt antagonist	Wnt antagonist
DKK1	SOSTDC1	2	0.00498	Wnt antagonist	BMP/Wnt antagonist
THBS1	LOX	2	0.00498	ECM glycoprotein	ECM cross-linker
IGFBP3	ERRFI1	2	0.00498	IGF-axis regulator	EGFR-pathway negative regulator
HGF	MET	1	0.00498	HGF/MET pathway	HGF receptor
FN1	SDC1	2	0.00498	ECM glycoprotein	HSPG receptor
COL1A1	DDR1	2	0.00498	Collagen	Collagen receptor RTK
TGFB2	SMAD3	2	0.00498	TGF-β family	TGF-β downstream effector
TIMP1	CD63	1	0.00498	MMP inhibitor	Tetraspanin (TIMP-1 receptor)

STRING nominates 40 of the 46 secretome ligands — 40 of the 42 (95.2 %) actually available to it after the anchor collisions. This layer establishes topological proximity in a literature-aggregated interaction graph; it does not establish that any interaction occurs in OSCC tissue (Table 3).

Table S5. Ligand–receptor pairs and SVD component loadings (Layers L10, L10b)

LIANA consensus aggregation (CellPhoneDB + NATMI + Connectome) on GSE103322 returned **841 Fibroblast → Malignant ligand–receptor pairs**, of which **153 have a ligand present in the curated GMSC secretome**. That 153-pair set is the pre-specified universe carried forward to the spatial layer. The 12-patient decomposition retained **five components**.

Terminology corrected (Reviewer 3 Minor 5). No tensor was constructed and Tensor-cell2cell was not run; the `cell2cell` v0.8.4 API was incompatible with our environment and a **singular-value decomposition** of the 12 patient × 1,476 ligand–receptor score matrix was substituted. The matrix was **not mean-centred**, so component 1 carries 91.1 % of the variance with all patient and all ligand–receptor loadings of one sign — a scale term, not a latent communication programme. Components 2–5 carry the sign structure. Sources: `results/parquet/L10_liana_ccc.parquet` (all 841 pairs), `L10_liana_filtered_to_gmsc_secretome.parquet` (the 153), `L10b_factor_loadings.parquet`.

Top 10 LIANA pairs by aggregated `lrscore`:

Source	Target	Ligand	Receptor	LR mean	lrscore	Specificity rank	Magnitude rank
Fibroblast	Malignant	VIM	CD44	5.789	0.923	0.052	0.00021
Fibroblast	Malignant	TIMP1	CD63	5.329	0.920	0.102	0.00043
Fibroblast	Malignant	LGALS1	ITGB1	5.146	0.914	0.077	0.00057
Fibroblast	Malignant	COL1A2	SDC1	4.824	0.913	0.001	0.00109
Fibroblast	Malignant	HMGB1	SDC1	4.692	0.910	0.008	0.00140
Fibroblast	Malignant	COL1A2	CD44	4.517	0.907	0.008	0.00199
Fibroblast	Malignant	COL1A1	SDC1	4.449	0.906	0.002	0.00234
Fibroblast	Malignant	MIF	CD44_CD74	4.468	0.906	1.000	0.00219
Fibroblast	Malignant	HLA-A	APLP2	4.492	0.905	0.272	0.00211
Fibroblast	Malignant	FN1	SDC1	4.380	0.904	0.003	0.00278

Table S6. Direction-resolved drug-repurposing shortlist, top 50 (Layer L12)

5,332 tokens were scored against three LINCS L1000 chemical-perturbation libraries (`Consensus_Sigs`, `Chem_Pert_up`, `Chem_Pert_down`) on both contrasts — this study's proliferation/migration contrast and the companion apoptosis/ROS contrast. The token universe is approximately 5,297 LINCS consensus perturbagens plus 25 plate identifiers admitted in error by the name parser, with the remainder unresolved name variants; no coverage figure of 5,064 of 5,332 tokens (95 %) with connectivity data for both axes is reproduced by the deposited output (Section 3.10). $S(\text{token}) = z(\text{primary}) - \lambda \cdot z(\text{sibling})$ at $\lambda \in \{0.5, 1.0, 2.0\}$. **Ranks below are competition ranks, so tied tokens share a rank.**

Withdrawn: "rank 1 among FDA-approved compounds". That claim was false and was falsified by the submitted table itself, which printed monobenzone at rank 10 carrying the same approval tick three rows above the gefitinib row. DGIdb v5 was queried for the top 30 tokens only; it returned a record for 9 and an approval flag for **4** (monobenzone, gefitinib, mofezolac, bretylium), so the "approved universe" is four compounds and **5,302 of 5,332 tokens were never annotated at all**. Among the four, **monobenzone ranks above gefitinib** at every λ. The defensible statement is: *the highest-ranked token carrying a DGIdb regulatory-approval flag among the 30 annotated is monobenzone; gefitinib is second.* The flag is a **general regulatory-approval flag, not FDA-specific** (mofezolac is approved in Japan only; bretylium is discontinued in the US) and the column is relabelled accordingly.

Corrected λ profile for gefitinib: 4.516348 / 4.830408 / 5.458528 at λ = 0.5 / 1.0 / 2.0 (competition ranks 18 / 17 / 5). The submitted row printed 4.95 / 4.83 / 4.59 — only λ = 1.0 was right, and the printed profile *decreased* in λ whereas the true profile *increases*, because z(sibling) is negative. Every other row checked matches the deposited table to two decimal places.

`approval_year` and `atc_class` are deleted, not printed empty: DGIdb v5 returned neither for any of the 30 annotated tokens, so the submitted claim that DGIdb supplied ATC class and approval year was false as to both. Gefitinib's ATC code L01EB01 is correct, sourced to the WHO ATC index.

Resolved geometry is new and is the column that matters. Reversal = the LINCS down-signature intersects the tumour-up gene list (or up intersects down); mimicry = the same-direction intersections. **The selectivity score cannot distinguish the two:** monobenzone (reversal), mepacrine (mimicry) and ku-c104230 (mimicry) all score *exactly* 5.340752 at λ = 1.0. Of the top 50, 19 are reversal-only, 23 mimicry-only, 2 significant in both and 6 have no significant primary-axis term. Of all 5,332 tokens, 79 (1.48 %) reach padj < 0.05 on the primary axis and **none reaches padj < 0.05 anywhere on the sibling axis** (minimum padj 0.2552), so $\lambda \cdot$ z(sibling) subtracts a null quantity. Rows marked *plate barcode* are LINCS plate identifiers, not compounds: their score is a maximum over thousands of unrelated perturbagens.

Full 5,332-row table: **`results/parquet/r1_corrected/L12_direction_resolved.parquet`** (CSV mirror alongside). Supporting audits: `L12_token_audit.csv`, `L12_rank_verification.csv`, `L12_reversal_only_reranking.csv`, `L12_axis_separability.csv`, `L12_gefitinib_drivers_both_DE_runs.csv`. The submitted `L12_dual_axis_selectivity.parquet` is retained for comparison. The previously cited `results/tables/supplementary/S6_drug_top50_selectivity.csv` **does not exist** and the pointer is withdrawn.

Preview at λ = 1.0 (12 of 50 rows):

Rank	Token	Token type	*S* λ=0.5	*S* λ=1.0	*S* λ=2.0	z primary	z sibling	Geometry	min BH padj (primary)	Approval flag (DGIdb v5)	DGIdb int.
1	ljp005	**plate barcode**	8.544	7.592	5.687	9.496	1.905	both	4.0×10^{-4}	not annotated	—
2	epz-020411	code-like name	8.487	6.481	2.470	10.492	4.011	reversal	2.0×10^{-4}	not annotated	—
3	rti-55	code-like name	7.258	6.427	4.764	8.090	1.663	reversal	0.0011	not annotated	—

Rank	Token	Token type	S λ=0.5	S λ=1.0	S λ=2.0	z primary	z sibling	Geometry	min BH padj (primary)	Approval flag (DGIdb v5)	DGIdb int.
4	isometheptene	compound name	5.767	5.532	5.063	6.002	0.470	reversal	0.0047	not approved	0
4	y-26763	code-like name	5.767	5.532	5.063	6.002	0.470	**mimicry**	0.0047	not annotated	—
8	ljp009	**plate barcode**	6.321	5.369	3.464	7.274	1.905	both	0.0019	not annotated	—
9	ku-c104230	code-like name	5.027	5.341	5.969	4.713	−0.628	**mimicry**	0.0118	not annotated	—
9	mepacrine	compound name	5.027	5.341	5.969	4.713	−0.628	**mimicry**	0.0118	not annotated	—
9	**monobenzone**	compound name	5.027	5.341	5.969	4.713	−0.628	reversal	0.0118	**approved**	3
12	nafcilline	compound name	7.836	5.181	−0.130	10.492	5.311	**mimicry**	2.0×10^{-4}	not annotated	—
13	ticarcillin	compound name	4.869	5.026	5.340	4.713	−0.314	reversal	0.0118	not approved	2
**17**	**gefitinib**	compound name	**4.516**	**4.830**	**5.459**	**4.202**	**−0.628**	**reversal**	**0.0170**	**approved**	**67**

On gefitinib specifically. Its single significant primary-axis term does have reversal geometry — the LINCS consensus "Gefitinib Down" set intersecting the tumour-up list, 9 of 245 genes, $P = 3.87 \times 10^{-6}$, padj = 0.0170 — so the direction is correct as far as it goes. However, **7 of the 9 driver genes are MAGE-family cancer–testis antigens** (largely the MAGEA cluster on Xq28), and removing that single co-regulated locus moves the hypergeometric P from 3.87×10^{-6} to **0.30**, against a BH threshold at that rank of 1.14×10^{-5}. Enrichr's Fisher test assumes gene independence, which one co-regulated locus violates. **No EGFR-pathway gene contributes**, and could not: EGFR is one of the eight excluded anchors, so the nomination is independent of EGFR biology by construction. The 67 DGIdb interactions are real and do include EGFR/ERBB2/3/4, but they did not generate the hit. Across the 78 genuine per-signature gefitinib rows, 25 are reversal and 17 mimicry against a 51.31 % reversal baseline (binomial $p = 0.18$) and **no row reaches padj < 0.05**. Under a direction-corrected reversal-only re-ranking gefitinib is 9th of 4,901 and monobenzone 5th, with the top four positions taken by unapproved tool compounds and a sympathomimetic. Nothing in this table supports a claim of demonstrated activity, and none is made anywhere in the manuscript.

Table S7. CRISPR essentiality per gene across 53 oral-cavity SCC cell lines (Layer L14)

(Submitted title: "across 88 HNSC cell lines".)

Primary stratum: 53 oral-cavity squamous cell carcinoma lines (`OncotreeSubtype == "Oral Cavity Squamous Cell Carcinoma"` with CRISPR data) × 90 candidate genes (L12-predicted drug targets plus the eight wet-lab anchors). **Sensitivity stratum: 77 head-and-neck-lineage lines.** DepMap Public 24Q4 **Chronos** gene-effect scores. Essentiality threshold: median Chronos < −0.5 (essential), < −1.0 (strongly essential).

Panel correction. The published 88-line panel was the `OncotreeLineage == "Head and Neck"` selection **unioned** (not overridden) with 17 hard-coded model identifiers, and all 15 findable hard-coded identifiers resolve to non-head-and-neck lines. **11 of the 88 lines (12.5 %)** were pancreatic (HPAFII, PANC0327), lymphoid (AMO1, SUPT1), bladder (SCABER), lung (HCC366, CHAGOK1, NCIH510), bowel (SW48), ovarian (OVCAR5) or peripheral nervous system (NB7). Of the intended lines, HN5 has no model with that stripped name; BICR-18 (ACH-000992) is in `Model.csv` as larynx SCC but has no `CRISPRGeneEffect` row; and CAL-62 is anaplastic thyroid carcinoma and was never head-and-neck. The corrected script reproduces the published parquet exactly on the 88-line union (max |Δ median| = 0.0), which is what proves the panel was that union.

Metric name corrected: Chronos, not CERES (DepMap retired CERES after 21Q2).

The correction barely moves the layer — Spearman ρ = 0.9971 between published and corrected medians over the 88 measured genes, median |Δ median| = 0.0074, maximum 0.082 (CRKL) — and the key claim strengthens. Two bucket changes: GMNN leaves the essential list (median −0.497 primary, −0.491 sensitivity) and RELA enters it **in the oral-cavity stratum only** (−0.549, 50.9 %; −0.492 and non-essential in the 77-line stratum), so RELA is a borderline, oral-cavity-specific call. The count of essential genes is coincidentally unchanged at 12 (11 in the sensitivity stratum).

Full tables: **`results/parquet/r1_corrected/L14_depmap_essentiality_PRIMARY_OCSC.parquet`**, `L14_depmap_essentiality_SENSITIVITY_HN.parquet`, with the published-versus-corrected comparison in `L14_depmap_before_after.csv`, panel membership in `L14_depmap_panel_membership.csv` and the hard-coded-identifier audit in `L14_depmap_published_panel_audit.csv` and `L14_depmap_intended_line_resolution.csv`.

20-row preview, primary stratum, ranked by median Chronos ascending:

Rank	Gene	In DepMap	*n* lines	Median Chronos	Essential in *n* lines	Essential fraction
1	PCNA	yes	53	−2.864	53	1.000
2	MYC	yes	53	−1.996	52	0.981
3	MTOR	yes	53	−1.137	52	0.981
4	CRKL	yes	53	−1.120	50	0.943
5	EIF4A1	yes	53	−1.094	52	0.981
6	CDK6	yes	53	−0.839	41	0.774
7	CCND1	yes	53	−0.821	43	0.811
8	EGFR	yes	53	**−0.677**	37	**0.698**
9	NUP62	yes	53	−0.634	37	0.698
10	CDK4	yes	53	−0.579	33	0.623
11	PIK3CA	yes	53	−0.553	30	0.566
12	RELA	yes	53	−0.549	27	0.509
13	GMNN	yes	53	−0.497	26	0.491
14	ERBB2	yes	53	−0.468	25	0.472

Rank	Gene	In DepMap	*n* lines	Median Chronos	Essential in *n* lines	Essential fraction
15	ERBB3	yes	53	−0.423	19	0.358
16	ARF1	yes	53	−0.413	21	0.396
17	KRAS	yes	53	−0.291	7	0.132
18	CBX1	yes	53	−0.257	2	0.038
19	MAPK14	yes	53	−0.249	3	0.057
20	MAPK1	yes	53	−0.208	4	0.075

Rank order changes at positions 3–7 and 12–13 relative to the submitted preview (MTOR and CRKL move above EIF4A1; CDK6 above CCND1; RELA above GMNN). Wet-lab anchors PCNA (rank 1), MYC (2), CCND1 (7) and EGFR (8) are essential in the majority of oral-cavity lines, and the L12-predicted targets MTOR, CRKL, CDK4/6 and PIK3CA are **essential** (the submitted note read "essentially") in the majority. **88 of the 90 candidate genes are present in the 24Q4 matrix**; MAP4K5 and UHRF1 are genuinely absent, verified against the file header. Figure presentation: main Figure 11A; ranked bars, cross-line scatter and the before/after comparison in **Figure S5A–C**.

This layer establishes that these genes are required for the fitness of oral-cavity SCC lines *in vitro*. It does not establish that GMSC paracrine signalling engages them, and it is not evidence for any drug (Table 3).

Table S8. Gene-sets dropped after anchor exclusion (Layer L9)

Pre-ranked GSEA was run with the eight wet-lab anchors removed from **both** the ranking statistic and each gene-set's membership. Sets falling below `min_size = 5` after exclusion were dropped per the pre-registered protocol: **15 sets across 2 libraries** (13 from GO Biological Process 2023, 2 from Reactome Pathways 2024). Figure presentation: **Figure S6A, S6B**.

[VERIFY BEFORE UPLOAD] This count must be re-confirmed against the **regenerated** anchor-blind audit log, which is being rebuilt after the removal of L11 and the addition of the missing `assert_no_anchor_leak("L12", …)` assertion. One value must appear in Section 2.2.13, the Table 2 caption, this table and the deposited log. Source: `results/parquet/L9_dropped_genesets.parquet`.

#	Library	Gene-set	Original size	Anchor overlap
1	GO_Biological_Process_2023	ERBB2-EGFR Signaling Pathway (GO:0038134)	5	1
2	GO_Biological_Process_2023	Negative Regulation Of Cellular Extravasation (GO:0002692)	5	1
3	GO_Biological_Process_2023	Positive Regulation Of Cardiac Muscle Cell Differentiation (GO:2000727)	5	1
4	GO_Biological_Process_2023	Positive Regulation Of Intrinsic Apoptotic Signaling Pathway By P53 Class Mediator (GO:1902255)	5	1
5	GO_Biological_Process_2023	Positive Regulation Of Receptor Binding (GO:1900122)	5	1
6	GO_Biological_Process_2023	Regulation Of Blood Vessel Remodeling (GO:0060312)	5	1
7	GO_Biological_Process_2023	Regulation Of Histone Deacetylase Activity (GO:1901725)	5	1
8	GO_Biological_Process_2023	Regulation Of Hyaluronan Biosynthetic Process (GO:1900125)	5	1
9	GO_Biological_Process_2023	Regulation Of Mast Cell Chemotaxis (GO:0060753)	5	1

#	Library	Gene-set	Original size	Anchor overlap
10	GO_Biological_Process_2023	Regulation Of Nitric Oxide Mediated Signal Transduction (GO:0010749)	5	2
11	GO_Biological_Process_2023	Regulation Of Retinal Ganglion Cell Axon Guidance (GO:0090259)	5	1
12	GO_Biological_Process_2023	Vascular Endothelial Growth Factor Receptor-2 Signaling Pathway (GO:0036324)	5	1
13	GO_Biological_Process_2023	Venous Blood Vessel Morphogenesis (GO:0048845)	5	1
14	Reactome_Pathways_2024	Drug-mediated Inhibition of CDK4 CDK6 Activity	5	1
15	Reactome_Pathways_2024	TFAP2 (AP-2) Family Regulates Transcription of Cell Cycle Factors	5	1

All dropped sets are small (≤ 5 members) and fall below the minimum size once the anchor that drove their overlap is removed. **Library versions are cited as Enrichr library names plus a fetch date, not as an MSigDB release**: the submitted claim of "MSigDB v2024.1.Hs" (five occurrences) is unsupported by any artefact in the workspace, whose provenance record holds only the Enrichr library names `MSigDB_Hallmark_2020`, `KEGG_2021_Human`, `Reactome_Pathways_2024` and `GO_Biological_Process_2023` with their fetch date.

Table S9. Internal and external validation of the prognostic layer (Layer L7)

The submitted manuscript reported in-sample hazard ratios and Cox p-values for the prognostic layer with no validation of any kind. This table supplies both the internal package (S9.1–S9.3) and the external test in GSE41613 (S9.4), in answer to Reviewer 2's fourth point. Internal estimates use the corrected primary-tumour-only TCGA cohorts; all estimates use `SEED = 20260521`, `lifelines` 0.30.3 and `scikit-survival` 0.27.0; Cox models are adjusted for age and sex; strata are median-split.

S9.1. All 15 cohort × signature cells

Five signatures × three cohort variants. **z-sum** = sum of standardised z-scores of the eight anchors (anchor-*dependent* consistency test); **LASSO-Cox** = the anchor-blind exploratory signature; the three **ssGSEA** signatures score MSigDB Hallmark sets with the eight anchors removed from each set's membership.

Cohort	*n*	Events	Signature	HR (95 % CI)	Cox *p*	BH *p* across the 15 cells	Log-rank *p*
STRICT	171	65	z-sum	1.259 (0.980–1.616)	0.0712	0.107	0.130
STRICT	171	65	**LASSO-Cox**	**1.575 (1.286–1.929)**	1.13×10^{-5}	7.20×10^{-5}	5.81×10^{-5}
STRICT	171	65	ssGSEA EMT	1.028 (0.799–1.322)	0.831	0.890	0.680
STRICT	171	65	ssGSEA Myc Targets V1	1.150 (0.896–1.476)	0.271	0.339	0.744
STRICT	171	65	ssGSEA G2-M Checkpoint	1.303 (1.008–1.685)	0.0435	0.0872	0.0164
BROAD	181	67	z-sum	1.267 (0.990–1.622)	0.0601	0.100	0.119
BROAD	181	67	**LASSO-Cox**	**1.612 (1.299–2.001)**	1.44×10^{-5}	7.20×10^{-5}	5.42×10^{-5}
BROAD	181	67	ssGSEA EMT	1.017 (0.793–1.304)	0.895	0.895	0.555

Cohort	n	Events	Signature	HR (95 % CI)	Cox p	BH p across the 15 cells	Log-rank p
BROAD	181	67	ssGSEA Myc Targets V1	1.202 (0.937–1.540)	0.147	0.201	0.732
BROAD	181	67	ssGSEA G2-M Checkpoint	1.360 (1.052–1.757)	0.0188	0.0563	0.0170
PAN_HNSC	512	215	z-sum	1.307 (1.133–1.509)	2.51×10^{-4}	9.40×10^{-4}	1.06×10^{-3}
PAN_HNSC	512	215	**LASSO-Cox**	**1.602 (1.389–1.847)**	8.50×10^{-11}	1.27×10^{-9}	1.71×10^{-10}
PAN_HNSC	512	215	ssGSEA EMT	1.164 (1.011–1.339)	0.0346	0.0865	0.238
PAN_HNSC	512	215	ssGSEA Myc Targets V1	1.153 (1.002–1.326)	0.0465	0.0872	0.300
PAN_HNSC	512	215	ssGSEA G2-M Checkpoint	1.037 (0.904–1.190)	0.605	0.698	0.372

All HRs in S9.1 are apparent (in-sample) estimates and must be read against S9.2. Three corrections to the submitted text follow from this table. (i) "Marginal significance only in PAN-HNSC" for the z-sum signature is false: the corrected p is 2.51×10^{-4}, 48-fold smaller, and it survives BH correction across the 15 cells. (ii) "Non-significant across all three cohorts" for the ssGSEA variants is false: four of the nine ssGSEA cells are nominally significant, though none survives BH correction across the 15 tests (BH p 0.056–0.087). (iii) The ssGSEA EMT hazard ratios in STRICT and BROAD **change direction** relative to the submitted values (0.89 and 0.88 → 1.028 and 1.017) once the normals are removed, and both ssGSEA G2-M cells gain nominal significance. Source: `results/parquet/r1_corrected/r1_L7_survival_results.parquet` (row-filter-only comparison in `r1_L7_survival_results_filter_only.parquet`).

S9.2. Internal validation of the LASSO-Cox signature

Cross-validation and permutation apply to the LASSO-Cox signature only, because it is the only signature whose construction involves data-dependent feature selection; **the complete selection procedure is repeated inside every fold and inside every bootstrap resample**, so no fold sees a feature chosen on its own outcomes.

Quantity	STRICT	BROAD	PAN_HNSC
Apparent (in-sample) C-index	0.651	0.633	0.635
**Cross-validated C-index (5 × 5-fold, mean ± SD)**	**0.506 ± 0.021**	**0.518 ± 0.021**	**0.568 ± 0.013**
CV optimism (apparent − CV)	0.146	0.115	0.067
**Bootstrap optimism-corrected C-index (500 resamples)**	**0.557**	**0.543**	**0.607**
Bootstrap optimism (± SD)	0.094 ± 0.030	0.090 ± 0.031	0.028 ± 0.018
Apparent HR (95 % CI)	1.575 (1.286–1.929)	1.612 (1.299–2.001)	1.602 (1.389–1.847)
**Out-of-fold HR (95 % CI)**	**1.014 (0.789–1.304)**	**1.198 (0.939–1.528)**	**1.361 (1.190–1.557)**
Out-of-fold Cox p	0.911	0.146	$\mathbf{7.05 \times 10^{-6}}$
In-sample permutation empirical p (1,000 iterations)	**0.809**	**0.688**	**0.0010**
Median HR under permuted labels	**1.702**	**1.696**	1.329
**Fraction of permuted runs reaching in-sample Cox $p < 0.05$**	**100.0 %**	**99.9 %**	**100.0 %**

Quantity	STRICT	BROAD	PAN_HNSC
Fraction of permuted runs reaching out-of-fold Cox $p < 0.05$	0.5 %	0.5 %	1.5 %
Out-of-fold permutation p on CV C-index (200 iterations)	0.428	0.229	**0.0050**
Out-of-fold permutation p on out-of-fold HR	0.458	0.015	**0.0050**
Non-zero features in the full-data fit	9	8	6
Median features selected per CV fold	7	6	6
Alphas on the 20-point path satisfying the 5–15-feature rule	1	2	3
External outcome cohort analysed	GSE41613 (S9.4)	GSE41613 (S9.4)	GSE41613 (S9.4)
External C-index in GSE41613 (z-score convention)	0.562 (0.475–0.649)	0.538 (0.451–0.622)	**0.625 (0.530–0.708)**

Reading. In the **pre-registered oral-cavity cohort the signature is null**: cross-validated discrimination is indistinguishable from a coin toss, the out-of-fold HR is 1.01 (p = 0.91), the permutation empirical p is 0.81, and the **median HR under permuted survival labels (1.702) exceeds the observed HR (1.575)**. Only the pan-HNSC **sensitivity** cohort separates from its null on both out-of-fold statistics. BROAD's two out-of-fold statistics disagree (CV-C p = 0.229 vs out-of-fold-HR p = 0.015, whose own Wald p is 0.146) and the conservative reading is taken. The out-of-fold permutation resolution floor is p ≥ 1/201 ≈ 0.005.

The line that matters most is the false-positive rate. At these sample sizes, **100 % of label-permuted runs of the same 5–15-feature LASSO-Cox pipeline reach in-sample Cox p < 0.05**, so the in-sample Cox p-value of a LASSO-selected signature carries no evidential weight here. The submitted claim that the anchor-blind LASSO signature *outperformed* the anchor-based z-sum, and that this refuted the circularity concern, is withdrawn: the advantage was partly manufactured by the normals (12 of 13 fell in the LASSO high-risk stratum and 13 of 13 in the z-sum and G2-M low-risk strata). The anti-circularity argument is re-grounded on **construction** — the anchors are excluded from the feature pool — not on effect size.

Two further disclosures. Features were **not standardised before the L1 penalty** (`CoxnetSurvivalAnalysis(normalize=False)`, never overridden); per-feature SD spans 0.30–2.96 (9.5–10.1-fold) and the selected features' median SD sits at the 96.6th–98.4th percentile of the pool, so the penalty systematically favours the highest-variance genes. And the 5–15-feature rule is satisfied by only one alpha in STRICT, where the non-zero count jumps 3 → 9 → 19 between adjacent alphas: it is a near-deterministic pick, not a search. Both were true of the submitted run as well as the corrected one. Sources: `r1_L7_cv_summary.csv`, `r1_L7_bootstrap_summary.csv`, `r1_L7_permutation_summary.csv`, `r1_L7_permutation_outoffold_summary.csv`, `r1_L7_alpha_grid.csv`, `r1_L7_feature_scale_audit.csv`, `r1_L7_normal_stratum_placement.csv`, `r1_L7_run_metadata.json` (all under `results/parquet/r1_corrected/`).

S9.3. Selected features and their stability

Selection frequency across the 25 cross-validation folds and across the 500 bootstrap resamples, for the features retained by the full-data fit.

Cohort	Feature	CV selection frequency	Folds selected	Bootstrap selection frequency
STRICT	MMP3	0.96	24/25	0.660
STRICT	DKK1	0.88	22/25	0.650
STRICT	PCOLCE2	0.64	16/25	0.374

Cohort	Feature	CV selection frequency	Folds selected	Bootstrap selection frequency
STRICT	SFRP1	0.56	14/25	0.386
STRICT	THBS1	0.40	10/25	0.186
STRICT	TGM2	0.40	10/25	0.226
STRICT	COMP	0.36	9/25	0.258
STRICT	HOXC10	0.28	7/25	0.322
STRICT	PTHLH	0.24	6/25	0.152
BROAD	MMP3	1.00	25/25	0.670
BROAD	DKK1	0.96	24/25	0.658
BROAD	SFRP1	0.64	16/25	0.470
BROAD	PCOLCE2	0.56	14/25	0.412
BROAD	HOXC10	0.36	9/25	0.322
BROAD	MCM2	0.32	8/25	0.156
BROAD	PTX3	0.20	5/25	0.198
BROAD	THBS1	0.16	4/25	0.134
PAN_HNSC	DKK1	1.00	25/25	0.970
PAN_HNSC	PTX3	1.00	25/25	0.612
PAN_HNSC	SFRP1	0.96	24/25	0.698
PAN_HNSC	**AREG**	0.76	19/25	0.488
PAN_HNSC	PCOLCE2	0.76	19/25	0.598
PAN_HNSC	SPOCK1	0.64	16/25	0.388

Only 4 of 9 STRICT features and 4 of 8 BROAD features are selected in at least half the folds; in PAN_HNSC all 6 are (≥ 0.64). Cross-cohort overlap of the three feature sets is Jaccard 0.545 (STRICT–BROAD), 0.400 (BROAD–PAN) and 0.250 (STRICT–PAN); three features are common to all three (DKK1, PCOLCE2, SFRP1) whereas the submitted text named two (DKK1, PCOLCE2). SGCG, named in the submitted feature list, drops out entirely after the correction — which is why row-filtering the published signature scores could not have repaired this defect and the model had to be refit.

Residual-leakage disclosure. AREG is an EGFR ligand, i.e. a direct interaction partner of one of the eight excluded anchors, and it is selected **only in PAN_HNSC**. Anchor exclusion removes the anchors themselves, not their immediate network neighbourhood: 10.0 % (56 of 562) of the retained feature pool are STRING high-confidence direct partners of an excluded anchor, and two selected features are among them (AREG; and MMP3, a paralogue of the excluded MMP9). This bears directly on the decision not to promote PAN_HNSC to primary. Sources: `r1_L7_feature_stability.csv`, `r1_L7_feature_overlap_between_cohorts.csv`, `r1_L7_lasso_features.parquet`.

S9.4. External validation in GSE41613 — all six pre-declared cells

The frozen TCGA-derived LASSO-Cox coefficients were applied once to an independent public cohort: **GSE41613** (GPL570, Affymetrix HG-U133 Plus 2.0; 97 HPV-negative oral-cavity squamous cell carcinomas, 51 deaths, median follow-up 54.4 months; series matrix downloaded 2026-07-27, md5 `ad645ea2d7fac58a141cc1e9756ca0d5`). **Nothing was refit, reselected or retuned in this cohort.** The endpoint is

all-cause overall survival, matching the internal TCGA definition and not the disease-specific endpoint the original authors modelled. Six cells — three signatures × two standardisation conventions — were declared before any score was related to outcome, and **all six are reported**. All signature genes are present on GPL570 (STRICT 9/9, PAN-HNSC 6/6, BROAD 8/8), so the pre-declared missing-gene rule was never invoked. n = 97 with 51 events for every cell; MV2 drops the one patient with treatment recorded as unknown (n = 96).

**Cell**	**Genes**	**C-index (95 % CI)**	**Log-rank *p* (median split)**	**HR/SD univariable (95 % CI)**	***p***	**HR/SD + age band, sex**	***p***	**HR/SD + age band, sex, stage, treatment**	***p***
**STRICT · z** (pre-registered primary)	9/9	0.562 (0.475–0.649)	0.301	1.233 (0.945–1.607)	0.122	1.280 (0.966–1.697)	0.085	1.182 (0.874–1.598)	0.277
**PAN_HNSC · z** (sensitivity)	6/6	**0.625 (0.530–0.708)**	0.067	1.512 (1.132–2.020)	0.0051	**1.630 (1.176–2.259)**	**0.0034**	**1.665 (1.165–2.381)**	**0.0052**
BROAD · z (completeness)	8/8	0.538 (0.451–0.622)	0.676	1.095 (0.841–1.427)	0.499	1.125 (0.855–1.480)	0.398	1.122 (0.831–1.515)	0.453
STRICT · raw	9/9	0.533 (0.450–0.616)	0.426	1.101 (0.850–1.425)	0.466	1.132 (0.863–1.485)	0.369	1.058 (0.792–1.414)	0.703
**PAN_HNSC · raw**	6/6	**0.627 (0.537–0.712)**	0.080	1.487 (1.112–1.989)	0.0074	**1.615 (1.158–2.254)**	**0.0048**	**1.612 (1.131–2.299)**	**0.0083**
BROAD · raw	8/8	0.519 (0.433–0.606)	0.311	1.059 (0.812–1.382)	0.672	1.087 (0.824–1.434)	0.556	1.048 (0.785–1.400)	0.752

z = within-cohort gene-wise z-score (the plan's primary handling of the cross-platform scale difference); **raw** = no gene-wise standardisation (the convention under which the coefficients were originally fitted). The C-index is computed on the risk score directly, so a value below 0.50 would mean the direction reverses relative to TCGA. The median split is at the cohort median of each score (48 high / 49 low).

Reading — the result is split, and the split is the informative part. The pre-registered primary signature (STRICT) does not externally validate: its C-index interval includes 0.50, the log-rank test is null, and the adjusted HR of 1.280 per SD is the correct direction but a non-significant trend (p = 0.085). **PAN-HNSC — the only signature that survived internal validation (S9.2) — does discriminate** on the continuous score, with an interval excluding 0.50 and an adjusted HR of 1.630 per SD (p = 0.0034), which holds after adding stage and treatment (1.665, p = 0.0052). **BROAD, null internally, is null externally.** The two standardisation conventions agree closely (Spearman ϱ = 0.986 between the PAN-HNSC z and raw scores; ϱ = 0.565 between STRICT · z and PAN-HNSC · z), so the conclusion does not rest on that choice. The PAN-HNSC adjusted p survives Bonferroni correction over all six cells (0.020) and over the three distinct signatures (0.010).

Three constraints are stated with the result, and none is optional. (1) **No risk-group claim is supported.** The median-split log-rank test is non-significant for every cell, PAN-HNSC included (p = 0.067): the prognostic information is in the continuous score and dichotomising discards it. (2) **We do not promote PAN-HNSC to primary.** Figure S1A designates STRICT the pre-registered primary and PAN-HNSC a legacy sensitivity variant; swapping them after seeing which survives would be outcome-dependent selection, doubly so

because PAN-HNSC was already the variant that survived internally. (3) **Scope mismatch.** GSE41613 is oral-cavity and HPV-negative, matching STRICT's scope, whereas the signature that validated was fitted on the anatomically unrestricted, partly HPV-positive cohort — a transfer *across* scope.

What this does not interact with. The 100 % false-positive rate under label permutation (S9.2) is a property of the *selection procedure*. Nothing was selected here: the coefficients were frozen and applied once, so this is a single clean test and that finding neither discounts it nor rescues the withdrawn in-sample claims.

Power. At 97 patients and 51 events the lower bound of the PAN-HNSC C-index interval is a discrimination too weak to be clinically useful; equally, the STRICT null excludes a large effect, not a small one. One deposit, one platform, one centre.

For context, not replication. The companion study [13] externally validated **its own**, different signature in this same deposit and reported C = 0.57, log-rank p = 0.18 and a multivariable HR of 1.24 per SD (0.95–1.61, p = 0.11). The two signatures share neither anchor panel nor feature pool nor fitting cohort; the only shared object is the validation cohort. Source: `results/parquet/r1_corrected/L7_external_validation_gse41613.csv` (per-patient strata in `L7_external_gse41613_km.parquet`; full report in `E7_EXTERNAL_VALIDATION_REPORT.md`).
